# Neotropical cloud forests and páramo to contract and dry from declines in cloud immersion and frost

**DOI:** 10.1371/journal.pone.0213155

**Published:** 2019-04-17

**Authors:** E. H. Helmer, E. A. Gerson, L. Scott Baggett, Benjamin J. Bird, Thomas S. Ruzycki, Shannon M. Voggesser

**Affiliations:** 1 International Institute of Tropical Forestry, United States Department of Agriculture, Forest Service, Río Piedras, Puerto Rico, United States of America; 2 Ecological Research Support, Houghton, Michigan, United States of America; 3 Rocky Mountain Research Station, United States Department of Agriculture, Forest Service, Fort Collins, Colorado, United States of America; 4 Center for Environmental Management of Military Lands, Colorado State University, Fort Collins, Colorado, United States of America; Universitat Trier, GERMANY

## Abstract

Clouds persistently engulf many tropical mountains at elevations cool enough for clouds to form, creating isolated areas with frequent fog and mist. Under these isolated conditions, thousands of unique species have evolved in what are known as tropical montane cloud forests (TMCF) and páramo. Páramo comprises a set of alpine ecosystems that occur above TMCF from about 11° N to 9° S along the Americas continental divide. TMCF occur on all continents and island chains with tropical climates and mountains and are increasingly threatened by climate and land-use change. Climate change could impact a primary feature distinguishing these ecosystems, cloud immersion. But where and in what direction cloud immersion of TMCF and páramo will change with climate are fundamental unknowns. Prior studies at a few TMCF sites suggest that cloud immersion will increase in some places while declining in others. Other unknowns include the extent of deforestation in protected and unprotected cloud forest climatic zones, and deforestation extent compared with projected climate change. Here we use a new empirical approach combining relative humidity, frost, and novel application of maximum watershed elevation to project change in TMCF and páramo for Representative greenhouse gas emissions Concentration Pathways (RCPs) 4.5 and 8.5. Results suggest that in <25–45 yr, 70–86% of páramo will dry or be subject to tree invasion, and cloud immersion declines will shrink or dry 57–80% of Neotropical TMCF, including 100% of TMCF across Mexico, Central America, the Caribbean, much of Northern South America, and parts of Southeast Brazil. These estimates rise to 86% of Neotropical TMCF and 98% of páramo in <45–65 yr if greenhouse gas emissions continue rising throughout the 21^st^ century. We also find that TMCF zones are largely forested, but some of the most deforested areas will undergo the least climate change. We project that cloud immersion will increase for only about 1% of all TMCF and in only a few places. Declines in cloud immersion dominate TMCF change across the Neotropics.

## Introduction

### Background

**Fig 1:**
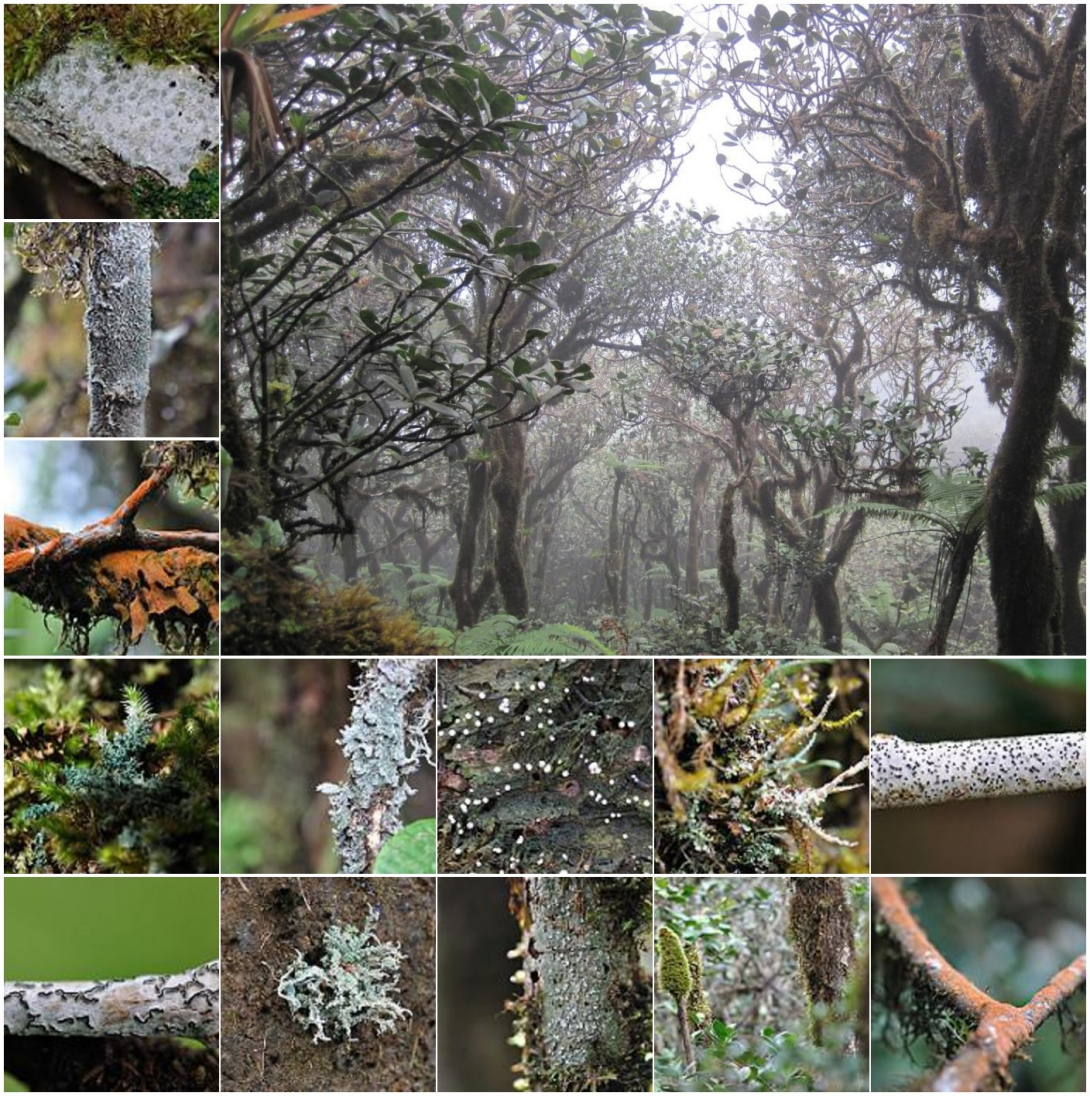
TMCF species adaptations reflect persistent cloud immersion. In Puerto Rico, for example, high lichen diversity is illustrated for stunted, ridgetop “elfin” cloud forest (photos by Joel A. Mercardo-Díaz and María Rivera).

Clouds persistently engulf many tropical mountains at elevations cool enough for them to form, creating isolated patches of habitat with markedly more fog and mist compared with lower elevations. Under these conditions, thousands of unique species have evolved in what are known as tropical montane cloud forests (TMCF) and páramo, a set of alpine ecosystems above some TMCF. All continents and island chains with tropical latitudes and mountains have TMCF. Páramo occurs in the Neotropics from about 11° N to 9° S along the continental divide. TMCF are among the most biodiverse ecosystems on Earth [1]. Amid this diversity, endemic species, those found nowhere else, are more common in TMCF than in other tropical forests [2–6]. The highest endemism of birds and mammals on Peru’s megadiverse eastern Andean slope is in cloud forests [6]. Among the important ecosystem services that TMCF provide, cloud forest trees, and the abundant and diverse epiphytes living on them, like mosses, ferns, bromeliads and lichens (Fig 1), intercept atmospheric water vapor that can amount to 75% of the stream water in drier places [7]. Patterns of species richness and endemism in TMCF are spatially complex, include many restricted-range species, and vary among taxa [6].

**Fig 2:**
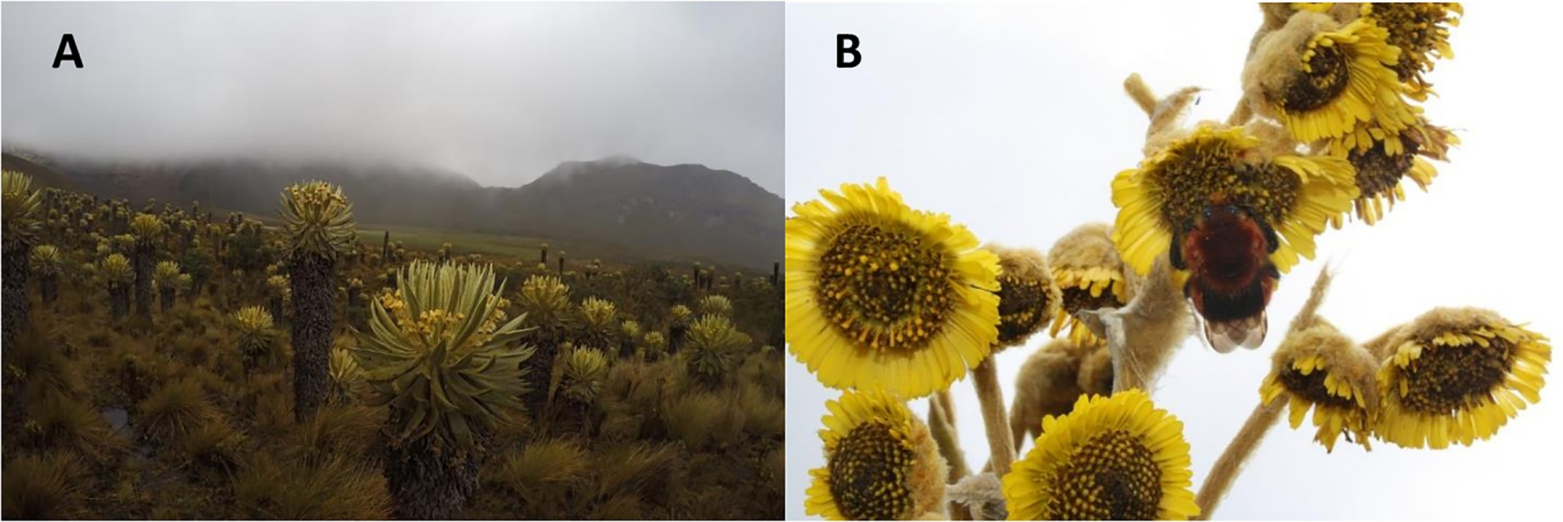
Many páramo species are adapted to frost and scavenge fog water. (A) Large rosette plants (*Espeletia* sp.) characteristic of much páramo vegetation and (B) a closeup of an *Espeletia* sp. showing its dense pubescence (photos by Mike and Lara Wolf (A) and Felipe Rodríguez (B)).

Páramo ecosystems occur in the equatorial Andes and Southern Central America. They are the headwaters for the Amazon River and critical to Andean water supplies [8]. With high plant diversity, páramo host ~4700 plant species [9]. A comparable extent of temperate alpine vegetation, in the southern Rocky Mountains of North America, has about 580 plant species [10], a much less diverse flora. Dominated by tussock-forming grasses, hard-leaved evergreen shrubs and large rosette plants (Fig 2A), páramo vegetation has many adaptations to cope with variable temperatures and intercept water vapor, including dense pubescence (Fig 2B) [9]. Geographic isolation and species endemism intensify in páramo. Páramo “islands,” *i*.*e*., patches of páramo habitat surrounded by cloud forest at lower elevations, are higher up on mountaintops. Páramo patch isolation was recent in evolutionary terms, because the Andean uplift to alpine elevations is geologically recent. Being isolated and geologically young, new plant species are evolving in páramo faster than anywhere else on Earth [11, 12]. Like island archipelagos, páramo bird species richness increases with páramo patch size, and endemism increases with distance from the geologically oldest patch [13]. Sixty-nine bird species are restricted to páramo [14]. To understand páramo species vulnerabilities, given these patterns of diversity, information on projected climate changes is needed at spatial scales much finer than the scales of global climate models. Most páramo areas are lost in the large grid cells of global climate models (GCMs).

Concerns are mounting that both climate and land-use change are affecting TMCF cloud immersion and species distributions [2, 15–27]. Similar concerns exist for páramo [22, 28, 29]. The fog and mist characterizing TMCF and páramo form from warm, moist air that rises by: 1) orographic uplift, in which prevailing winds force air against mountains and upwards; and, 2) convection, in which the land surface emits absorbed solar radiation, heating the impinging air as it rises [2]. Warmer ambient air temperatures could mean that rising air must travel further up mountains before it cools enough to form clouds, thereby reducing TMCF humidity or area. Reduced humidity negatively affects the plants and animals that are uniquely adapted to the high RH of cloud forests [19, 21, 30], and may make cloud forests more susceptible to fire [27]. Water vapor interception is a critical water source to some TMCF trees [30] and páramo plants [28]. Reduced cloud immersion and rainfall, and increased temperatures, may also reduce soil moisture and carbon storage and change plant species functional types toward faster-growing species. Increased global nitrogen deposition may be exacerbate these impacts [31].

Earth’s climate is changing quickly, while human demands for fresh water and land increase. There may be options to stem losses of TMCF and páramo species and their contributions to people [20]. Exercising those options requires planning based on knowing, at scales meaningful to management, where and how the climate variables that most uniquely define TMCF and páramo will change. These fundamentals, however, are largely unknown [16, 23]. Climate simulations or monitoring at a few places, and coarse-scale global climate simulations, project decreases in cloud immersion or low-level cloudiness in some places with TMCF, but increases in others, or only small changes [16–18, 23, 24]. Although global climate models (GCMs) simulate atmospheric climate profiles, their grid cells are 0.5° or larger. These resolutions are too coarse to assume that projected climates for cells containing cloud forest or páramo are what those ecosystems will experience [2, 16, 23]. Climate changes with elevation over distances shorter than each cell.

Even mapping current TMCF or páramo zones with the spatial detail needed has been a major challenge [22, 28, 29], as has mapping TMCF or páramo cloud immersion. Factors interact dynamically to determine the elevation where clouds form, like the changing moisture content of rising air and differing elevation profiles of wind speed, temperature and aerosols [16]. Consequently, simple thresholds in temperature, rainfall, elevation, or satellite image-based cloud cover alone cannot delineate TMCF zones. Their ranges in these variables are broad [7, 32] and overlap with non-TMCF zones. A point database of cloud forest locations from the World Conservation Monitoring Center (WCMC) [33] illustrates the wide range in cloud forest climates. Excluding extremes, these TMCF points range in mean temperatures from 12 to 24 °C, in rainfall from 800 to 3400 mm yr^-1^ and in elevation from 400 to 2800 m [32]. One source of this variability is the mass elevation effect: larger mountains absorb more solar radiation and emit more heat to rising air, increasing the elevation of cloud condensation.

No prior work maps TMCF cloud immersion metrics and extent at high spatial resolution across continents for both current and projected future climates (S1 Review and S1 Table). Regional Climate Models (RCMs) [17, 26] have only regional or smaller spatial extents, and GCMs cannot resolve mountaintops. Gauging cloudiness with discrete elevation thresholds or models using satellite image-based cloud cover [34–36] does not predict cloud forest conditions or extent for changed climate. Parameterizing empirical models with current maps of TMCF and climate to project future TMCF extent [20, 22] is limited to where vegetation maps exist that distinguish cloud forest from other forests. Most vegetation maps do not distinguish TMCF [7]. Finally, some prior work underestimates TMCF on small insular and coastal mountains while overestimating it on large dry mountains [35, 36]. The limitations of these prior works meant that mapping current and projected TMCF extent and cloud immersion across continents, at high resolution, required a new empirical approach.

### Objectives

Our overarching goal is to find where, and in what direction, cloud immersion of TMCF and páramo zones may change with climate across the Neotropics. To address this question and its implications for sustainability, we developed the objectives below.

1. Develop a new approach to mapping TMCF and páramo across continents that reveals how climate change will affect their distribution and cloud immersion.
2. Use the approach to map current and projected TMCF and páramo distributions across the Neotropics. Then, compare the projected differences in cloud immersion with those where field studies in TMCF show upward species migrations or cloud immersion changes. In addition for páramo, estimate how climate change will affect the extent and number of contiguous areas of páramo habitat (*i*.*e*., patches).
3. Evaluate the implications of the above results for TMCF protection, restoration and monitoring by: (a) comparing the changes in cloud immersion in protected *vs*. unprotected TMCF zones; (b) quantifying forest cover in TMCF zones, including overall and in protected and unprotected areas; (c) quantifying projected changes in cloud immersion metrics by both forest cover and protection status; and (d) comparing the proportions of currently deforested TMCF zones with those projected to undergo declines in cloud immersion. Although this last comparison does not account for future deforestation, it can shed light on the question of whether land-use or climate changes pose more risk to TMCF and páramo ecosystems.

Given the high species endemism of TMCF and páramo, we summarize results by ecoregions [37] and hierarchical groupings of them, because ecoregions consider species assemblages. We also consider projected changes in cloud immersion by the type of vegetation found at TMCF upper limits.

As we describe below, the new approach to mapping TMCF uses metrics for cloud immersion and frost. It can project these metrics under climate change scenarios at a scale appropriate for management. It relies on a novel empirical model to map the first cloud immersion metric, cloud forest minimum elevation (CF_min_). This empirical model includes novel application of maximum watershed elevation (Elev_max_), and it estimates mountain-base RH in a new way, by averaging watershed-level RH at 150 m elevation. Regional adjustment plus Elev_max_ allow the model to for the first time explicitly address the effect of mountain size on CF_min_, detecting coastal and insular TMCF. Also uniquely, we use frost metrics to define TMCF upper limits and páramo lower limits. To parameterize the CF_min_ model, we assembled a dataset of TMCF minimum elevations, from which we also obtained thresholds for the second cloud immersion metric, minimum relative humidity (RH_min_).

## Materials and methods

### Overview of TMCF and páramo mapping

We conducted a detailed review of related studies (see S1 Review and S1 Table). We determined that the new approach to TMCF and páramo mapping was needed based on that review.

The new method for mapping TMCF and páramo zones uses thresholds in two cloud immersion metrics and frost. To qualify as TMCF, cells had to: 1) be above a minimum elevation, CF_min_, which we modeled with multiple regression and a dataset of CF_min_ points; 2) have mean hourly relative humidity (RH) above subregional minima (RH_min_); and 3) have less frost than subregional frost thresholds that distinguish páramo and other higher-elevation vegetation from montane, subalpine and mixed TMCF. CF_min_ delineates the lower elevation where the cloud bank forms often enough for cloud forest to occur, RH_min_ eliminates places above CF_min_ that are too dry to support TMCF, and the frost thresholds map TMCF upper limits. To qualify as páramo, cells had to meet the above two cloud immersion criteria, be above TMCF upper limits and occur in the region where páramo occurs in the ecoregion map. To be above TMCF upper limits, cells had to have as much or more frost than subregional frost thresholds. The páramo region includes the Andes from about 11° N to 9° S and the Talamancan Mountains, which are in Costa Rica and Panama, and it includes the drier vegetation that is now also known as Jalca [9, 37]. We mapped all of these zones at a resolution of 7.5 seconds (~250 m), corresponding to the DEM [38] that we used to estimate and map CF_min_.

#### Threshold for cloud forest minimum elevation (CF_min_)

For the first threshold to map cloud forests, we parameterized a multiple regression model to map CF_min_ (Eq 1):

$${\text{CF}}_{\text{min}}=a_{1}+b_{1}{\text{ELEV}}_{\text{max}}{}^{2}+b_{2}{\text{ELEV}}_{\text{max}}+b_{3}{\text{RH}}_{150}+b_{R2\text{-}R5}\text{Region}$$ (1)

**Fig 3:**
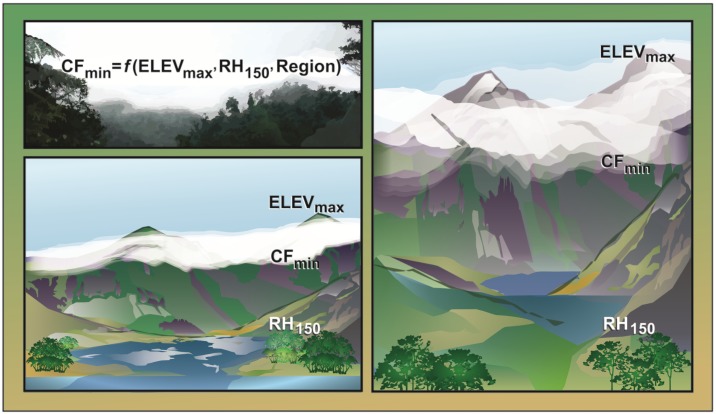
A simple model mapped and projected cloud forest minimum elevation (CF_min_). The model parameters include maximum watershed elevation (ELEV_max_), to gauge the mass elevation effect; average annual hourly relative humidity (RH) from 100–150 m elevation within a watershed (RH_150_), to account for differences in the humidity of the rising air; and region (Region), to help account for other factors that affect cloud formation. All else equal, the mass elevation effect causes CF_min_ to be at a lower elevation on smaller mountains compared with larger ones. Also, on larger mountains, frost or atmospheric inversions occur that cause colder or drier conditions that define cloud forest upper limits, though fast-draining soils, land use, microclimate, or other factors may also define cloud forest upper limits.

Where CF_min_ is the elevation at the minimum cloud forest elevation, from 79 coordinates representing CF_min_; ELEV_max_ is the maximum elevation of the surrounding watershed; RH_150_ is the mean RH of cells from 100–150 m elevation in the watershed, from a map of RH; Region is a discrete variable indicating a region (Fig 3, S2 and S3 Tables); and *a*_*1*_, *b*_*1*_*-b*_*3*_, and *b*_*R2*_-*b*_*R5*_ are constants. The model rationale relies on well-recognized but previously unquantified relationships between CF_min_ and mountain size, initial RH of rising air, and Region, which gauges other factors affecting CF_min_ (see below). We expected that ELEV_max_ would explain much of the variability in CF_min_ both within and among regions, because it explicitly accounts for the mass elevation effect, *i*.*e*., the positive effect of mountain size on CF_min_. All else equal, clouds form at higher elevations on larger mountains. Similarly, we expected that RH_150_ would help explain variability in CF_min_ within and among regions, because all else equal, clouds form at lower elevations where the rising air is more humid [2]. We used RH_150_ instead of sea-level RH because in continental regions like the eastern slopes of the Andes, cells at sea level are far from the mountains where cloud forests occur and do not represent the moisture content of rising air.

#### Response data for the CF_min_ model

To parameterize the CF_min_ model, we developed a dataset of coordinates for points representing 79 estimates of CF_min_ from across the Neotropics. The estimates are mainly from the literature (S2 and S3 Tables). The elevations where tropical forests transition to TMCF can be gradual or abrupt [2]. Frequent clouds and mist visibly change tropical forest species composition [39, 40], soil characteristics [41] and vegetation physiognomy [1]. Many observations of the elevation of this change are published. We compiled 66 estimates of CF_min_ published in 54 ecological studies (S3 Table). These estimates of CF_min_: 1) were in a peer-reviewed ecological journal article or book chapter; 2) explicitly stated an elevation or range of elevations where forest transitions to cloud forest, or delineated cloud forest boundaries in a map based on field work; and 3) included information clarifying that the cloud forest lower boundary (a) included the lowest-elevation cloud forest present (often lower montane TMCF), meaning that it was not limited to shorter or stunted cloud forest where taller cloud forest was also present (*i*.*e*., the definition of TMCF was not limited to upper montane, elfin, or subalpine cloud forest), (b) did not include lower-elevation evergreen forest that was not cloud forest, and (c) included either a description of changes in forest physiognomy or species composition indicating a transition to cloud forest, or a map legend clarifying what cloud forest lower boundaries represented. If a study reported an elevation range for the transition to cloud forest, or if more than one study estimated CF_min_ for a site, we averaged the reported elevations. Using Google Earth and the DEM, we identified a forested cell at the site of the authors’ field work with an elevation ≤ 50 m from the CF_min_ estimate.

We found no publications meeting the above criteria for the Pacific slope of the northwestern Andes and the northeastern slope of the Andes in Colombia. However, extensive cloud forests occur there. These latitudes are where the Intertropical Convergence Zone (ITCZ) spends the most time in the Neotropics, making them among the most humid in the Neotropics. We estimated CF_min_ values for these areas from remotely sensed evidence of persistent cloud cover at ground level. We selected representative points by overlaying the DEM with Landsat satellite imagery composited to minimize cloud cover [42]. In such imagery, residual cloudy patches shaped like the underlying topography indicate persistent cloud cover at ground level.

#### Relative humidity and mountain size for the CF_min_ model

The CF_min_ model required that we map RH_150_ and mountain size. To map RH, we assembled a Neotropical-wide dataset of hourly temperature and dew point temperature readings from 391 climate stations, including any climate station with four or more years of hourly or near-hourly data between the years 1945–2010, the same time span as the literature-derived observations of CF_min_. We estimated hourly RH from hourly temperature and dew point temperature [43]. We modeled and then mapped RH as a function of the bioclimatic variables in Worldclim data (S1–S3 Figs, S2 Table). Similar means have been used to map RH across the United States [44]. Watershed delineations for estimating and mapping ELEV_max_ and RH_150_ were mostly level-three watersheds from HYDRO1k [45]. We used finer-level HYDRO1k in the following areas where level 3 defined only one watershed: southern Central America, Cuba and Hispaniola. HYDRO1k defined only one watershed at all levels for central Amazonia, where we delineated smaller watersheds, and for other Caribbean islands, for which we generalized watersheds that were delineated from 30-m DEMs [46].

#### Region for the CF_min_ model

The discrete variable Region is included because cloud forests with the same ELEV_max_ and RH_150_ could still have different minimum elevations. Many other factors can affect the complex process of cloud formation, like coastal proximity, wind speed, aerosols, sea surface or ambient air temperatures [2, 7, 16], and dynamic changes in vertical profiles of air pressure or temperature. Contributing to variations among regions is that the annual mean humidity of cloud forests varies. “Cloud forest” refers to forest characterized by persistent clouds at ground level. However, cloud forest in one region may be on average drier than that elsewhere, decreasing CF_min_ [2]. For example, ground-level clouds may be thinner or persistent only seasonally. The Region variable helps account for these differences, as they tend to be shared within regions. In fact, Scatena et al. [34] grouped Neotropical cloud forests into six regions and mapped them by assigning one minimum elevation to delimit TMCF across each region. We modified the Regions in Scatena et al. [34], combining climatically similar areas with too few CF_min_ points to form their own category, based on well-known factors affecting cloud forest minimum elevations: latitude, coastal proximity, and humidity near CF_min_ as indicated by dry vegetation types just below TMCF. Accordingly, we combined Northern and Southern high-latitude points to a group of four points encompassing Northeastern Mexico and the Southern Andes, where conditions are cooler and drier (the *Outer Tropics*). Similarly, we combined the two points from the Galapagos Islands and coastal Ecuador with other coastal points with dry vegetation below TMCF, including parts of windward coastal Venezuela and the island of Saba (*Dry Coastal*). The remaining Caribbean Islands with TMCF close to a coast formed the *Caribbean Coastal* group (humid coastal areas). We grouped points from Pacific coastal mountains near the intertropical convergence zone (ITCZ), as they are coastal and have very humid conditions (*Pacific Coastal*). Points from Southeast Brazil were not significantly different from, and were combined with, remaining points from Southern and Central Mexico, Hispaniola, and Central and South America to form the *Inland Tropics*. We included Hispaniola with the inland tropics because its highest peak, where the most cloud forest occurs, is about as far from a coastline as some mountain ranges in the inland tropics. Two areas with neither CF_min_ literature estimates nor satellite image evidence of persistent cloud cover were included with the rest of the inland tropics.

### Threshold for minimum relative humidity (RH_min_)

For the second threshold, we used RH at the 79 CF_min_ locations to designate subregional values for RH_min_. Changes away from RH_min_ assess changes in fog or mist frequency that can affect cloud forest species [19, 21, 30] in places that are projected to dry but remain above CF_min_. RH_min_ also defined TMCF upper limits in Southeastern Brazil and other places.

The RH_min_ threshold was set to low values, of 50 to 60%, South of the Huancabamba Depression. Drier conditions and fine-scaled topography, characterized by long, narrow mountains, cause the spatial distribution of vegetation types to be finely-scaled [47]. This topography, combined with sparsely distributed climate stations and the interpolation component of climate mapping, cause lower RH values to dominate some TMCF. To distinguish cloud forest from drier vegetation, we performed an unsupervised classification of satellite imagery into evergreen forest zones *vs*. drier vegetation zones, classifying the bands available (red, near-infrared and the two shortwave infrared bands) in 30-m Landsat image composites from Hansen et al. [42]. These image composites minimize cloud cover by combining the clear pixels from many scene dates after normalizing or correcting them for between-date differences, improving visual distinction between deciduous and evergreen forest [48] and making simple image classifications reliable [49, 50]. They typically represent dry season conditions in the study area. Images are less cloudy then, and the evergreen forest zones are much greener than the drier vegetation zones. We verified the classification with fine-scale imagery viewable with Google Earth. The classification distinguished evergreen from drier zones even where forest was disturbed. After editing the few exceptions by hand, we used the classification to remove drier vegetation zones (included because of the low values we set for RH_min_) from TMCF.

### Thresholds for frost

Frost delimited cloud forest upper limits where RH minima did not define them. Frost refers to frequency of freezing temperatures in d yr^-1^, which we mapped from the climate station data as a function of the Worldclim variables (S2 and S3 Figs). Frost has been similarly mapped in the European Alps [51]. We used two frost thresholds to map three elevation zones, including: 1) montane TMCF, with little to no frost; 2) a transition zone to higher-elevation vegetation formations, which have some frost and that we refer to as subalpine or mixed TMCF; and 3) higher-elevation formations, with more frost. Montane TMCF included lower montane, upper montane and stunted ridgetop (“elfin”) (*e*.*g*., Fig 1) cloud forests. Subalpine or mixed TMCF encompassed mosaics with or transitions from TMCF to other, non-TMCF vegetation. Subalpine TMCF is also stunted and occurs where cloud forest transitions to páramo, or to the drier tropical alpine grasslands of the Andes known as puna. Puna grasslands are at elevations above TMCF south of about 9° S. Being further from the equator, rainfall is both lower and more seasonal than the páramo further north. Mixed TMCF refers to vegetation in transitional or patchy areas where TMCF occurs together with other vegetation at higher elevations that is not páramo or puna.

The ‘other vegetation,’ mentioned above, includes tropical alpine vegetation besides páramo and puna, or other forest types. The other tropical alpine vegetation includes the páramo-like vegetation of the Guyana highlands, the volcanic highlands of Guatemala and Central Mexico, on Pico Duarte in the Dominican Republic and the vegetation on isolated rocky outcrops within TMCF of the Serra Do Mar in Southeastern Brazil [9, 52]. The other forest includes: pine-oak forests of the Sierra Madre Occidental, pine forests of Hispaniola, and the Araucaria forests of Southeastern Brazil.

Frost thresholds for these zones came from visually interpreting fine resolution imagery (≤5-m pixel sizes), viewable with Google Earth, in conjunction with the map of frost frequency. We distinguished montane from subalpine TMCF with visual cues in Google Earth or the Landsat imagery indicating an open or stunted tree canopy.

### Validation

To validate the cloud forest mapping algorithm, we first evaluated the CF_min_ model, including its fit and whether relationships in the model were as expected [53]. Second, we used an independent dataset to estimate model sensitivity, the percent of known TMCF sites that the algorithm detected. We could not estimate model specificity, the percent of sites mapped as TMCF that are not TMCF (*i*.*e*., map user’s accuracy, or 100% minus commission error). Estimating specificity would require Neotropical-wide TMCF maps. Maps of TMCF are only available for Mexico (and used in [20]) and some Caribbean islands [49, 54, 55].

### Forest cover and protected areas

We estimated forest cover with a 30-m resolution tree cover dataset from Landsat imagery for the year 2000 [42], defining forest as having ≥10% tree cover. The United Nations Food and Agriculture Organization defines forest as having 10% or more tree canopy cover and an area ≥0.5 ha. Consequently, forest estimates here include young forest stands. Previous work shows that, of existing global land-cover or tree-cover cover maps, the most accurate forest cover is derived from a binary forest *vs*. nonforest classification of the Hansen et al. [42] data, when forest is defined as having ≥10% tree cover [56]. We estimated protected proportions of TMCF zones and forest with the World Database on Protected Areas [57].

### Future scenarios

Future scenarios included two Representative Concentration Pathways (RCPs) and time spans. RCPs correspond to projected changes in the Earth-atmosphere energy balance resulting from future scenarios with different levels of greenhouse gas (GHG) emissions and land-use change [58]. The two scenarios represent a moderate scenario, with GHG emissions stabilizing by the year 2040, followed by gradual decline (RCP-4.5), and a worst-case scenario of increasing GHG emissions throughout this century (RCP-8.5). Future times include the years 2041 to 2060 (~2050) and the years 2061 to 2080 (~2070). We refer to the resulting four projections as: RCP-4.5, ~2050; RCP-4.5, ~2070; RCP-8.5, ~2050; and RCP-8.5, ~2070.

We used projected climate maps downscaled to a ~1-km spatial resolution [59] from the Coupled Model Intercomparison Project Phase 5 (CMIP5) [60], resampling them to the ~250-m cell size of the DEM. To reduce the effects of known biases in CMIP5 model projections, we reviewed available studies that evaluated the ability of CMIP5 projections to simulate observed climate in the Neotropics. They showed that these climate projections commonly have dry annual precipitation (P) biases across the Amazon basin [61] and, except for the Hadley models, they incorrectly locate the ITCZ in Northern South America [62], where there are extensive cloud forests. There, an ensemble model would diffuse the location of the ITCZ, averaging cells that in one model are within the ITCZ with cells from another model in which the ITCZ is further north or south. Further, studies ranked the Hadley models, HadGEM-ES in particular, above others in simulating the climate of the Amazon [61, 62]. Moreover, this model can simulate climate in Southern South America, where we found only one comparative study [63]. For these reasons, we placed more confidence in climate projected by HadGEM2-ES [64] for South America, rather than an ensemble. Across the Caribbean basin, cold biases in mean land (T) and sea surface temperatures (SST) have improved with the CMIP5 models, but remain as bias [65]. Consequently, we eliminated outliers and little tested models [66] by eliminating the three lowest-ranking or least accurate models for P, T or SST based on five comparative studies [65, 67–70]. Eliminated models were nearly all outliers in more than one study. The elimination process left three GCMs, and we used a three-model ensemble for Mesoamerica (*i*.*e*., Mexico and Central America) and the Caribbean, averaging results from these models: CCSM4 [71], MPI-ESM-LR [72] and HadGEM2-ES.

### Data summaries

To gauge changes in RH and CF_min_, we compare ecoregional changes in these metrics to changes in ecoregions where field studies in TMCF show upward species migrations. The latter ecoregions include the Guanacaste/Tilarán ecoregion, where studies at Monteverde link mist frequency declines with upward migrations of animal species, and the Peruvian Yungas foothills, where tree species distributions are rapidly shifting upwards [73]. Given the RH decline (RH_d_) of -2.6% in a worst-case scenario (RCP-8.5) in the Guanacaste/Tilarán ecoregion, we report the areas of projected TMCF change in 3% increments of RH change. We then intersect the maps of current and projected changes in TMCF zones with maps of protected areas and forest cover. This intersection finds where increased TMCF protection, restoration or monitoring may be warranted. We focused results and discussion of forest cover and protection on TMCF zones where we project the least severe changes in cloud immersion. In Mesoamerica and the Caribbean, it is cells with an RH decline in the range -3% < RH_d_ < 0%. In South America, it is cells with RH_d_ ≥ 0%. In all areas, the most severe cloud immersion declines are cells changing to be below CF_min_, to have RH_d_ ≤ -3%, RH<Rh_min_, or a combination of those declines.

### Technological advances

This study achieves several methodological advances. By modeling cloud forest minimum elevations, relative humidity and frost, it gauges both cloud immersion and zone extent for both TMCF and páramo across continents at high spatial resolution for current and projected climates while explicitly accounting for the mass elevation effect on TMCF. It also uses finer resolution data to estimate some inputs, including a 250-m DEM to estimate CF_min_ and ELEV_max_, hourly climate station data to estimate RH and frost, and finer-scale, 30-m tree cover data when estimating forest cover.

## Results

### The TMCF mapping algorithm

#### The CF_min_ model

**Fig 4:**
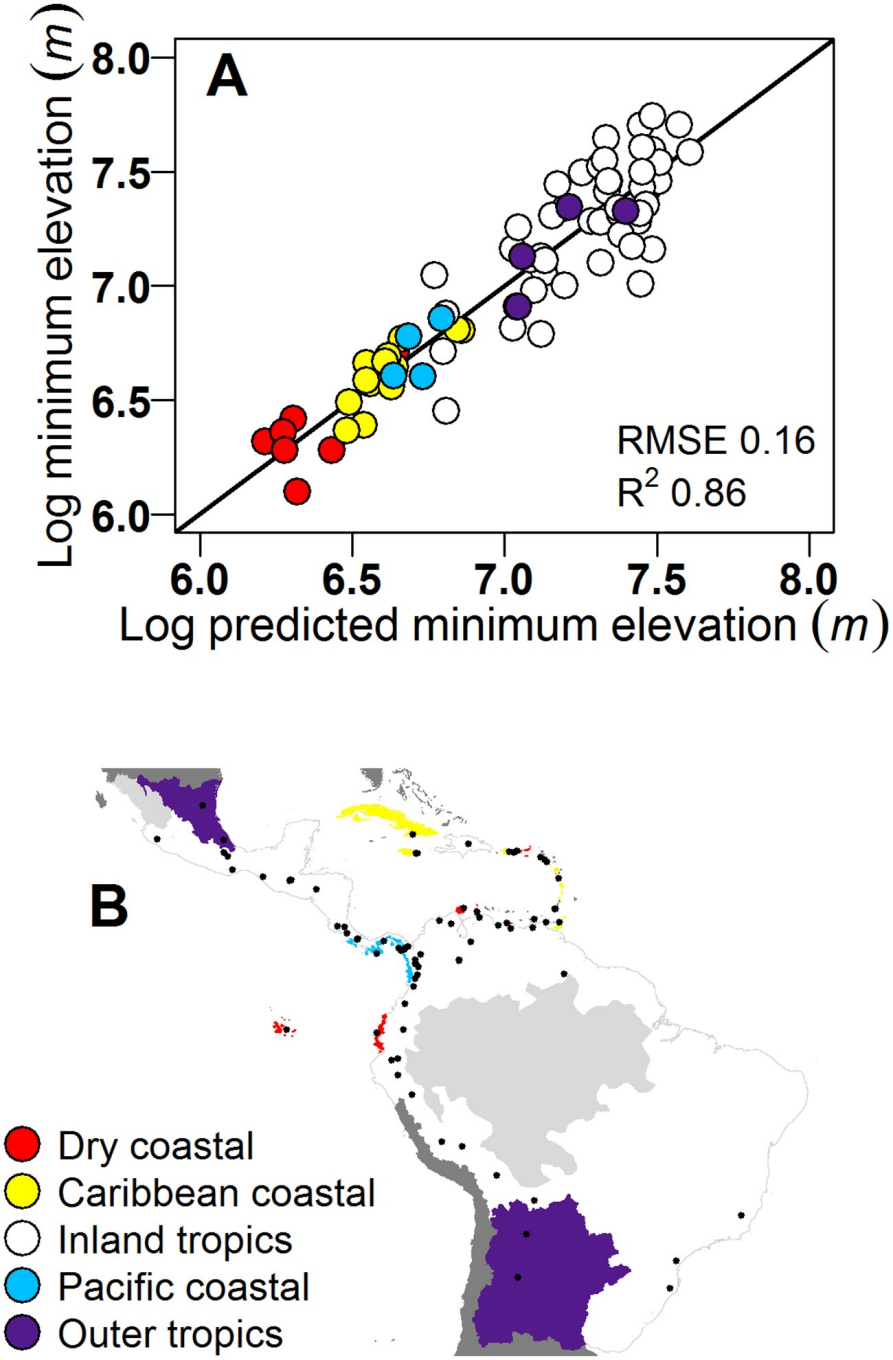
The CF_min_ model explained 86% of point variation without having bias. (A) A 1:1 line (thick black line) compared with log(CF_min_) plotted as a function of CF_min_ predicted by the mapping model: Log(CF_min_) = 8.480*** − 0.0305ELEV_max_^2^*** + 0.317ELEV_max_*** − 0.0233RH_150_*** − Region. Region = [0 if Region = Inland Tropics; else 0.676*** if Region = Dry Coastal; else 0.472*** if Region = Caribbean Coastal; else 0.324** if Region = Pacific Coastal; else 0.239*** if Region = Outer Tropics] (N = 79; Parameter estimate Pr > F: <0.0001 = ***, <0.01 = **), and (B) Modeling regions (Region). Light grey = areas with no CF_min_ estimates. Black circles = CF_min_ locations.

A plot of observed *vs*. predicted [74] points in the CF_min_ model explained 86% of point variation without obvious bias (Fig 4). Further, the model projected CF_min_ increases where field studies or dynamic climate modeling show or project rises in cloud LCLs or species elevation ranges (see next section). And as expected, CF_min_ lowers as RH_150_ increases. Predicting CF_min_ while holding ELEV_max_ constant at the mean value for all points in the model illustrates this trend (S4 Fig). The resulting graph, in S4 Fig, causes the points to appear more variable than they are in the actual model. Also as expected, CF_min_ rises with rising ELEV_max_, leveling off at higher elevations along with the distribution of mountain sizes, as illustrated by predicting CF_min_ while holding RH_150_ constant at its mean (in S4 Fig, CF_min_ is also more variable in this figure than in the model). Expected too was the result that for a given ELEV_max_ and RH_150_, CF_min_ was lower for coastal, island and outer-latitude points than for the rest of the Neotropics (S4 Fig).

#### Cloud forest detection

We estimate that TMCF zones in the Neotropics cover 401,370 km^2^, almost as large as the US State of California. To evaluate cloud forest detection sensitivity, we calculated the percentage of WCMC cloud forest sites (“known” sites) within five km of mapped (“predicted”) TMCF zones, following past work [35]. This percentage is akin to model sensitivity (*i*.*e*., map producer’s accuracy, or 100 percent minus omission error).

**Fig 5:**
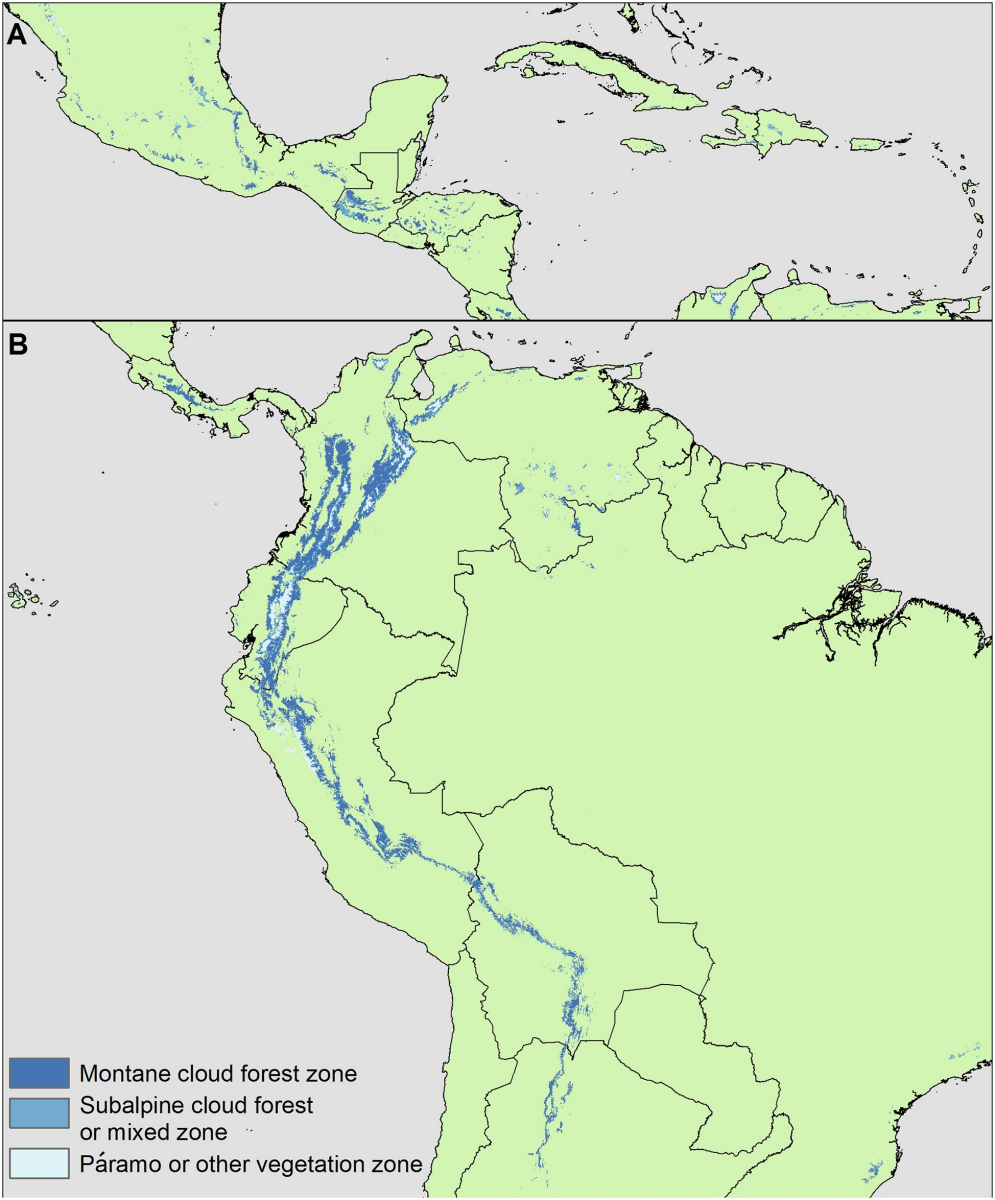
Current Neotropical montane cloud forests and páramo. The mapping algorithm, based on CF_min_, minimum annual relative humidity (RH _min_) and Frost, detected TMCF across the Neotropics, including (A) Mesoamerica and the Caribbean islands, and (B) South America. “Other vegetation” includes other tropical alpine vegetation or non-TMCF forest (see *Thresholds for frost*).

**Fig 6:**
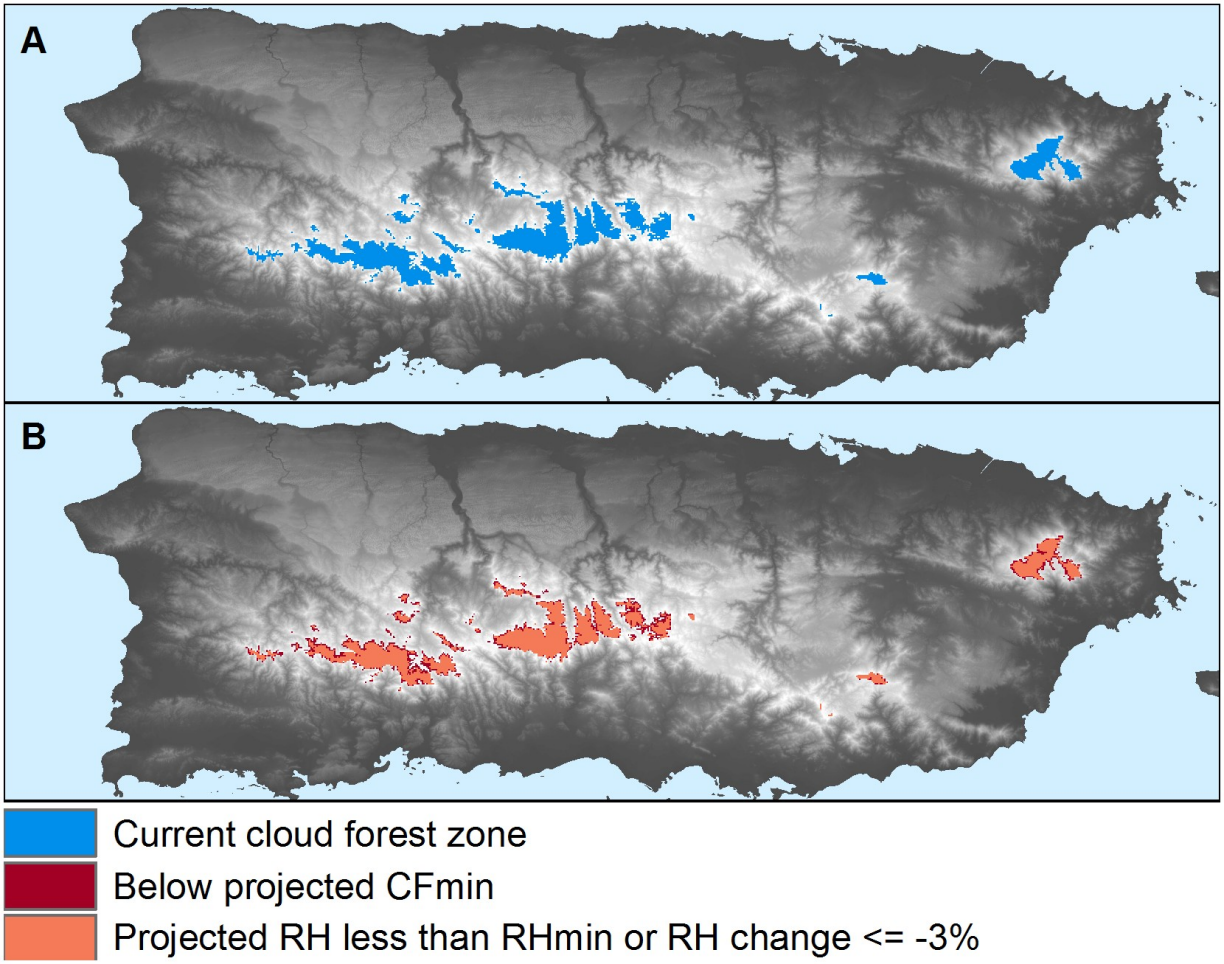
The approach detected TMCF on coasts and islands. A closeup example of coastal and island TMCF zones for the island of Puerto Rico for current (A) and projected future conditions (B). Under a moderate greenhouse gas emissions scenario (RCP-4.5), in <45–65 yr (~2070) all of Puerto Rico’s cloud forest zones would either be below CF_min_, suffer RH declines of 3% or more or to below RH_min_. Although not depicted in this figure, all of Puerto Rico’s TMCF zones would suffer RH drops of more than 3%, and these RH declines are largest in Western and Central Puerto Rico.

Here, 82% of Neotropical WCMC points were within five km of mapped TMCF, which is comparable to prior work [35] that detected 81% of global WCMC points within five km of mapped TMCF. Unlike the prior work, though, in this study the WCMC points are independent of the mapping algorithm (though some TMCF areas are represented in both the WCMC and CF_min_ datasets). In the prior work, the WCMC sites served both to evaluate and parameterize the mapping algorithm. If we estimate sensitivity with the points that we used to parameterize the mapping algorithm, 95% of them are within five km of mapped TMCF zones, and 80% are within 500 m. Further, the algorithm detected insular and coastal TMCF (Figs 5 and 6), which tend to be on small mountains, without mapping extensive cloud forest zones in the higher-elevation, inland savanna regions of Southeast Brazil (Fig 5). Unlike prior work, there TMCF only occurs about as far inland in the new map as points in the WCMC database, which is meant to comprehensively document all known TMCF sites.

In this study, most of the WCMC points that were further than 5 km from mapped TMCF zones were on small, isolated mountains in the Amazon basin (and far from the Andean foothills), or they were in Northwest Mexico. We did not find literature estimates of CF_min_ for these areas. Future work should include such estimates in model parameterization. In these regions, our maps are less reliable.

### Cloud immersion and frost changes

#### Neotropical changes in TMCF cloud immersion

**Fig 7:**
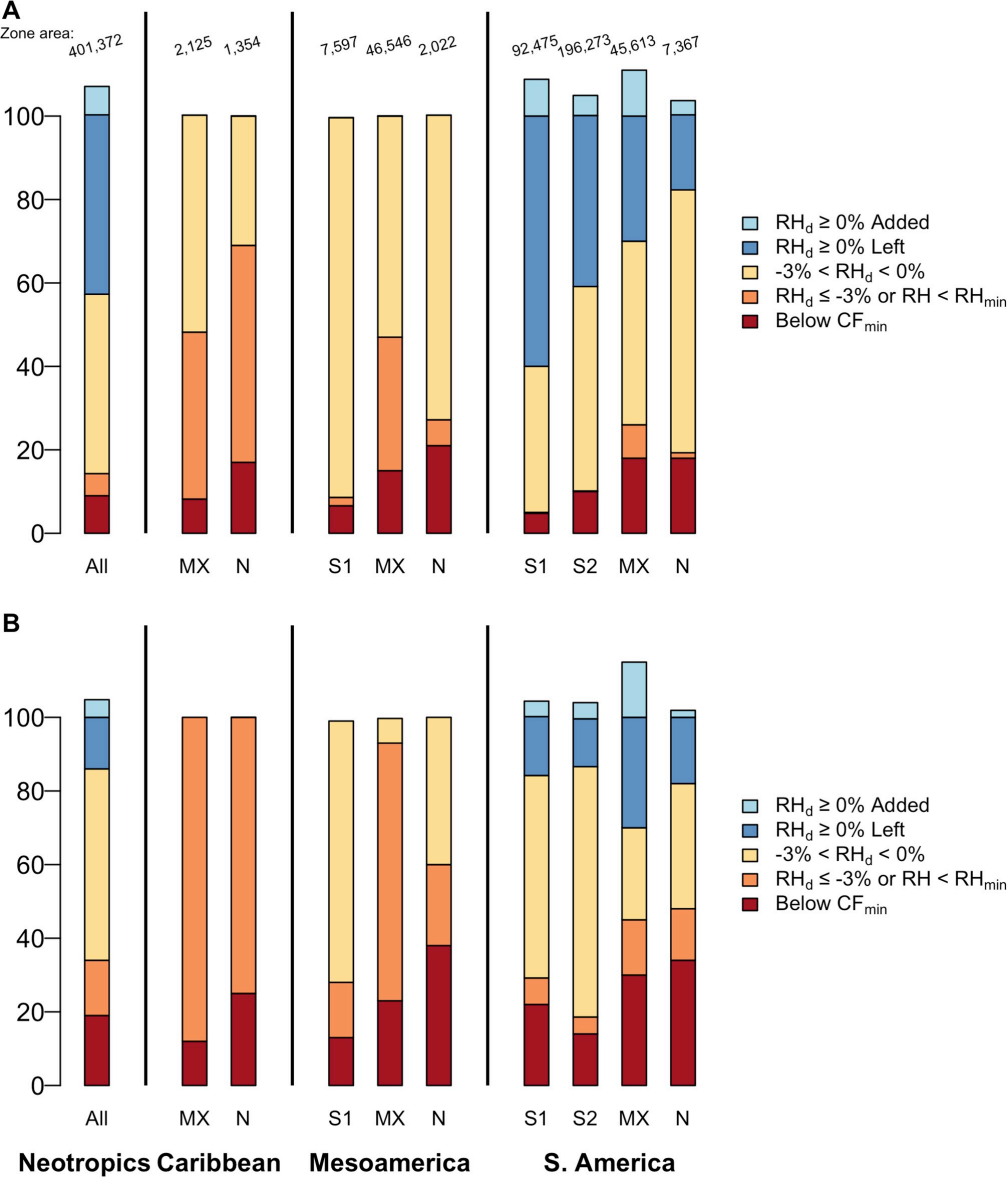
Cloud immersion changes in Neotropical cloud forests. Existing Neotropical TMCF zone area (in km^2^, top line, 250-m cell size) and percent of this area (bars) by Region, TMCF upper limit and category of change in cloud immersion. TMCF would experience cloud immersion declines of nearly 60 to 90% with (A) moderate climate change as soon as around 2040 (57% decline, RCP 4.5, average year 2050), to (B) a worst-case scenario as soon as around 2060 (86% decline, RCP 8.5, ~2070). The remaining area with a TMCF climate is 43% Left, plus 6.8% Added, ≈ 50% for (A) and 14% plus 4.8% ≈ 19% for (B). Most added TMCF comes at the expense of páramo. The total lost includes these *categories of change in cloud immersion*: **Below CF**_**min**_ (= CF_min_ rises above this area—other categories remain above CF_min_); **RH**_**d**_ **≤ -3% or < RH**_**min**_ (= RH falls severely); **-3% < RH**_**d**_ **< 0%** (= RH falls by up to 3%). The percent **Left** includes the category RH_d_ ≥ 0% (= RH is stable or increases slightly). The percent **Added** comes from CF_min_ lowering, RH increases, or frost declines at TMCF upper limits. *Types of TMCF at upper limits*: **No Subalpine** = montane TMCF where no subalpine TMCF occurs; **Mixed** = montane TMCF + mixed TMCF); **Subalpine 1 =** montane TMCF + subalpine 1 TMCF (TMCF transitions to páramo); **Subalpine 2** = montane + subalpine 2 TMCF (Subalpine 2 TMCF transitions to puna). *Regions*: S. America = South America, Mesoamerica = Mexico + Central America, Caribbean = Caribbean Islands, Neotropics combines the three regions.

In the four scenarios tested, almost 60 to 90% (57% to 86%) of existing Neotropical TMCF zone area (hereafter, TMCF unless otherwise indicated) experiences cloud immersion declines, as gauged by changes in CF_min_ and RH (Fig 7, S4–S7 Tables). This range includes 57%, reached as soon as around 2040 for the moderate, RCP-4.5 climate change scenario for the years 2041–2060 (average year 2050), to 86% as early as around 2060 for the worst-case, RCP-8.5 scenario (~2070). By ~2050 under RCP-8.5, more than 86,000 km^2^ of Neotropical TMCF, near the size of the U.S. State of Minnesota, would undergo the severest cloud immersion declines of being below future CF_min_ or RH_min_, or having an RH decline of three percent or more. This area of severe change rises to more than 136,000 km^2^ by ~2070, larger than the country of Greece. More than 48,000 km^2^ would be below CF_min_ for RCP-8.5 by ~2050, which rises to >75,000 km^2^, 19% of Neotropical TMCF, for RCP-8.5, ~2070 (Fig 7, S4–S7 Tables). Only small increases, of 1.3–1.4% of TMCF, are added from isolated CF_min_ lowering or RH increases. Only 3.4–5.5% increases in TMCF come from frost declines at TMCF upper limits (Fig 7, S4–S7 Tables).

The average 19% of all TMCF types across the Neotropics that would be below CF_min_ under RCP 8.5, ~2070, is smaller than if TMCF types and ecoregions are considered. Much larger portions of ecoregions of types N (small mountains with no alpine or subalpine zone) and MX (larger but drier mountains with mixed or non-TMCF higher-elevation zones) suffer the severest cloud immersion declines (Fig 7, S4–S7 Tables). All Caribbean and Mesoamerican ecoregions but one, the Talamancan Mountains, are types N or MX. On Caribbean islands without mixed (type MX) or alpine zones (*i*.*e*., all but Hispaniola), 25% of TMCF would be below CF_min_ in the worst-case scenario (RCP-8.5, ~2070). For Mesoamerica in the worst-case scenario, 38% of TMCF on small mountains (type N), and 23% on larger but drier mountains (type MX), would be below CF_min_.

#### Regional and ecoregional changes in TMCF cloud immersion

At the ecoregion scale, TMCF in about 90% of ecoregions both contracts and suffers RH declines as early as 2060 under RCP-4.5 (average year 2070) (S8 and S9 Tables). For Caribbean and Mesoamerican ecoregions, 100% of TMCF suffer these cloud immersion declines in all scenarios (S8 Table, which shows results for montane TMCF). The same is true, 100% of TMCF suffers declines, for the TMCF in many South American ecoregions, including those in northernmost South America, Alto Parana forests in Southeastern Brazil and the Bolivian Yungas (S9 Table). The northernmost South American ecoregions with 100% declines include Santa Marta, the northernmost parts of the Cordillera Oriental and most non-Andean ecoregions there including the Guyana highlands. Further, 90% to 99% of montane TMCF suffers these cloud immersion declines by ~2070 under RCP-4.5 in the following South American ecoregions: Andean foothills ecoregions, the Venezuelan Andes, and the Serra do Mar coastal forests (S9 Table). In addition, Pacific-influenced ecoregions have smaller RH drops (S8 and S9 Tables).

Net expansion of TMCF is only projected for the Galapagos Islands, Ecuadorian Dry Forests, along the western Andean slope south of the equator, in the southernmost Andean Yungas, in the Araucaria forests of Southeastern Brazil, and in some coastal locations in northern South America (S9 Table). In these places, expansion would occur from lowering of CF_min_ or increases in RH. Most of these ecoregions have a strong Pacific Ocean influence. The total areas of these expansions are small.

**Fig 8:**
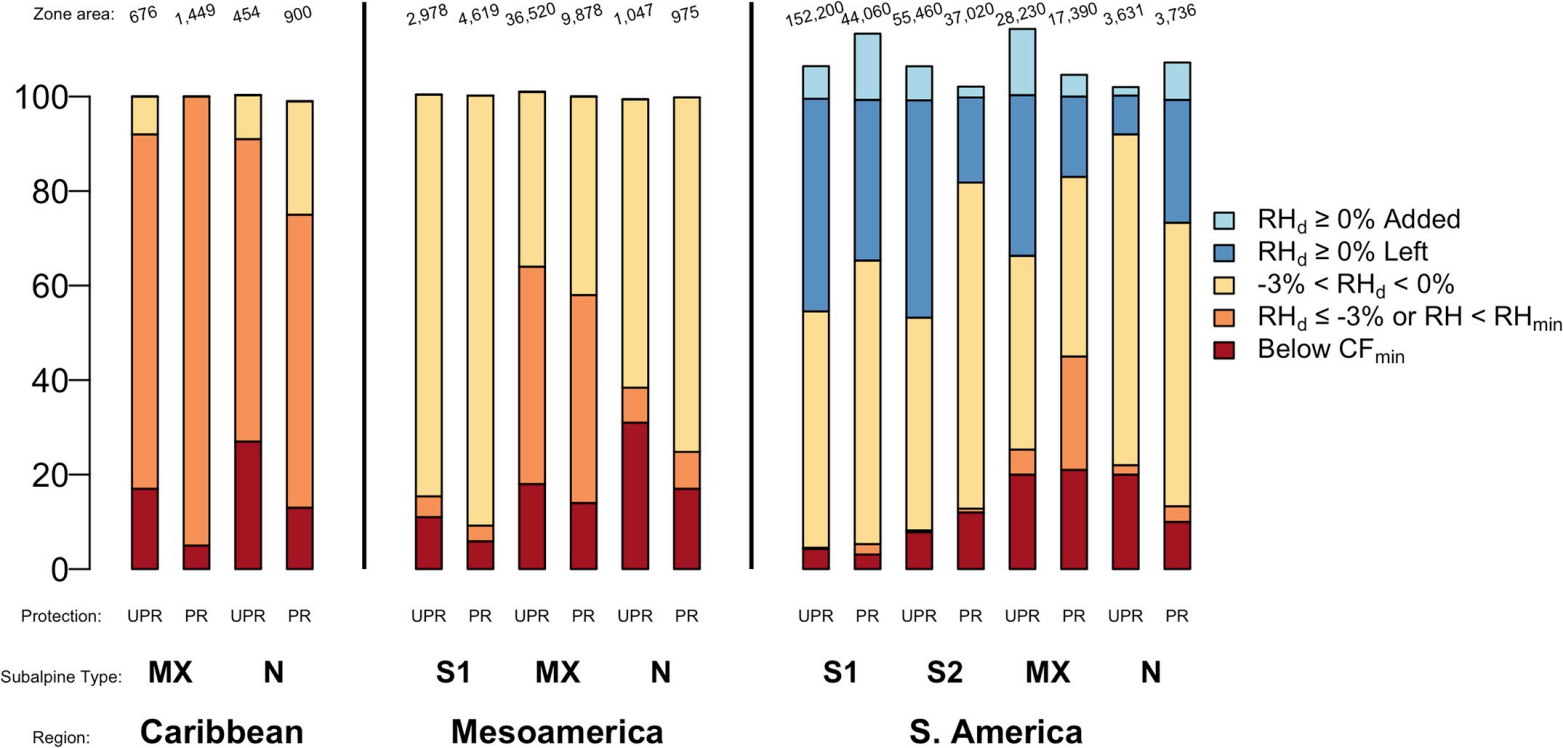
Cloud immersion changes by protection for RCP 4.5, ~2070. Existing Neotropical TMCF zone area (in km^2^, top line, 250-m cell size) and percent of this area (bars) by Region, TMCF upper limit, protection status and category of change in cloud immersion. In the Caribbean and Mesoamerica, unprotected and usually less-studied TMCF would experience greater cloud immersion declines. Protection status: **UPR** = Unprotected, **PR** = Protected. Types of TMCF at upper limits: Fig 7 legend defines cloud immersion change categories and other chart abbreviations.

Changes in cloud immersion differed by protection status in many places (Fig 8, S10–S13 Tables). In the Caribbean, Mexico, and Central America, and on smaller mountains (Type N) in South America, rising CF_min_ elevations affect much larger portions of unprotected TMCF than affect protected areas. Protected portions of TMCF in the Caribbean and Mesoamerica, and on small South American mountains, tend to be at higher elevations. In addition, the same increase in CF_min_ affects proportionally larger areas of small mountains compared to large ones. Along the Central and Northern Andes, CF_min_ rises above similar, or larger, portions of protected and unprotected TMCF. This result arises in part because many protected areas there extend from lowlands up to and often including TMCF.

#### Severity of cloud immersion declines

In a majority of ecoregions, projected cloud immersion declines are comparable to those where upward species migrations are linked to climate change. The montane areas of 20 out of the 24 ecoregions in the Caribbean and Mesoamerica exhibit RH declines steeper than the 1.5% drop observed for the Guanacaste/Tilarán montane forests ecoregion (S8 Table). There, upward animal species migrations are linked with reduced RH at the Monteverde cloud forest reserve [21], which is visited by tourists from around the world. In South America (S9 Table), 20% of TMCF ecoregions have RH drops larger than the 1.2% drop in the Peruvian Yungas foothills for this scenario (RCP-4.5, ~2070), where species are also shifting upwards [73]. These include Santa Marta, the Guyana Highlands and the Bolivian Yungas. In addition, the following ecoregions experience RH declines nearly as large, being ≥0.9%: the Venezuelan Andes, the Andean Eastern Cordillera Real foothills, and some non-Andean Northern South American ecoregions (S9 Table). As for CF_min_, by 2070 under RCP-4.5, it would rise above 34% of TMCF in Guanacaste/Tilarán and above 13% of TMCF in the Peruvian foothills. Comparable or larger portions of current TMCF would be below projected CF_min_ values in several ecoregions of the Caribbean and Mesoamerica, where six of 24 ecoregions would have ≥ 30% of their area below CF_min_ in the scenario. The same is true for 30% of South American ecoregions (S12 and S13 Tables). More than 50% percent of Caribbean and Mesoamerican ecoregions and 25% of South American ecoregions lose around 20% or more of their area to contraction from falling below CF_min_ for this moderate scenario, including TMCF in the Trans-Mexican Volcanic belt, where Monarch butterflies (*Danaus plexippus*) overwinter by the millions. They depend on cloud immersion there to provide relatively stable temperatures [75].

In four ecoregions of the Caribbean and Mesoamerica, 100% of montane TMCF is projected to be in the following most severe categories of cloud immersion decline under RCP 4.5, ~2070: (1) RH_d_ ≤ -3% or RH ≤ RH_min_, or 2) Below CF_min_ (S8 Table). They are Puerto Rico, the Windward Islands and Guadeloupe, Trinidad and Tobago, and Chiapas. Eighty to 97% of montane TMCF in five additional ecoregions also undergo these severe changes: the Leeward Islands, Hispaniola, Veracruz, Jamaica and the above-mentioned Trans-Mexican Volcanic Belt. In South America the following ecoregions have little to no montane TMCF area projected to have stable or increasing RH: Santa Marta, the Guyana Highlands, most other non-Andean Northern South American ecoregions, Alto Parana and the Campos Rupestres, and the Bolivian Yungas (S9 Table). More than 99% of Santa Marta TMCF are in the aforementioned severest change categories. This severe change extends to the Cerro Pintado, which is located in an adjacent ecoregion. In the following additional South American ecoregions, 80% or more of the current montane TMCF zone will experience RH declines and rising CF_min_: the Peruvian Yungas foothills, the Venezuelan Andes, the Serra do Mar and the Cordillera Oriental.

#### Changes in páramo cloud immersion and frost

**Fig 9:**
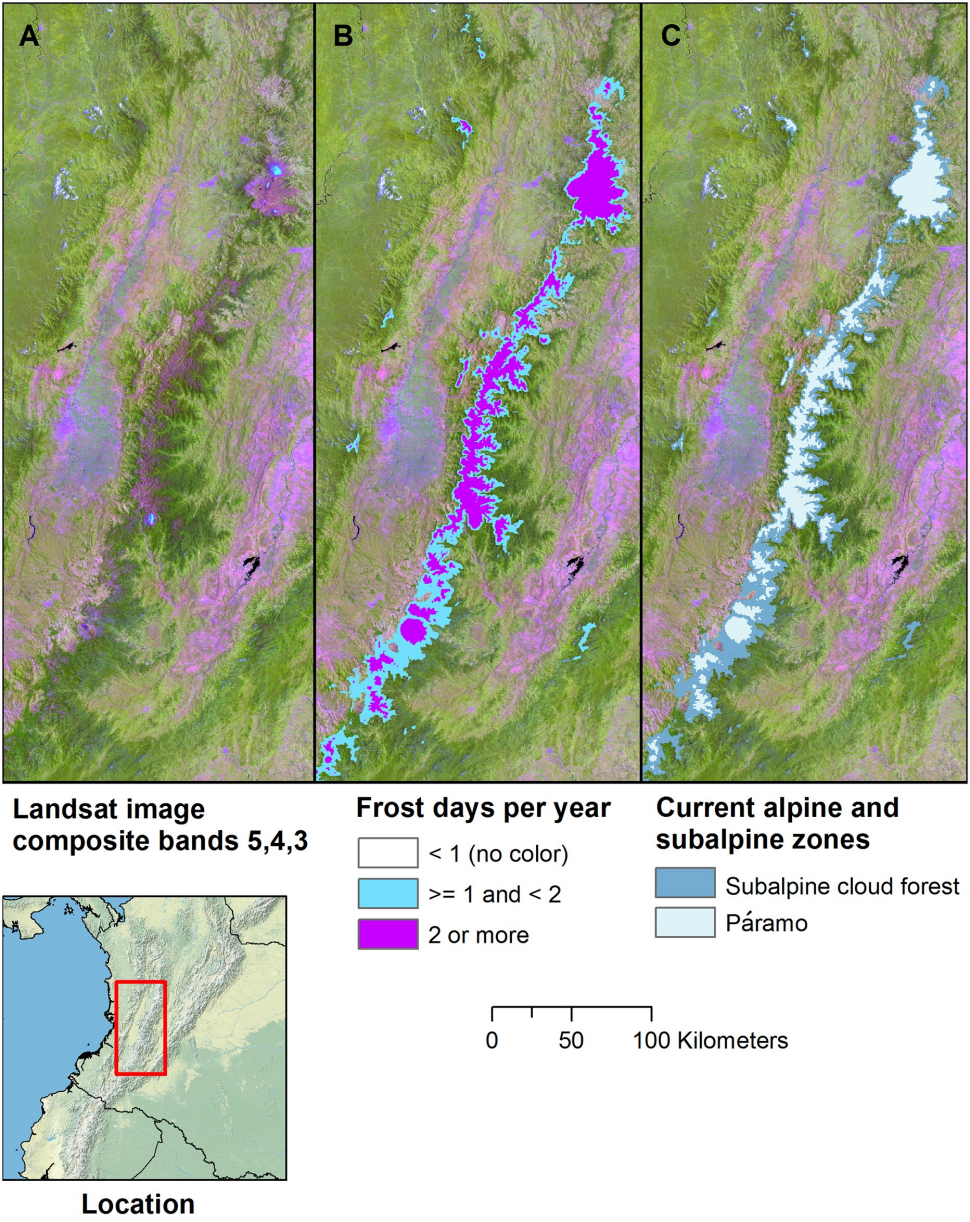
Transitions to páramo start at 1–1.5 d yr^-1^ of frost. Transitions to páramo from TMCF began at frost frequencies of 1 to 1.5 d yr^-1^, highlighting frost as a major factor delimiting equatorial Andean tree lines. (A) a Landsat image composite in which páramo appears in dark pink to magenta-green shades, subalpine TMCF appears in green-violet to green shades, and montane to lower montane TMCF is green; (B) areas exceeding minimum frost frequency thresholds for subalpine (*i*.*e*., transitional) or páramo zones; (C) how the frost frequency thresholds were used to map subalpine cloud forest and páramo zones. A frost day is any day that temperatures fall to ≤ 0° C.

Our results highlight frost as a major factor delimiting equatorial Andean tree lines. In defining TMCF maximum elevations, we found that where there are páramo ecoregions, the TMCF-to-páramo transition region (*i*.*e*., Subalpine 1 TMCF) begins where frost frequencies are at least 1 and up to 1.5 d yr^-1^, depending on the ecoregion. The transition continues to where frost frequencies reach 2 to 2.5 d yr^-1^. In the area shown in Fig 9, this transition, and consequently the extent of mapped subalpine TMCF, spans frost frequencies of 1–2 d yr^-1^. Frost frequencies that we used to delimit TMCF upper limits in ecoregions without páramo were larger (5 to 40 d yr^-1^), except for the Guyana Highlands. In addition, frost less precisely delineated transitions from TMCF to alpine zones in nonpáramo areas.

**Fig 10:**
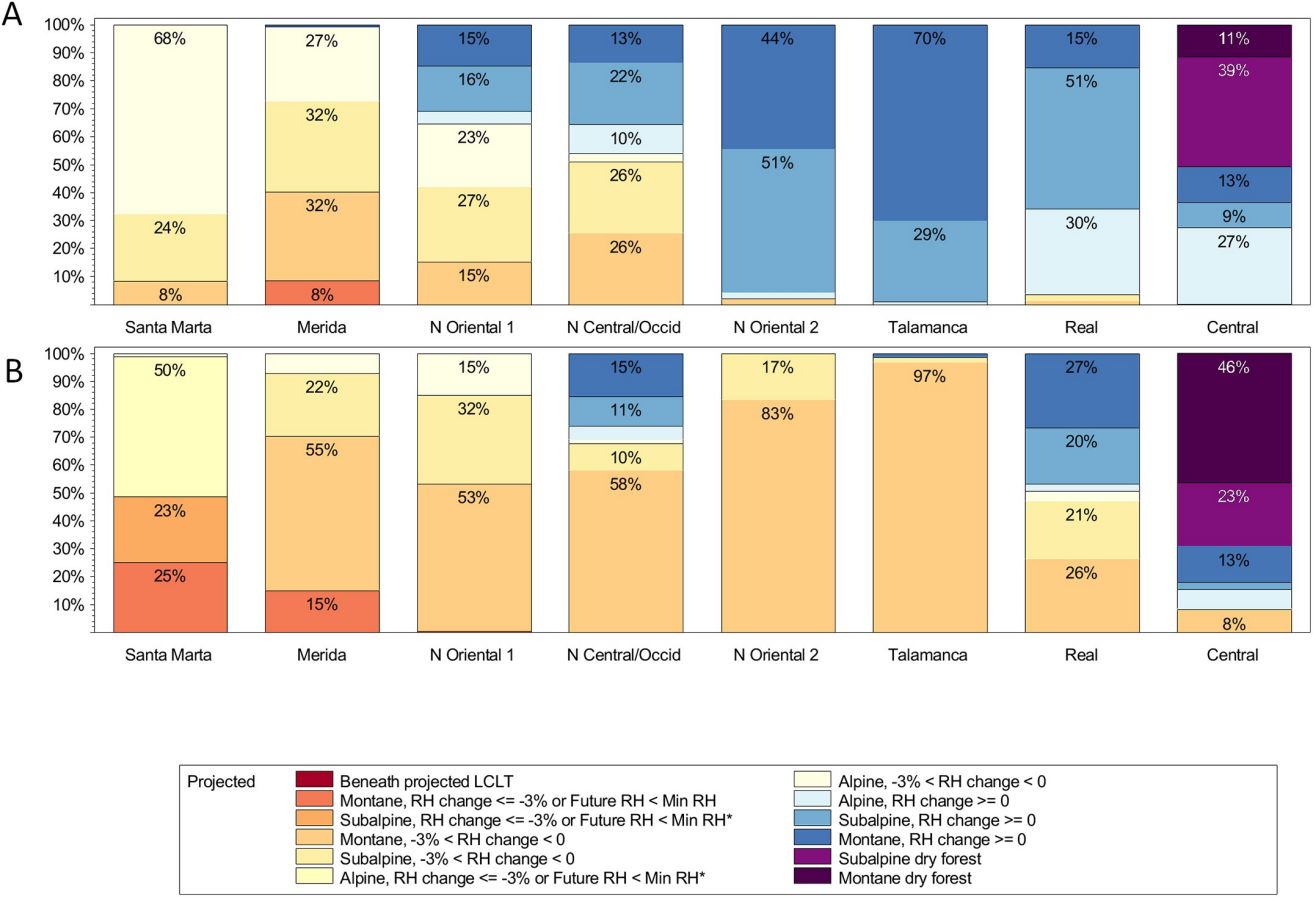
Ecoregional changes in páramo cloud immersion and frost frequency. Categories of change are shown as a percent of current páramo zone area for average year 2070 by Representative Concentration Pathway including: (A) RCP-4.5; (B) RCP-8.5. Páramo *ecoregions*: Santa Marta = Cordillera de Santa Marta; Talamanca = Cordillera de Talamanca (Costa Rica, Panama); N Oriental 1 = Colombian Cordillera Oriental north of 5° N; N Oriental 2 = Colombian Cordillera Oriental from 2° N to 5° N; N Central/Occid = Colombian Cordilleras Central and Occidental); Real = Ecuadorian Cordillera Real; Central = Peruvian Cordillera Central. *Frost change categories*: **Alpine** = frost above Alpine Frost_min2_; **Subalpine** = frost above Subalpine Frost_min1_; **Montane** = frost below Subalpine Frost_min1_. *Cloud immersion change categories*: **Beneath CF_min_** = falls below CF_min_; **RH_d_ ≤ -3% or < RH_min_** = RH falls 3% or more or below RH_min_; **-3% < RH_d_ < 0%** = RH falls up to 3%; **RH_d_ ≥ 0%** = RH is stable or increases.

Projections suggest that páramo will be strongly affected by climate change. Considering only frost reductions, as early as 2060 (~2061–2080, average year ~2070) under RCP-4.5, 73% of páramo would be vulnerable to tree invasion from the TMCF at lower elevations (Fig 10, S16–S19 Tables). As early as ~2040 (the years 2041–2060, average year ~2050), the portions of páramo affected by reduced humidity are also large, reaching 17 to 50% under the RCP-4.5 and RCP-8.5 scenarios, respectively. Considering both frost frequency reductions and RH declines, with RCP-4.5, ~2070, 70 to 100% of ecoregional páramo, depending on the ecoregion, would be affected. These percentages increase under RCP-8.5 to about 93 to 100% of páramo ecoregions (Fig 10, S16–S19 Tables).

**Fig 11:**
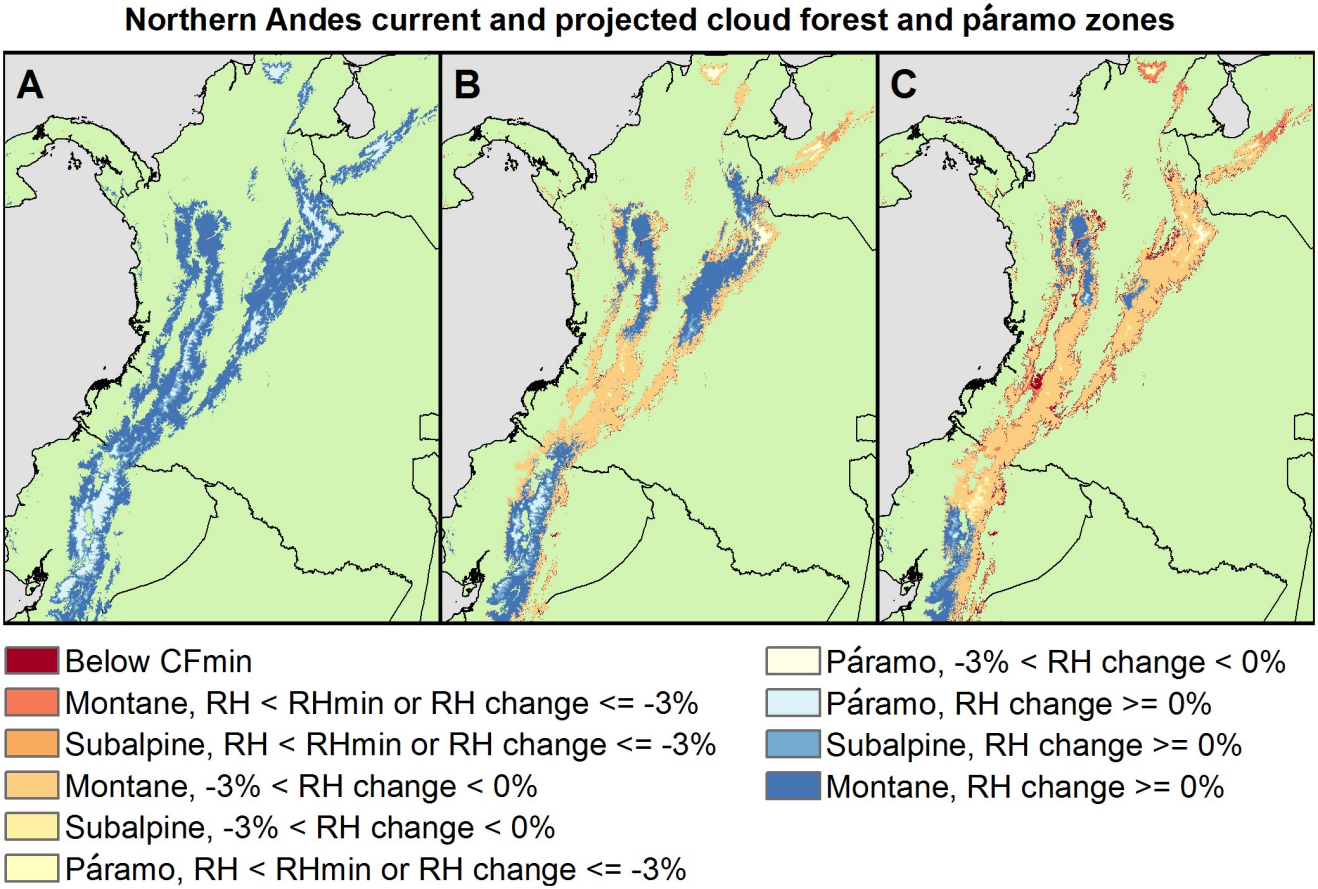
Drying and contraction of TMCF and páramo zones example. Páramo and TMCF zones of the northern Andes for current conditions (A), and for ~2070 under (B) RCP-4.5 and (C) RCP-8.5.

As for the spatial distributions of these RH and frost declines, in general, more páramo dries in the northernmost Andes; warming predominates further south (Fig 11). Subalpine TMCF zones contract at lower elevations. Although both subalpine and montane zones expand into alpine zones (Fig 10), net TMCF zone areas with stable RH decline (S4–S7 Tables). Assuming the frost and RH declines eventually cause páramo invasion by non-páramo species, including trees from lower-elevations, we project that numbers and sizes of contiguous patches of páramo would eventually decline. For example, the number of contiguous areas (of at least one 250-m cell) with páramo climate (currently 1,472) would eventually drop to about 364 under RCP-4.5, and to 208 areas under RCP-8.5 (by ~2070).

### Implications for protection, restoration and monitoring

#### Forest cover and protection in TMCF zones

Neotropics-wide and regionally, TMCF zones are largely forested, but deforestation is apparent at subregional and ecoregional scales. The percent of TMCF zone areas forested is 86% of total Neotropical, 84% of Caribbean, 85% of Mesoamerican, and 86% of South American (Table 1). At the subregional (>1 ecoregion) scale, subregions with more TMCF deforested than elsewhere include Northwest Mesoamerica and the Northern Andes, where forest cover drops to 81% (Table 1). Middle Mesoamerica TMCF is only 83% forested.

**Table 1:** Regional and subregional TMCF zone areas by forest cover and protection^a^.

Subregion or Region^a^	Montane plus Subalpine or Mixed TMCF Zone Area (km2)^b^	Forest Area Within TMCF Zone Area (km2)^b^	Un-protected Non-forest Within TMCF Zone Area (%)	Protected Non-forest Within TMCF Zone Area (%)	Un-protected Forest Within TMCF Zone Area (%)	Protected Forest Within TMCF Zone Area (%)	Forest Within TMCF Zone Area (%)
**Greater Antilles**	3,286	2,748	14	2.8	20	64	84
**Lesser Antilles**^c^	174	161	0.7	7.1	7.5	85	92
**Trinidad and Tobago**	16	16	0	0	88	12	100
**Caribbean**	3,477	2,925	13	2.9	20	64	84
**Middle Mesoamerica**	25,410	21,210	15	1.9	59	24	83
**Northeast Mesoamerica**	10,540	8,996	13	1.6	74	12	86
**Northwest Mesoamerica**	10,900	8,789	15	4.1	68	13	81
**Southern Mesoamerica**	1,574	1,534	1.5	1.1	43	55	98
**Talamancan Mountains**	7,593	7,175	4.5	1	36	59	95
**Mesoamerica**	56,020	47,700	13	2.1	60	25	85
**North Central Andes**	48,570	41,790	13	0.77	58	28	86
**Northern Andes**	151,700	122,100	18	2	63	18	81
**Northern South America**	14,720	14,360	0.92	1.6	31	67	98
**Pacific Coastal**^c^	1,302	784	0.84	39	22	38	60
**Southeast South America**^c^	4,132	3,182	16	6.4	37	40	77
**Southern and South-Central Andes**	121,300	110,200	8.1	1.1	56	34	90
**South America**	341,800	292,400	13	1.6	58	28	86
**Neotropics**	401,300^c^	343,000^c^	13	1.7	58	28	86

^a^S14 and S15 Tables give these data by ecoregion.

^b^At 30-m forest cell size.

^c^Nonforest class includes significant deciduous forest, dry scrub, savanna, or fumarole vegetation, and not all forest absence equates to deforestation.

Deciduous forest, dry scrub, savanna, or fumarole vegetation near TMCF reduces forest cover in some ecoregions and subregions (as indicated in Table 1 and S14 and S15 Tables), even where the TMCF has little deforestation. Instead, the vegetation within a 250-m cell is a natural mosaic, or the TMCF areas are narrow or small, overlapping with the non-TMCF vegetation. Of 12 such ecoregions, half have little (<5%) of their area (whether forested or not) in both the least affected category of change and protected (S8 and S9 Tables). The results below pertain to where much of the nonforest represents deforestation.

At ecoregional scales, Mesoamerican ecoregions with the least forest cover (≤82%, from significant deforestation) included, from most to least deforested, pine-oak or montane forests of the Trans-Mexican Volcanic Belt, Central America, Hispaniola (mainly in Haiti), the Sierra Madre Oriental, and Veracruz (S14 and S15 Tables). South American ecoregions with ≤82% forest cover included four of the seven ecoregions in the Northern Andes, with the least forest cover in the Magdalena Valley, along the western Andean slope (in the Northwest Andes ecoregion), and in the Venezuelan Andes. Other ecoregions with higher (>82%) overall forest cover but some Unprotected forest occupies most (58% and 60%) of current TMCF in South America and Mesoamerica, respectively (Table 1). Protected forest covers an additional 25 and 28% of TMCF in these regions. In contrast in the Caribbean, unprotected forest covers only 20% of TMCF, while 64% is protected forest. Protected nonforest occupies small areas in most ecoregions, usually much smaller than protected forest area.

#### Forest cover and protection where TMCF cloud immersion is least affected

Increasing protection might better conserve TMCF where TMCF cloud immersion is projected to be least affected by climate change. Consequently, these results focus primarily on forest cover and protection where montane TMCF is projected to be least affected under the moderate, RCP 4.5, ~2070 scenario. Under this scenario, the regionally least-affected categories of projected cloud immersion changes were: -3% ≤ RH_d_ < 0% in the Caribbean and Mesoamerican regions and RH_d_ ≥ 0% in South America (Fig 4, S4–S7 Tables).

In more than half of all ecoregions, only a minority of montane TMCF will be in a least affected category of cloud immersion change, and most of these areas have little protection. Thirteen of all 24 ecoregions in the Caribbean and Mesoamerica, and 14 of all 24 ecoregions in South America, have less than about one-third (≤35%) of their montane TMCF area in the regionally least affected change categories (S8 and S9 Tables). Excluding ecoregions where natural nonforest vegetation predominates nonforest areas (leaving 36 ecoregions), 24 of 36, or 67% of ecoregions, have less than five percent of their montane TMCF area categorized as both protected forest and in the regionally least affected change category (50% of all ecoregions).

Some ecoregions have more opportunities for protection than others. In 14 of the above 24 ecoregions (29% of all ecoregions), at least five percent of TMCF is categorized as unprotected forest and in the regionally least affected cloud immersion change category, offering protection opportunities. More extensive protection opportunities occur where much of the montane TMCF that will be least affected by climate change is unprotected forest. For example, some ecoregions have more than one quarter (≥23%) of their montane TMCF in the regionally least affected category of cloud immersion change, while having among the smallest portions of protected forest. In the Caribbean and Mesoamerica, these ecoregions include the pine-oak forests of the Sierras Madre del Sur, de Oaxaca and Occidental, and the Oaxacan montane forests, which have <1% of their area in protected forest in the least affected category, and the Sierra Madre Oriental and Chimalapas, which have <5% of least-affected montane TMCF protected (S8 Table). These latter ecoregions have about 90% or more of their montane TMCF that is projected to be least climate affected in unprotected forest. In South America, ecoregions with the largest proportions (≥24%) of montane TMCF in the least affected category of cloud immersion change and the least (<5%) protected forest include the Northwest Andes, the Magdalena Valley and the Cordillera Oriental North (S9 Table).

Some ecoregions have high deforestation where montane TMCF is in the least affected change category. In some of these places there is little least-affected montane TMCF. Ecoregions with the most deforestation of montane TMCF in the least affected change category in the Caribbean and Mesoamerica include: Hispaniolan montane forests (calculated from S8 Table, *e*.*g*, (0.8% + 0%)/4.8% = 17% of the small portion of least affected area), and pine-oak forests of the Trans-Mexican Volcanic Belt (11%) and Central America (12%). Mixed TMCF in the Trans-Mexican Volcanic Belt, the Sierra Madre Oriental and Central America also have high deforestation in the small areas with the least projected cloud immersion change.

In South America, ecoregions with the most deforestation of montane TMCF in the least affected category of cloud immersion change (outside of the ecoregions mentioned with no least-affected TMCF) are: the Magdalena Valley (41%), the Cordillera Oriental (37%), the Northwestern Andes (34%), the Cordillera Oriental north (27%), the Eastern Cordillera Real (22%), and the Venezuelan Andes (20%) (calculated from S9 Table).

## Discussion and conclusions

### The TMCF mapping algorithm

The mapping algorithm, based on a simple, novel model for CF_min_, plus RH and frost, proved useful for continental-scale mapping of TMCF for current and projected climates. Further, its outputs quantify change in climate parameters widely used to characterize changes in TMCF cloud immersion: CF_min_, which is closely related to typical LCL; and RH. The model for CF_min_ performed well operationally and followed expected relationships. Simple models, like the one here for CF_min_, have advantages over complex ones if they are useful [53]. In part, the usefulness of our algorithm stems from it avoiding the drawbacks of related studies. Past work may cover a smaller area, require vegetation maps for model parameterization, have too coarse of a spatial scale to link climate changes to TMCF, not be able to project climate-induced changes in TMCF zone extent, metrics for cloud immersion, or frost, or, have low sensitivity to insular and coastal TMCF while overestimating TMCF elsewhere.

To expand on the last point above, our maps of current TMCF zones may better represent TMCF distribution. First, they have better overall sensitivity to TMCF (using the most comparable metric available: 95% here, vs. 81% in prior work). Second, and in an example of increased sensitivity, prior continental scale mapping tends to miss coastal and insular TMCF [35], or it assigns to these areas relatively low probability of occurrence [36]. In contrast, the algorithm used here was sensitive to these areas. A drawback of relying on model sensitivity to evaluate mapping algorithms is that sensitivity increases with the area of cloud forest mapped [76]. Third, however, the approach here may better target TMCF (*i*.*e*., have better specificity/low commission error). The small extent of TMCF mapped here, as compared with the more broadly defined “cloud-affected forest” mapped in prior work [35], suggests that the high sensitivity achieved in this study is not attributable to overestimating TMCF zone area. For example, the prior work indicates extensive cloud-affected forest across the Campos Rupestres of Southeast Brazil. Although this area may have frequent overhead cloud cover, it is not clear that much TMCF occurs there. Other prior work [36] maps TMCF occurrence probabilities for this area that are as large as those that it predicted for known TMCF on islands. Our algorithm did not include this area with TMCF (Fig 5). Area estimates are consistent with better targeting of TMCF. The estimate of total Neotropical TMCF zone area here is about one-fifth that of the hydrologically-defined “cloud-affected forest” in [35].

### Cloud immersion and frost changes

We project that about 60–90% of Neotropical TMCF will undergo cloud declines, compared with TMCF expanding by only around 1% from increased cloud immersion and in only a few places. Prior studies suggest that TMCF cloud immersion will increase, decrease, or both, or change only slightly [2, 16, 21, 23, 24, 77]. That work includes dynamic climate modeling at a few TMCF sites, estimation of cloud base heights or LCLs at even fewer sites (with climate, radiosonde, ceilometer, or remote sensing data), and global modeling at spatial scales too coarse to link the analysis cells to the TMCF within them. Here, we project that with a moderate GHG emissions scenario (RCP 4.5), as early as 2040 declines in cloud immersion dominate projected changes across the Neotropics, affecting 57% of their current area (Fig 4, S4 Table). This portion rises to 86% of TMCF area under RCP 8.5 (Fig 4, S7 Table).

Not only are the projected reductions in cloud immersion widespread in the Neotropics, they are likely to be severe in many places (RH_d_ ≤ -3%, RH ≤ RH_min_, or CF_min_ rising above current TMCF). The most severe changes dominate 16 of 24 ecoregions in the Caribbean and Mesoamerica, non-Andean northern South America, the northernmost Andes (Santa Marta, Cerro Pintado), the Central and Northern Andean foothills, the Bolivian Yungas and the northern part of the Southern Andean Yungas. Almost everywhere, CF_min_ rises. It rises above greater portions of TMCF on smaller and drier mountains everywhere, in unprotected areas in the Caribbean and Mesoamerica, and in unprotected areas on smaller mountains in South America (Fig 5). Moreover, the RH declines are, in 58% of ecoregions, steeper than where studies document upward species migrations.

Smaller declines in RH (<0.5% decline), or stable RH, only occur at higher elevations in the Central Andes, the southern parts of the Northern Andes, parts of the Western Andean slope and the southern part of the Southern Andean Yungas. Increases in RH, and CF_min_ lowering, occur almost exclusively in ecoregions with a clear Pacific influence, such as the Galapagos Islands, Pacific coastal TMCF, the southernmost Southern Andean Yungas and the Auracaria forests of Southeastern Brazil.

The above results, that cloud immersion declines predominate, are consistent with studies and theory [2, 16, 78] suggesting that climate change may increase RH over oceans but not over land. Over land, increased evaporative demand may offset any warming-related increases in precipitation or evaporation, causing RH to decline. Our results suggest that this pattern would strongly affect TMCF.

Declines in cloud immersion from RH decline will also affect most páramo. Further, reduced frost will likely enhance tree invasion of páramo. The finding that TMCF starts transitioning to páramo at frost frequencies of 1–1.5 d yr^-1^ highlights frost as an important páramo delimiter at regional scales. Land use and other factors, like microclimate, also can delimit páramo and can slow tree invasion of Andean alpine zones [79]. However, declines in frost frequency and cloud immersion will likely eventually shrink páramo habitat areas and decrease their number. Depending on the scenario and ecoregion, these changes will affect 70–98% of páramo area.

### Implications for protection, restoration and monitoring

Superimposing the projected changes in TMCF cloud immersion onto current forest cover and protection status as in this study can guide climate change response frameworks. Given the high endemism of TMCF, planning at least as fine as at the ecoregion scale is needed, even though TMCF are largely forested at regional scales. Examples below from our results help reveal where forest protection, monitoring, restoration, or community involvement may be most urgently warranted or least costly.

Actions to protect TMCF species are indicated where less severe changes in cloud immersion overlap unprotected forest. Places with the least negative changes in cloud immersion are presumably more likely to be climate refugia than more severely impacted ones, though we recognize that TMCF species vary in their optimal ranges of habitat attributes. Such places include the half or more of ecoregions with less than five percent of their TMCF area projected to be both protected forest and in the regionally least affected category of cloud immersion change under RCP 4.5, ~2070. The majority of these ecoregions (at least ~30% of all ecoregions), have at least five percent of least affected area in unprotected forest that could conceivably be protected.

Protection may be least costly in ecoregions with large portions of unprotected, intact forest in the least affected categories of cloud immersion change, but with little or no protected forest. In Mexico, for example, six ecoregions have extensive (one-fourth or more) unprotected, intact forest in the regionally least affected climate change category (S8 Table; see also [20]) while having <5% of least affected TMCF protected. Similar circumstances apply to three South American ecoregions (S9 Table).

Protection is probably most urgent where the areas projected to be least affected by climate change are both unprotected and a small portion of the TMCF in an ecoregion. Only 2.4% of the Peruvian Yungas foothills, for example, falls into the least-climate-affected category under RCP 4.5, ~2070. Expanding protection and habitat connectivity in those areas could help conserve endemic birds and mammals, as no protected areas cover the habitat of one-third of these species [6].

**Fig 12:**
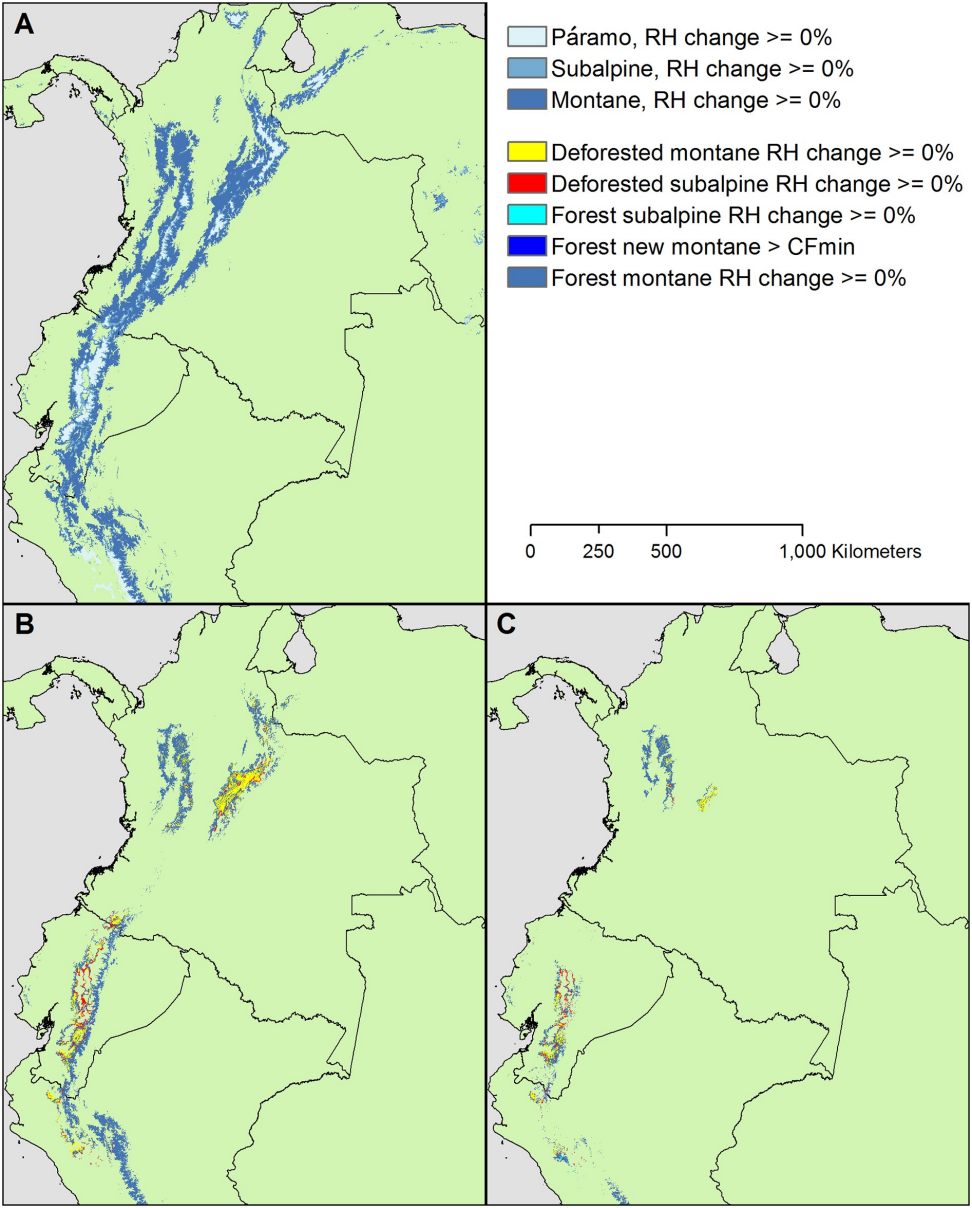
Deforestation is extensive in some places with stable cloud immersion. (A) Páramo and TMCF zones of the north, north-central and northwest Andes for current conditions, (B)-(C) remaining TMCF zones with RH_d_ ≥ 0% with areas currently deforested indicated by color for ~2070 under (B) RCP-4.5 and (C) RCP-8.5.

Both protection of old TMCF remnants and TMCF restoration are urgent where extensive deforestation prominently overlaps with areas least affected by changes in cloud immersion (Fig 12 and S8 and S9 Tables). Ecoregions with populated plateaus or inter-mountain valleys, as occur in parts of Mexico, Central America, the Andes, and Hispaniola, have more TMCF deforestation than elsewhere, with overall TMCF zone forest cover of about 80% or less. However, it is in Andean ecoregions, such as the Magdalena Valley, where extensive deforestation occurs precisely where the RH changes are smallest (Fig 12). Parts of other Northern Andean ecoregions also show this pattern. Further, another similar area stretches from Southern Colombia to Northern Peru and includes parts of the Northwestern Andean, Eastern Cordillera Real and Peruvian Yungas ecoregions, encompassing much of the Cordillera Real from 1°N–7°S and the Western Andean slope from 1°S–7°S.

Protection of remaining TMCF patches in deforested areas is warranted, in part, because we know that the deforested areas surrounding them are most likely to reforest first: proximity to existing forest is often the best predictor of forest regrowth spatial patterns for both socioeconomic and ecological reasons, including in tropical montane forest and other forest zones [80–82]. In addition, secondary forest near existing forest geometrically increases mean forest patch size in these landscapes [80]. Community-driven TMCF restoration has been successful in Andean landscapes [83].

Community involvement is also needed where deforestation or forest degradation threaten protected areas. For example, Monarch butterflies (*Danaus plexippus*) spend their winters, by the millions, in the cloud forests of the Trans-Mexican Volcanic Belt [75]. Most of the montane TMCF in this ecoregion, including in protected areas, will undergo severe declines in cloud immersion. It is at higher elevations in the protected areas there, however, where we project the smallest RH declines. Remote sensing has shown that programs supporting reserves and their surrounding communities there helped to minimize logging and deforestation in protected areas [84].

By mapping cloud forests and páramo and projecting changes in indicators of cloud immersion at relatively fine spatial scales, these results can also guide risk assessments and monitoring. Most species range models do not take mist immersion into account [85]. Our results allow risk assessments to consider cloud immersion and frost and can guide monitoring programs that span a larger range of projected climate change effects. More specifically, these results suggest that increased monitoring of unprotected TMCF may be warranted. For example, we found that unprotected lands across Mesoamerica and the Caribbean, and on smaller mountains in South America, will likely undergo larger declines in cloud immersion than protected lands (S14 and S15 Tables). This pattern emerges because: 1) protected lands are at higher elevations in these areas, and 2) a larger part of TMCF is affected by a given increase in CF_min_ on smaller-mountain ecoregions. Monitoring only major protected areas will underestimate species’ vulnerability to climate change.

In Puerto Rico, for instance, the threatened Elfin-Woods Warbler (*Setophaga angelae*) is restricted to higher elevation forests, including its namesake “elfin” ridgetop cloud forests (Fig 1) and the cloud forests below the elfin forests [86, 87]. Changes in cloud cover are a risk factor for the species [88, 89], though this species may be adaptable to some change [87]. One of the warbler’s two known populations occurs where we project the steepest cloud immersion declines, in western Puerto Rico, and extends into unprotected lands. The other population occurs in the wettest TMCF on the island, in Northeastern Puerto Rico, which is intensively monitored protected land. Climate or other monitoring focused only on this major protected area could underestimate climate change effects on the species as a whole.

### Climate change *vs*. current TMCF land use: Concluding remarks

We projected that páramo and TMCF habitat will shrink. Cloud immersion declines for the majority of Neotropical ecoregions will be similar to or more severe than the changes where upward species migrations or rising cloud base heights are documented. The result suggests widespread TMCF drying and upward shifts of TMCF minimum elevations and species. Both of these changes imply widespread TMCF habitat shrinkage. Mountain surface area declines with increasing elevation, and high-elevation plateaus are often too dry for TMCF specialists. Our results further suggest that declines in TMCF area will not be offset by warming in humid alpine areas. Further, páramo habitat patches, too, will likely shrink, dry, or disappear. With many TMCF and páramo species being range-restricted, the principal that species richness declines with habitat area applies [85]. We conclude that shrinking habitat areas due to climate change alone will likely cause local species extirpations, if not widespread extinction of some TMCF and páramo species.

Land use will likely exacerbate climate change effects in some places. In Peru, tree communities changed with gradual warming since the last ice age, but current warming is ten times faster [73]. Slow upward tree species migration, combined with difficulties colonizing alpine areas that are exacerbated by land use, adds to species vulnerability [73, 79]. In Costa Rica, lowland deforestation pushes cloud base heights upwards [18]. Furthermore, in South America, four to 19% of Andean ecoregions that are least climate-affected are already deforested (S9 Table), reducing options for climate change adaptation.

For TMCF across much of the Neotropics, and for most páramo areas, climate change poses a risk to species over areas at least as extensive as the areas that may be negatively affected by land use, regardless of future land cover. Globally, more species are vulnerable to climate change than to land-use change [90]. Here we projected that 100% of the area of TMCF or páramo zones in many Neotropical ecoregions will undergo substantial cloud immersion or frost declines. A sixth mass extinction has been underway since the industrial age began [91]. Neotropical TMCF and páramo species losses will likely add to it. This study can help guide potential conservation efforts to stem the loss of TMCF and páramo species relative to what they might otherwise be.

## Supporting information

S1 Review Review of methods to map cloud forests or project their climates. (DOCX)

S1 Fig Observed vs. predicted RH. Observed annual hourly relative humidity (RH) plotted as a function of RH as predicted by the mapping model log (RH/1-RH) = 4.32E-04 + 0.0162Bio1** + 0.178Bio2*** − 0.0144Bio7*** + 0.00100Bio12*** − 0.0107Bio2*Bio2*** − 3.02E-05Bio1*Bio12*** (N = 391; Parameter estimate Pr > F: <0.0001 = ***, <0.01 = **).
(TIF)

S2 Fig Observed vs. predicted frost where frost ≥ 10 d·yr^-1^. Annual number of days with temperatures ≤ 0°C (Frost) plotted as a function of Frost predicted by the mapping model log(Frost/1-Frost) = -2.52*** + 0.122Bio1** − 0.511Bio6*** − 0.0176Bio11 − 0.00870Bio6*Bio11* − 0.0128Bio1*Bio11** + 0.0287Bio1*Bio6*** (N = 220 stations with Frost > 0; Parameter estimate Pr > F: <0.0001 = ***, <0.01 = **, <0.1 = *). If this model predicted < 10 days of Frost per year, the results from the model in S3 Fig were substituted for the results from this model, because this model overestimated Frost at those low values.
(TIF)

S3 Fig Observed vs. predicted frost where 0 < frost < 10 d·yr^-1^. Annual number of days with temperatures ≤ 0°C (Frost) plotted as a function of Frost predicted by the mapping model *log(Frost d yr*^*-1*^ *) = 2*.24* + 0.280Bio1*** − 0.186Bio6* − 0.429Bio11*** (N = 84, Pr > F <0.0001, Parameter estimate Pr > F: <0.0001 = ***, <0.01 = **, <0.1 = *).
(TIF)

S4 Fig The model for CF_min_ followed expected relationships. Relationships between cloud forest minimum elevation (CF_min_) and (A) maximum watershed elevation (ELEV_max_) and (B) relative humidity from 100–150 m elevation (RH_150_). More specifically, (A) observed (circles) and predicted log(CF_min_) (thick black line) plotted against ELEV_max_ *for mean RH*_*150*_ showing 95% confidence bands for log(CF_min_) (shaded bands), and (B) observed (circles) and predicted log(CF_min_) (thick black line) plotted against RH_150_ for *mean Elev*_*max*_ showing 95% confidence bands for log(CF_min_) (shaded bands).
(TIF)

S1 Table Data, spatial resolutions, metrics, and methods of studies that map or project TMCF conditions. (DOCX)

S2 Table Variable definitions. (DOCX)

S3 Table Cloud forest minimum elevations used to parametrize CF_min_ model. (DOCX)

S4 Table Neotropical and regional cloud immersion changes under RCP 4.5, 2041–2060. (DOCX)

S5 Table Neotropical and regional cloud immersion changes under RCP 4.5, 2061–2080. (DOCX)

S6 Table Neotropical and regional cloud immersion changes under RCP 8.5, 2041–2060. (DOCX)

S7 Table Neotropical and regional cloud immersion changes under RCP 8.5, 2061–2080. (DOCX)

S8 Table Changes in montane TMCF cloud immersion by ecoregion, and forest cover and protection of the least affected change category, Mesoamerica and the Caribbean. (DOCX)

S9 Table Changes in montane TMCF cloud immersion by ecoregion, and forest cover and protection of the least affected change category, South American ecoregions. (DOCX)

S10 Table Regional cloud immersion changes by protection status for RCP 4.5, 2041–2060. (DOCX)

S11 Table Regional cloud immersion changes by protection status for RCP 4.5, 2061–2080. (DOCX)

S12 Table Regional cloud immersion changes by protection status for RCP 8.5, 2041–2060. (DOCX)

S13 Table Regional cloud immersion changes by protection status for RCP 8.5, 2061–2080. (DOCX)

S14 Table Forest cover and protection by ecoregion, Caribbean and Mesoamerica. (DOCX)

S15 Table Forest cover and protection by ecoregion, South America. (DOCX)

S16 Table Changes in páramo cloud immersion and frost for RCP 4.5, years 2041–2060. (DOCX)

S17 Table Changes in páramo cloud immersion and frost for RCP 4.5, years 2061–2080. (DOCX)

S18 Table Changes in páramo cloud immersion and frost for RCP 8.5, years 2041–2060. (DOCX)

S19 Table Changes in páramo cloud immersion and frost for RCP 8.5, years 2061–2080. (DOCX)
