# Supplementary material for: Neotropical cloud forests and páramo to contract and dry from declines in cloud immersion and frost
Source: PLoS One. 2019 Apr 17;14(4):e0213155. doi: 10.1371/journal.pone.0213155 (PMC6469753; doi:10.1371/journal.pone.0213155)
Supplement: S1 Review — (DOCX) [file pone.0213155.s001.docx]

**S1 Review**

**Review of methods to map cloud forests or project their climates**

We reviewed studies that map cloud forest or páramo climatic zones, or that project changes in their extent or climate (S1 Table) and categorized them into five types according to their methodology. These five types use:

1. Global climate models (GCMs) to project changes in cloud-related climate parameters;
2. Regional climate models (RCMs) to project changes in cloud-related climate parameters;
3. Thresholds in elevation or cloud-related climate parameters to map present or projected cloud forest distributions;
4. Cloud immersion frequency (CIF) estimated from remotely-sensed cloud cover or cloud base heights; or,
5. Empirical models that map current or projected probabilities of climatic zone occurrence from climate, topography or cloud cover. Discrete outputs are based on relative probabilities of occurrence.

All of the studies, including the climate models where used, employ diverse input data derived from a range of sources having wide-ranging horizontal, vertical and temporal resolutions (S1-S2 Tables). They gauge cloudiness with the following cloud-related climate parameters, from most to least common: relative humidity (RH), cloud immersion frequency (CIF), cloud forest minimum elevation (CF_min_), cloud lifting condensation level (LCL), or other variables. Studies using empirical approaches characterize model fits. Some studies projecting change estimate ranges or standard deviations of different model projections. Some studies with discrete mapped outputs estimate classification errors. None of the studies propagate error.

Drawbacks of these methods vary. Methods using only GCMs have resolutions too coarse to know if the estimated climate changes apply to TMCF and too coarse for management (S1 Table). Those using RCMs have regional or smaller spatial extents. Methods relying on elevation or remotely sensed cloud cover to gauge cloudiness cannot predict cloud forest conditions under projected climate change. Methods using cloud forest maps to parameterize empirical models are limited to where vegetation maps distinguish cloud forest from other forests, which most do not [1]. In Tovar et al. [2], for example, cloud forest and montane evergreen forest are the same class in the reference maps used. Consequently, they are the same class in output maps. Only three studies explicitly consider cloud forest upper limits or páramo, including this one (S1 Table). Finally, past studies do not explicitly account for the effects of mountain size on cloud formation.

Several studies have the disadvantage of parameterizing thresholds or empirical models with the WCMC locations. The problem with these points is that they represent centers of known cloud forest sites and so represent either maximum or middle elevations [3, 4]. Adding to this imprecision is that the WCMC points are known to have spatial error [4-6]. Most of them have a maximum locational precision of 1-minute latitude and longitude, which is about 2 km at the equator; and, many have only 0.25 to 1-degree precision (28 to 111 km at the equator).

**Achieving resolutions fine enough to map cloud forests**

All of the spatially explicit studies in S1 Table that map cloud forest zones with ~1 km resolutions use Worldclim climate maps [7], which owe their ~1-km resolution to a ~1-km digital elevation model (DEM). This resolution is the finest resolution of these studies’ spatial inputs (excepting two using tree cover with ~500-m cells). Mapping climate at high resolution with a DEM is justifiable because topography effectively downscales climate in mountainous terrain [8-10]. Wall-to-wall elevation data are available from remote sensing data at much finer resolution than that of interpolated climate data. In addition, well-known correlations exist between climate and landscape factors like elevation, coastal proximity and facet relative to prevailing winds. Consequently, more accurate and higher-resolution climate maps, in Puerto Rico, for example [9], are possible by assigning a climate value to a cell based not only on the inverse of its distances from surrounding climate stations, but also based on its topography. These strong climate-elevation relationships are why GCMs are too spatially coarse for cloud forest or páramo mapping – each cell covers too great of a range in elevation and related climate. In other words, because mountains are where topography most strongly influences climate, accounting for topography is especially important there. Unfortunately, climate stations are often sparsely distributed in mountains. To bridge this gap, elevation from a ~1-km DEM was incorporated into the Worldclim mapping algorithm.

**Reference data from expert observation**

All of the approaches in S1 Table that rely on thresholds or empirical models required reference data from experts. Experts used field observations, including quantitative or qualitative vegetation observations, image interpretation, maps, or a combination of these sources. The reference data types and the studies using them include: 1) maps of cloud forest or páramo that are based on field work and remote sensing data ([2, 11, 12], this study); 2) geographic coordinates of the centers of known cloud forest areas from the WCMC data, where coordinates were identified with available information from experts, or expert communication [4, 6, 11, 13]; and 3) published CF_min_ values based on field studies (this study). We consistently used the lowest-elevation cloud forest present in a place to designate CF_min_ (most commonly lower montane cloud forest). However, the TMCF in this study also include higher-elevation TMCF (elfin, mixed, montane, upper montane, subalpine). For TMCF nomenclature, we refer readers to existing reviews of cloud forest types and their definitions [13-16].

## References

1. Bruijnzeel LA, Mulligan M, Scatena FN. Hydrometeorology of tropical montane cloud forests: emerging patterns. Hydrological Processes. 2011;25(3):465-98. doi: 10.1002/hyp.7974.

2. Tovar C, Arnillas CA, Cuesta F, Buytaert W. Diverging responses of tropical Andean biomes under future climate conditions. PloS one. 2013;8(5):e63634.

3. Karmalkar AV, Bradley RS, Diaz HF. Climate change scenario for Costa Rican montane forests. Geophysical Research Letters. 2008;35(11):n/a-n/a. doi: 10.1029/2008gl033940.

4. Wilson AM, Jetz W. Remotely Sensed High-Resolution Global Cloud Dynamics for Predicting Ecosystem and Biodiversity Distributions. PLoS Biol. 2016;14(3):e1002415.

5. Jarvis A, Mulligan M. The climate of cloud forests. Hydrological Processes. 2011;25(3):327-43. doi: 10.1002/hyp.7847.

6. Mulligan M. Modeling the tropics-wide extent and distribution of cloud forest and cloud forest loss, with implications for conservation priority. In: Bruijnzeel LA, Scatena F, Hamilton L, editors. Tropical montane cloud forests: science for conservation and management. Cambridge, UK: Cambridge University Press; 2010. p. 14-38.

7. Hijmans R, Cameron S, Parra J, Jones P, Jarvis A. Very high resolution interpolated climate surfaces for global land areas. Int J Climatol. 2005;25(15):1965-78. doi: 10.1002/joc.1276.

8. Daly C, Halbleib M, Smith JI, Gibson WP, Doggett MK, Taylor GH, et al. Physiographically sensitive mapping of climatological temperature and precipitation across the conterminous United States. International journal of climatology. 2008;28(15):2031-64.

9. Daly C, Helmer EH, Quiñones M. Mapping the climate of Puerto Rico, Vieques and Culebra. International journal of climatology. 2003;23(11):1359-81.

10. Daly C, Neilson RP, Phillips DL. A Statistical-Topographic Model for Mapping Climatological Precipitation over Mountainous Terrain. Journal of Applied Meteorology. 1994;33(2):140-58. doi: 10.1175/1520-0450(1994)033<0140:astmfm>2.0.co;2.

11. Nair US, Asefi S, Welch RM, Ray DK, Lawton RO, Manoharan VS, et al. Biogeography of Tropical Montane Cloud Forests. Part II: Mapping of Orographic Cloud Immersion. Journal of Applied Meteorology and Climatology. 2008;47(8):2183-97. doi: 10.1175/2007jamc1819.1.

12. Ponce-Reyes R, Reynoso-Rosales V-H, Watson JEM, VanDerWal J, Fuller RA, Pressey RL, et al. Vulnerability of cloud forest reserves in Mexico to climate change. Nature Clim Change. 2012;2(6):448-52. doi: 10.1038/NCLIMATE1453.

13. Scatena F, Bruijnzeel LA, Bubb P, Das S. Setting the stage. In: Bruijnzeel LA, Scatena F, Hamilton L, editors. Tropical montane cloud forests: science for conservation and management. Cambridge, UK: Cambridge University Press; 2010. p. 3-11.

14. Grubb PJ. Control of Forest Growth and Distribution on Wet Tropical Mountains: with Special Reference to Mineral Nutrition. Annual Review of Ecology and Systematics. 1977;8(1):83-107. doi: 10.1146/annurev.es.08.110177.000503.

15. Stadtmüller T. Cloud forests in the humid tropics: a bibliographic review: Bib. Orton IICA/CATIE; 1987.

16. Foster P. The potential negative impacts of global climate change on tropical montane cloud forests. Earth-Science Reviews. 2001;55(1–2):73-106. doi: 10.1016/S0012-8252(01)00056-3.

S1 Review of Supporting material for:

E. H. Helmer, E. A. Gerson, L. Scott Baggett, Benjamin J. Bird, Thomas S. Ruzycki, Shannon M. Voggesser. 2019. Neotropical cloud forests and páramo to contract and dry from declines in cloud immersion and frost. 2019. PLOS ONE.

Raster data available at: <https://doi.org/10.2737/RDS-2019-0008>
