# Supplementary figures and images for: Neotropical cloud forests and páramo to contract and dry from declines in cloud immersion and frost
Source: PLoS One. 2019 Apr 17;14(4):e0213155. doi: 10.1371/journal.pone.0213155 (PMC6469753; doi:10.1371/journal.pone.0213155)

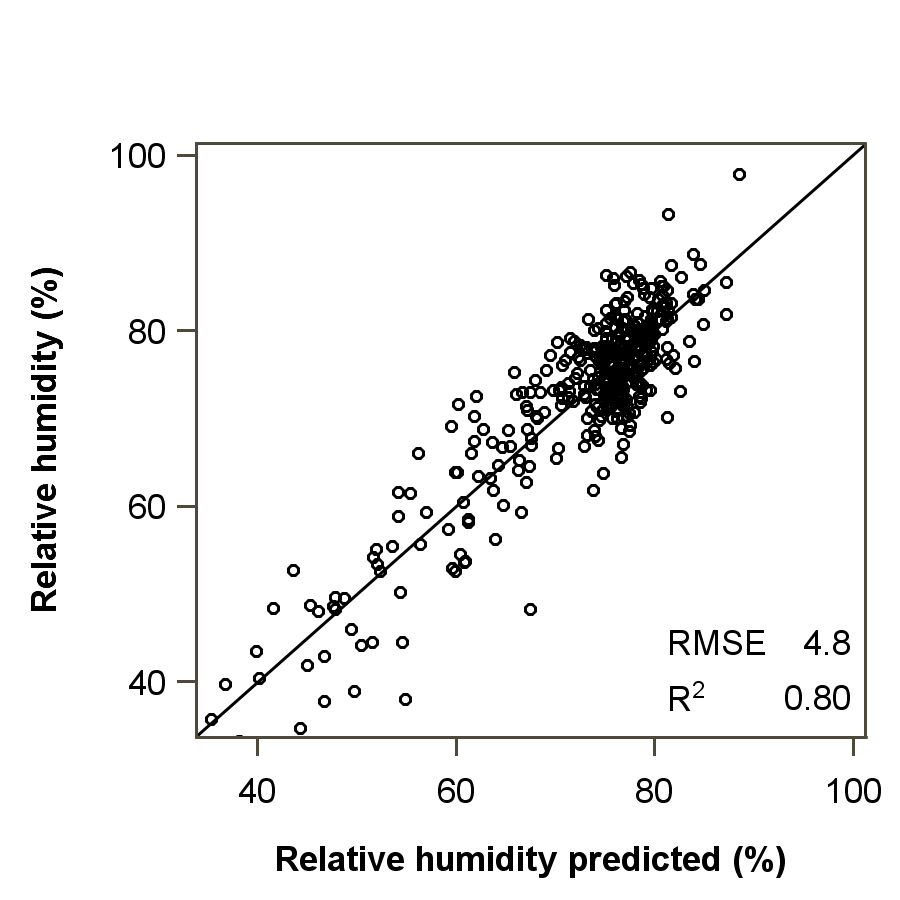

Supplement: S1 Fig — Observed annual hourly relative humidity (RH) plotted as a function of RH as predicted by the mapping model log (RH/1-RH) = 4.32E-04 + 0.0162Bio1** + 0.178Bio2*** − 0.0144Bio7*** + 0.00100Bio12*** − 0.0107Bio2*Bio2*** − 3.02E-05Bio1*Bio12*** (N = 391; Parameter estimate Pr > F: <0.0001 = ***, <0.01 = **). (TIF) [file pone.0213155.s002.tif]

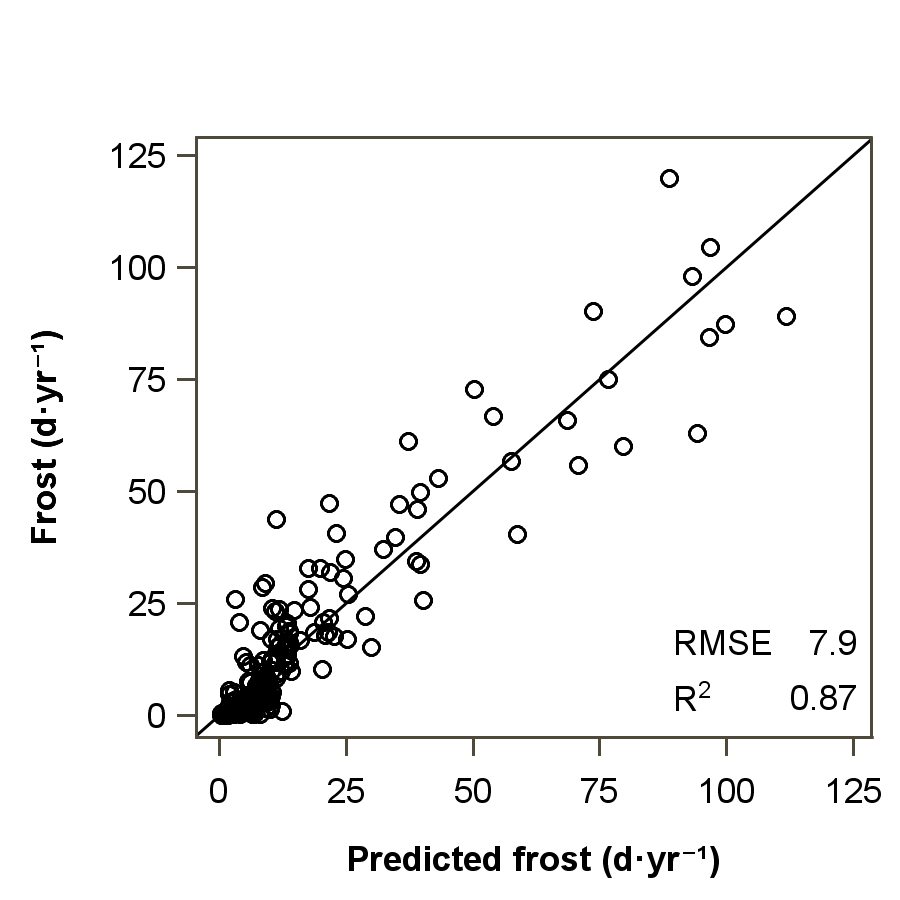

Supplement: S2 Fig — Annual number of days with temperatures ≤ 0°C (Frost) plotted as a function of Frost predicted by the mapping model log(Frost/1-Frost) = -2.52*** + 0.122Bio1** − 0.511Bio6*** − 0.0176Bio11 − 0.00870Bio6*Bio11* − 0.0128Bio1*Bio11** + 0.0287Bio1*Bio6*** (N = 220 stations with Frost > 0; Parameter estimate Pr > F: <0.0001 = ***, <0.01 = **, <0.1 = *). If this model predicted < 10 days of Frost per year, the results from the model in S3 Fig were substituted for the results from this model, because this model overestimated Frost at those low values. (TIF) [file pone.0213155.s003.tif]

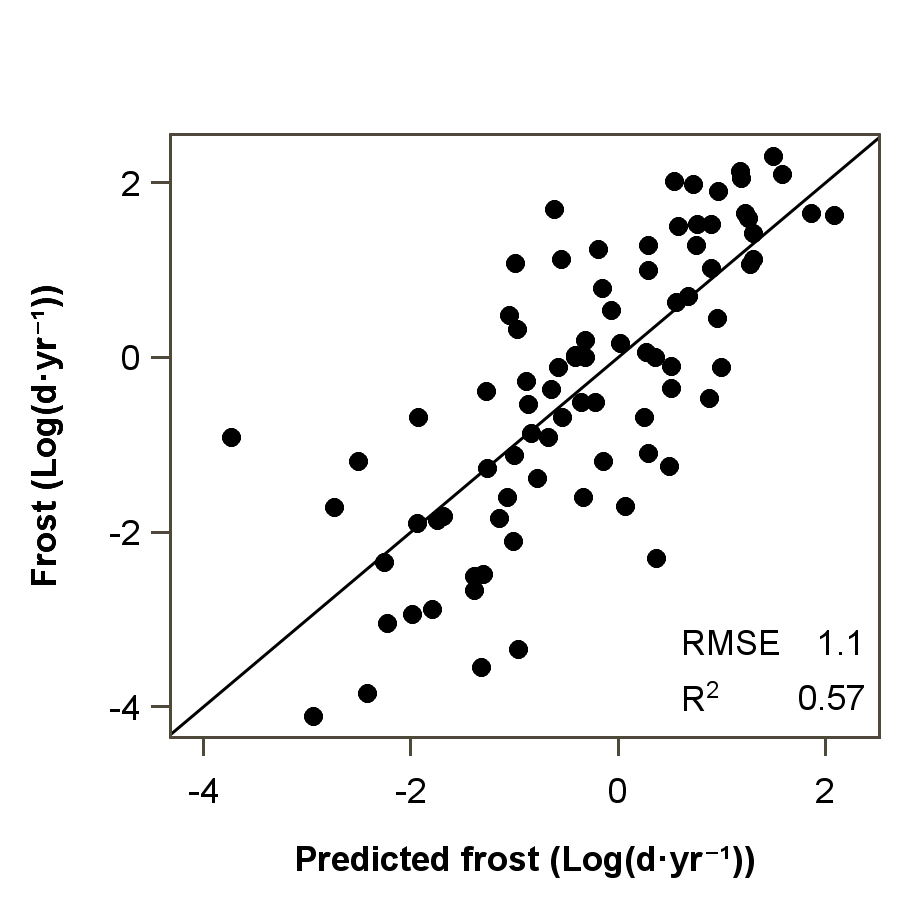

Supplement: S3 Fig — Annual number of days with temperatures ≤ 0°C (Frost) plotted as a function of Frost predicted by the mapping model log(Frost d yr-1 ) = 2.24* + 0.280Bio1*** − 0.186Bio6* − 0.429Bio11*** (N = 84, Pr > F <0.0001, Parameter estimate Pr > F: <0.0001 = ***, <0.01 = **, <0.1 = *). (TIF) [file pone.0213155.s004.tif]

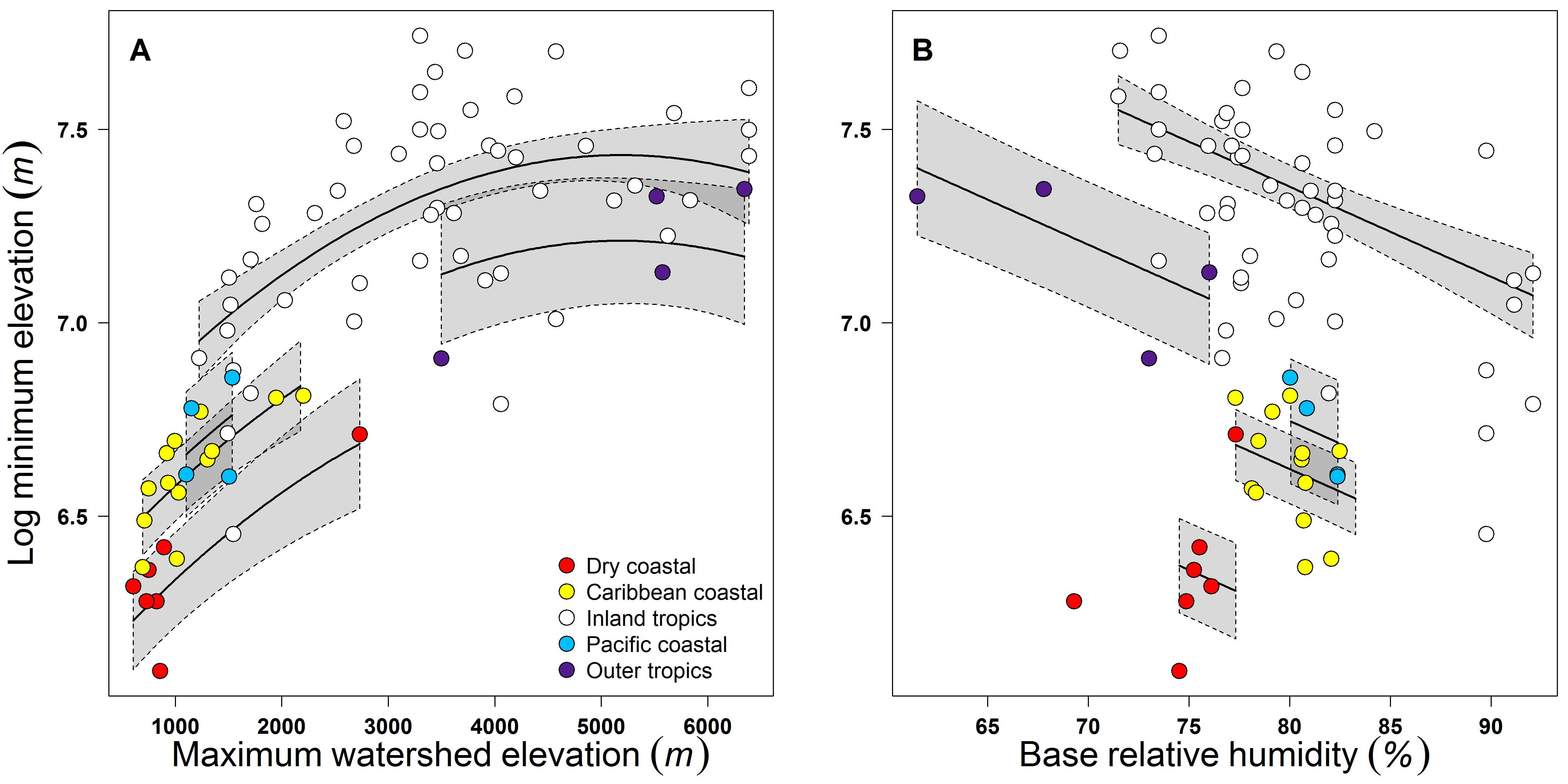

Supplement: S4 Fig — Relationships between cloud forest minimum elevation (CFmin) and (A) maximum watershed elevation (ELEVmax) and (B) relative humidity from 100–150 m elevation (RH150). More specifically, (A) observed (circles) and predicted log(CFmin) (thick black line) plotted against ELEVmax for mean RH150 showing 95% confidence bands for log(CFmin) (shaded bands), and (B) observed (circles) and predicted log(CFmin) (thick black line) plotted against RH150 for mean Elevmax showing 95% confidence bands for log(CFmin) (shaded bands). (TIF) [file pone.0213155.s005.tif]
