# Supplementary material for: Neotropical cloud forests and páramo to contract and dry from declines in cloud immersion and frost
Source: PLoS One. 2019 Apr 17;14(4):e0213155. doi: 10.1371/journal.pone.0213155 (PMC6469753; doi:10.1371/journal.pone.0213155)
Supplement: S1 Table — (DOCX) [file pone.0213155.s006.docx]

S1 Table. Data, spatial resolutions, metrics, and methods of studies that map or project TMCF conditions.

| **Reference** | **Type** | **Method** | **Cloud Forest Location Data** | **Inputs and cell sizes** | **Cloud immersion metric** | **Output Type** | **Output Extent and Cell Size** | **Error Measures** | **Error Propa-gation** | **Projects Change with Climate** | **≤1 km Maps** | **No Strong Bias** | **TMCF- Specific** | **TMCF Upper Limits Defined** | **Cloud Immersion Metrics Mapped** |
| --- | --- | --- | --- | --- | --- | --- | --- | --- | --- | --- | --- | --- | --- | --- | --- |
| Still et al. 1999 | 1 | GCM | 4 sites | GCM (3.75°) | RH, Absolute Humidity, Warmth Index | Continuous (Temperature, Absolute Humidity, Warmth Index, CFMid) | 4 sites | . | None | ✓ |  | ✓ | ✓ |  |  |
| Foster 2001 | 1 | GCM | . | Four GCMs (>5°) | RH | Continuous  (RH) | Global (>5°) | Std. Dev. of RH | None | ✓ |  |  |  |  | ✓ |
| Sperling et al. 2004 | 1 | GCM | 1 site  4 locations | Three GCMs (2.8° to 5.6°)  Daily Climate Readings | RH | Continuous  (Temperature, Specific Humidity, RH, CFMid) | 1 site, 4 locations | Change significance (*p-values)* | None | ✓ |  | ✓ | ✓ | ✓ |  |
| Pounds et al. 2006 | 2 | RCM | 1 site | Temperature (5°)  Sea Surface Temperature (2°)  RCM (1.6 to 25 km), Elevation (1 km) | RH | Continuous (RH) | Regional  (1.6 km) | . | None | ✓ |  | ✓ | ✓ |  |  |
| Nair et al. 2008 A | 4 | CIF | Map  WCMC sites | Cloud Top Height (1°)  Cloud Thickness (5 km)  Cloud Cover (5 km), Elevation (1 km) | CIF | Continuous (CIF) | Regional  (1 km) | Goodness of Fit | None |  | ✓ | ✓ | ✓ |  | ✓ |
| Nair et al. 2008 B | 2, 4 | CIF | Map  WCMC sites | RCM (1 to 4 km), Cloud Cover (1 km) | CIF | Continuous (CIF) | Regional  (1 km) | . | None |  | ✓ |  | ✓ |  | ✓ |
| Karmalkar et al. 2008 | 2 | RCM | RCM | Climate (0.5°), RCM (25 km) | LCL | Continuous (LCL, Temperature, Precipitation) | Regional  (25 km) | Std. Dev. of Temperature Precipitation | None | ✓ |  |  | ✓ |  | ✓ |
| Scatena et al. 2010 | 3 | Thresholds in CFMin | CFMin  WCMC sites | Six Regions, Elevation (1km)  Forest Cover (1 km),  Forest Cover (500 m) | None | Discrete | Pan-Tropical (1 km) | . | None |  | ✓ |  | ✓ | ✓ |  |
| Mulligan 2010 | 3, 4 | Thresholds in RH, CIF | WCMC sites | Sea Level Pressure (5°)  Temperature (1°),  Relative Humidity (1°),  Cloud Cover (1°)  Elevation (1km), Forest Cover (500 m) | RH, CIF | Discrete | Pan-Tropical (1 km) | Sensitivity | None |  | ✓ |  | ± |  | ✓ |
| Foster 2010 | 1 | GCM | . | GCM (2.8°) | LCL | Continuous (LCL) | Global (≥2.8°) | Std. Dev. of LCL | None | ✓ |  |  |  |  | ✓ |
| Rojas-Soto et al. 2012 | 5 | Cloud Forest Probability | Tree Species Maps | GCMs (various)  Climate (1 km), Elevation (1 km) | None | Discrete | Regional  (1 km) | Class Error | None | ✓ | ✓ | ✓ | ✓ |  |  |
| Tovar et al. 2013 | 5 | Montane. Forest Probability | Map | Climate (1 km), Slope (1 km)  Topographic Contour Index (1 km) Topographic Ruggedness Index (1 km) | None | Discrete | Regional  (1 km) | Class Error | None | ✓ | ✓ | ✓ |  | ✓ |  |
| Ponce-Reyes et al. 2014 | 5 | Cloud Forest Probability | Map | Climate (1 km), Forest (1 km) | None | Discrete | Regional  (1 km) | Goodness of Fit | None | ✓ | ✓ | ✓ | ✓ |  |  |
| Wilson, Jetz 2016 A | 4, 5 | Cloud Forest Probability | WCMC sites | Two Regions  Cloud Cover (1 km), Elevation (1 km) | None | Probability | Global  (1 km) | Goodness of Fit | None |  | ✓ |  | ✓ |  |  |
| Wilson, Jetz 2016 B | 5 | Cloud Forest Probability | WCMC sites | Two Regions  Climate (1 km), Elevation (1 km) | None | Probability | Global  (1 km) | Goodness of Fit | None |  |  |  | ✓ |  |  |
| This study | 3, 5 | Thresholds in CFMin, RH, Frost | CFMin | Five Regions, Climate (1 km)  Watersheds (1 km), Watersheds (30 m), Elevation (250m), Forest Cover (30 m), Hourly Climate Readings | CFMin, RH | Discrete, Continuous, CFMin, RH, Frost | Western Hemisphere Tropics  (250 m) | Goodness of Fit, Sensitivity | None | ✓ | ✓ | ✓ | ✓ | ✓ | ✓ |

**CFMid**=Middle cloud forest elevation (m), **CFMin**=Minimum cloud forest elevation (m), **CIF**=Cloud Immersion Frequency (%), **GCM**=Global Climate Model, **LCL**=Lifting Condensation Level (m), **RCM**=Regional Climate Model, **RH**= Relative Humidity (%), **WCMC**=World Conservation Monitoring Centre, **Frost**=Days per year with hourly temperatures ≤ 0° C, **±**=Hydrologically defined.
