# Supplementary material for: Neotropical cloud forests and páramo to contract and dry from declines in cloud immersion and frost
Source: PLoS One. 2019 Apr 17;14(4):e0213155. doi: 10.1371/journal.pone.0213155 (PMC6469753; doi:10.1371/journal.pone.0213155)
Supplement: S2 Table — (DOCX) [file pone.0213155.s007.docx]

S3 Table. Variable definitions.

**S2 Table. Variable definitions.**

|  | **Variable Description^a^** |  |
| --- | --- | --- |
| Bio1 | Mean annual temperature (°C) from Worldclim |  |
| Bio2 | Mean annual diurnal temperature range (°C) from Worldclim |  |
| Bio6 | Minimum temperature of the warmest month (°C) from Worldclim |  |
| Bio7 | Temperature annual range (°C) from Worldclim |  |
| Bio9 | Mean temperature of the driest three-month period (°C) from Worldclim |  |
| Bio11 | Mean temperature of the coldest three-month period (°C) from Worldclim |  |
| Bio12 | Total annual precipitation (mm) from Worldclim |  |
| CF_min_ | Minimum cloud forest elevation (m) |  |
| ELEV_max_ | Maximum watershed elevation (km) |  |
| Frost | Average number of days per year with hourly temperatures ≤ 0° (d·yr^-1^) |  |
| Frostmin1 | | Minimum frost frequency (d·yr^-1^) defining lower boundary for subalpine or mixed TMCF (transitional to alpine or other higher elevation vegetation |
| Frostmin2 | Minimum frost frequency (d·yr^-1^) defining lower boundary for alpine or other higher elevation vegetation, including páramo |  |
| LCL | Lifting Condensation Level (m) |  |
| Region, R | Modeling Region |  |
| RH | Average annual hourly Relative Humidity (%) |  |
| RH_150_ | Base Relative Humidity (RH), i.e. average RH from 100 to 150m elevation (%) |  |
| RH_d_ | Current RH minus RH in a future scenario (%) |  |
| RH_min_ | Subregional minimum cloud forest RH |  |
