# Supplementary material for: Neotropical cloud forests and páramo to contract and dry from declines in cloud immersion and frost
Source: PLoS One. 2019 Apr 17;14(4):e0213155. doi: 10.1371/journal.pone.0213155 (PMC6469753; doi:10.1371/journal.pone.0213155)
Supplement: S3 Table — (DOCX) [file pone.0213155.s008.docx]

S3 Table. Estimates of cloud forest minimum elevations.

| **Source** | **Location, Aspect, Region** | | **CF_min_ (m)** | **Longitude (dd)** | **Latitude (dd)** | **CF_min_ Cell (m)** | **ELEV_max_ (km)** | **RH_150_ (%)** | **RH (%)** | |  |
| --- | --- | --- | --- | --- | --- | --- | --- | --- | --- | --- | --- |
| [1] | Sierra Madre Oriental, Tamaulipas, w, OT | | 1,000 | -99.1832 | 23.1162 | 1,000 | 3.499 | 73.0 | 69.7 | |  |
| [2] | Sierra Madre Oriental, Circ.Xalapa, w, OT | | 1,200 | -96.9088 | 19.4833 | 1,249 | 5.576 | 76.0 | 81.9 | |  |
| [3] | Sierra Mazateca, w, IT | | 2,200 | -96.9571 | 18.1403 | 2,211 | 4.575 | 79.3 | 76.6 | |  |
| [4] | Sierra Madre del Sur, St Tomás, w, IT | | 2,200 | -95.9721 | 16.2543 | 2,216 | 3.72 | 71.6 | 81.0 | |  |
| [5] | Sierra Madre del Sur, Sierra Juarez, w, IT | | 1,100 | -96.5194 | 17.7206 | 1,107 | 4.575 | 79.3 | 83.1 | |  |
| [6] | Sierra de Manantlán, l, IT | | 1,950 | -103.979 | 19.5002 | 1,968 | 4.184 | 71.5 | 73.8 | |  |
| [7, 8] | Sierra Madre de Chiapas, w, IT | | 1,667 | -92.7603 | 15.4958 | 1,682 | 4.198 | 77.4 | 77.9 | |  |
| [9] | Sierra de las Minas, Cerro Raxon, w, IT | | 1,300 | -89.7375 | 15.1958 | 1,287 | 3.299 | 73.5 | 87.7 | |  |
| [9, 10] | Sierra de las Minas, Cerro Raxon, l, IT | | 1,825 | -89.8476 | 15.1958 | 1,808 | 3.299 | 73.5 | 90.2 | |  |
| [11] | Montaña de Miranda, w, IT | | 2,000 | -89.8981 | 15.1170 | 1,991 | 3.299 | 73.5 | 89.5 | |  |
| [11] | Montaña de Miranda, l, IT | | 2,300 | -89.9404 | 15.1244 | 2,302 | 3.299 | 73.5 | 90.7 | |  |
| [12, 13] | La Tigra National Park, w, IT | | 1,425 | -87.0897 | 14.2267 | 1,456 | 2.31 | 75.9 | 80.3 | |  |
| [14, 15] | Cordillera de Tilarán, l, IT | | 1,480 | -84.8047 | 10.3102 | 1,476 | 3.458 | 80.6 | 89.5 | |  |
| [16] | Cordillera Central, w, IT | | 1,450 | -84.0900 | 10.2390 | 1,449 | 3.4 | 81.3 | 90.7 | |  |
| [17, 18] | Cordillera de Talamanca, Costa Rica, l, IT | | 1,650 | -83.8368 | 9.5329 | 1,656 | 3.458 | 80.6 | 96.2 | |  |
| [19] | Serrania de Sapo, Cerro Sapo, wl, PC | | 700 | -78.3583 | 7.9811 | 741 | 1.102 | 82.4 | 85.5 | |  |
| [19] | Cordillera de Juardó ridge, wl, PC | | 730 | -78.0484 | 7.6822 | 737 | 1.504 | 82.4 | 87.4 | |  |
| [19] | Serranía de Pirre, w, IT | | 1,260 | -77.7213 | 7.7643 | 1,292 | 1.707 | 82.0 | 87.8 | |  |
| [19] | Serrania de Pirre, Cerro Cituro, w, IT | | 900 | -77.6919 | 7.9397 | 914 | 1.707 | 82.0 | 86.4 | |  |
| [19] | Cerro Tacarcuna, w, IT | | 1,418 | -77.2833 | 8.1714 | 1,416 | 1.817 | 82.0 | 86.3 | |  |
| [19] | Cerro Campana, l, PC | | 870 | -79.9247 | 8.6808 | 879 | 1.152 | 80.9 | 86.5 | |  |
| [19] | Cerro Hoya, w, PC | | 910 | -80.6542 | 7.3437 | 952 | 1.533 | 80.0 | 85.5 | |  |
| [19] | Cordillera de Talamanca, Panama, l, IT | | 2,100 | -82.7324 | 8.9146 | 2,096 | 3.44 | 80.6 | 90.0 | |  |
| [19] | Cordillera de Talamanca, Panama, w, IT | | 1,800 | -82.6814 | 8.9596 | 1,800 | 3.468 | 84.2 | 88.3 | |  |
| [20-22] | Sierra Maestra - Pico Turquino, w, CC | | 900 | -76.8105 | 20.0067 | 903 | 1.946 | 77.3 | 82.4 | |  |
| [23] | Blue Mountains, w, CC | | 912 | -76.4977 | 18.0398 | 908 | 2.201 | 80.0 | 85.5 | |  |
| [23] | John Crow Mountains, l, CC | | 762 | -76.3477 | 18.0040 | 770 | 1.301 | 80.6 | 85.3 | |  |
| [24] | Cordillera Central, Pico Duarte, w, IT | | 1,700 | -70.9020 | 19.0334 | 1,697 | 3.098 | 73.3 | 79.5 | |  |
| [25] | Luquillo Mountains, w, CC | | 600 | -65.7585 | 18.2561 | 596 | 1.016 | 82.0 | 87.9 | |  |
| [26, 27] | Cordillera Central, Cerro La Santa, l, CC | | 785 | -66.0586 | 18.1065 | 783 | 0.919 | 80.6 | 85.0 | |  |
| [26] | Cordillera Central, Cerro de Punta, wl, CC | | 875 | -66.5420 | 18.2227 | 871 | 1.237 | 79.1 | 87.2 | |  |
| [28] | Mt. Scenery, w, DC | | 500 | -63.2331 | 17.6374 | 534 | 0.826 | 69.3 | 78.7 | |  |
| [29] | Nevis Peak, wl, CC | | 700 | -62.5814 | 17.1455 | 715 | 0.75 | 78.1 | 81.4 | |  |
| [29] | Mount Liamuiga, l, CC | | 800 | -62.8127 | 17.3722 | 808 | 0.995 | 78.4 | 83.5 | |  |
| [29] | Mount Liamuiga, w, CC | | 700 | -62.7952 | 17.3639 | 707 | 1.031 | 78.3 | 82.1 | |  |
| [30, 31] | Morne Tres Pitons, w, CC | | 785 | -61.3293 | 15.3810 | 787 | 1.344 | 82.5 | 92.0 | |  |
| [29] | Mount Saint Catherine, w, CC | | 590 | -61.6562 | 12.1622 | 583 | 0.693 | 80.8 | 83.3 | |  |
| [29] | Mount Saint Catherine, l, CC | | 650 | -61.6895 | 12.1580 | 658 | 0.711 | 80.7 | 83.7 | |  |
| [32] | El Cerro del Aripo, l, CC | | 725 | -61.2496 | 10.7228 | 725 | 0.933 | 80.8 | 86.8 | |  |
| [33, 34] | Guiana Highlands, Mt Roraima, wl, IT | | 1,500 | -60.7646 | 5.2078 | 1,543 | 2.525 | 81.0 | | 85.0 | |
| **S3 Table cont’d** | |  |  |  |  |  |  |  | |  | |
| **Source** | **Location, Aspect, Region** | | **CFmin (m)** | **Longitude (dd)** | **Latitude (dd)** | **CFmin Cell (m)** | **ELEVmax (km)** | **RH150 (%)** | | **RH (%)** | |
| [35] | Coastal Ecuador, Cerro Montecristi, w, DC | | 520 | -80.6709 | -1.0539 | 555 | 0.606 | 76.1 | | 74.9 | |
| [36] | Serra do Caparaó, l, IT | | 1,700 | -41.8309 | -20.4247 | 1,733 | 2.676 | 76.0 | | 84.0 | |
| [37] | Serra do Mar mountains, w, IT | | 1,150 | -48.7992 | -25.2453 | 1,162 | 2.029 | 80.3 | | 84.2 | |
| [38] | Serra Geral, Serra do Mar border, l, IT | | 1,500 | -49.4930 | -28.1068 | 1,491 | 1.761 | 76.9 | | 85.2 | |
| [33, 34] | Cordillera Araya-Paria, w, IT | | 1,000 | -62.6271 | 10.7124 | 1,001 | 1.223 | 76.6 | | 84.1 | |
| [33, 34] | Cordillera de la Costa oceanfront, w, DC | | 800 | -66.7981 | 10.5897 | 822 | 2.733 | 77.3 | | 76.9 | |
| [33, 34] | Cordillera de la Costa, w, IT | | 1,200 | -67.7186 | 10.3398 | 1,215 | 2.733 | 77.6 | | 77.2 | |
| [33, 34] | Serrania del Interior Central, w, IT | | 1,200 | -66.3626 | 10.0311 | 1,232 | 1.505 | 77.6 | | 79.5 | |
| [33, 34] | Serrania del Interior Oriental, w, IT | | 1,800 | -64.0953 | 10.0959 | 1,847 | 2.582 | 76.6 | | 87.1 | |
| [33, 34] | Margarita Peak, w, DC | | 600 | -63.8938 | 11.0124 | 614 | 0.895 | 75.5 | | 79.1 | |
| [33, 34] | Sierra de San Luis, Falcón y Laura, wl, IT | | 1,050 | -69.6958 | 11.2068 | 1,075 | 1.489 | 76.8 | | 77.7 | |
| [33, 34] | Paraguaná Peninsula, w, DC | | 550 | -69.9476 | 11.8207 | 579 | 0.75 | 75.2 | | 76.0 | |
| [33, 34] | Cordillera de Merida, wl, IT | | 1,750 | -70.6667 | 8.6666 | 1,733 | 4.856 | 77.1 | | 79.0 | |
| [33, 34] | Sierra de Perija, w, IT | | 1,400 | -72.7771 | 10.5372 | 1,456 | 3.617 | 76.9 | | 84.0 | |
| [39] | Sierra Nevada de Santa Marta, l, IT | | 1,900 | -74.0020 | 10.8770 | 1,885 | 5.683 | 76.9 | | 90.0 | |
| [40] | Serranía de Macuira, w, DC | | 525 | -71.3801 | 12.1723 | 534 | 0.729 | 74.9 | | 75.0 | |
| EC | Andes Cordillera Oriental, Ecuador, w, IT | | 1,500 | -77.8101 | -0.6792 | 1,505 | 5.834 | 82.3 | | 91.9 | |
| [41, 42] | Andes Cordillera El Consuelo, w, IT | | 1,850 | -79.0603 | -3.9726 | 1,901 | 3.774 | 82.3 | | 82.4 | |
| EC | Andes Cordillera del Condor, l, IT | | 1,100 | -78.3709 | -3.7583 | 1,100 | 2.681 | 82.3 | | 84.9 | |
| [43] | Andes Huancabamba Depression, w, IT | | 1,700 | -78.4366 | -5.5461 | 1,734 | 3.947 | 82.3 | | 78.3 | |
| [44] | Andes Cordillera Willkabamba, w, IT | | 1,650 | -71.5520 | -13.0585 | 1,688 | 6.386 | 77.7 | | 80.6 | |
| [46] | Andes Cordillera Willkabamba N. HP, w, IT | | 1,380 | -73.7145 | -12.5835 | 1,373 | 5.624 | 82.3 | | 72.0 | |
| [45, 46] | Andes Río Abiseo National Park, w, IT | | 1,500 | -76.9355 | -7.5603 | 1,543 | 4.427 | 82.3 | | 75.1 | |
| [47-50] | Andes Cerro Jaccha Hornuni, w, IT | | 2,000 | -67.9231 | -16.1290 | 2,011 | 6.386 | 77.7 | | 75.5 | |
| [51] | Andes Anconquija, w, OT | | 1,500 | -65.6246 | -27.0191 | 1,521 | 5.519 | 61.5 | | 73.6 | |
| Img | Andes Cordillera Occidental 1, w, IT | | 625 | -76.5496 | 6.3069 | 635 | 1.545 | 89.8 | | 91.1 | |
| Img | Andes Cordillera Occidental 2, l, IT | | 975 | -76.5139 | 6.3405 | 970 | 1.545 | 89.8 | | 91.3 | |
| Img | Andes Cordillera Occidental 3, w, IT | | 1,700 | -76.2730 | 5.9496 | 1,710 | 4.032 | 89.8 | | 91.2 | |
| Img | Andes Cordillera Occidental 4, w, IT | | 1,150 | -77.6562 | 2.0503 | 1,149 | 1.517 | 91.2 | | 88.0 | |
| Img | Andes Cordillera Occidental 5, w, IT | | 1,250 | -76.3419 | 5.1209 | 1,246 | 4.056 | 92.1 | | 89.8 | |
| [52] | Santa Cruz Island, Galapagos, w, DC | | 450 | -90.2919 | -0.6530 | 446 | 0.859 | 74.5 | | 77.7 | |
| [53] | Andes Carrasco National Park, w, IT | | 1,800 | -63.8638 | -18.8283 | 1,806 | 6.386 | 77.7 | | 75.5 | |
| [54] | Andes Los Toldos Valley, w, OT | | 1,600 | -64.6955 | -22.3720 | 1,549 | 6.345 | 67.8 | | 62.5 | |
| Img | Andes Cordillera Occidental 6, w, IT | | 825 | -76.5979 | 6.6935 | 824 | 1.493 | 89.8 | | 88.5 | |
| Img | Andes Cordillera Occidental 7, w, IT | | 900 | -76.5686 | 4.7205 | 889 | 4.056 | 92.1 | | 87.6 | |
| Img | Andes Cordillera Occidental 8, w, IT | | 1,225 | -76.7274 | 3.8905 | 1,224 | 3.911 | 91.2 | | 79.0 | |
| Img | Andes Cordillera Oriental 1, w, IT | | 1,600 | -71.9322 | 6.7221 | 1,564 | 5.318 | 79.0 | | 89.7 | |
| Img | Andes Cordillera Oriental 2, w, IT | | 1,500 | -71.9055 | 6.6268 | 1,504 | 5.122 | 79.9 | | 89.2 | |
| Img | Andes Cordillera Occidental 9, w, IT | | 1,300 | -75.9930 | 7.3287 | 1,303 | 3.682 | 78.0 | | 84.7 | |

Img = CF_min_ from image interpretation (see main text). EC = Expert consultation. Column definitions in Table 3.

## References

1. Martin PS. Zonal distribution of vertebrates in a Mexican cloud forest. American Naturalist. 1955;89(849):347-61.

2. Williams Linera G, Pérez García I, Tolome J. El bosque mesófilo de montaña y un gradiente altitudinal en el Centro de Veracruz, México. La Ciencia y el Hombre. 1996;23:149-61.

3. Sánchez-Cordero V. Elevation gradients of diversity for rodents and bats in Oaxaca, Mexico. Global Ecology and Biogeography. 2001;10(1):63-76. doi: 10.1046/j.1466-822x.2001.00235.x.

4. Mejía Domínguez NR, Meave JA, Ruiz Jiménez CA. Análisis estructural de un bosque mesófilo de montaña en el extremo oriental de la Sierra Madre del Sur (Oaxaca), México. Boletín de la Sociedad Botánica de México. 2004;(74).

5. Arteaga GÁ, Calderón NG, Krasilnikov P, Sedov S, Targulian V, Rosas NV. Soil altitudinal sequence on base-poor parent material in a montane cloud forest in Sierra Juárez, Southern Mexico. Geoderma. 2008;144(3):593-612.

6. Vazquez-García JA, Givnish TJ. Altitudinal Gradients in Tropical Forest Composition, Structure, and Diversity in the Sierra de Manantlan. Journal of Ecology. 1998;86(6):999-1020. doi: 10.2307/2648664.

7. Tejeda‐Cruz C, Sutherland WJ. Cloud forest bird responses to unusually severe storm damage. Biotropica. 2005;37(1):88-95.

8. Leopold AS. Vegetation Zones of Mexico. Ecology. 1950;31(4):507-18. doi: 10.2307/1931569.

9. Campbell JA. The biogeography of the cloud forest herpetofauna of Middle America, with special reference to the Sierra de las Minas of Guatemala: University of Kansas; 1982.

10. Land HC. A collection of birds from the Sierra de las Minas, Guatemala. The Wilson Bulletin. 1962:267-83.

11. Holder CD. Rainfall interception and fog precipitation in a tropical montane cloud forest of Guatemala. Forest Ecology and Management. 2004;190(2–3):373-84. doi: <http://dx.doi.org/10.1016/j.foreco.2003.11.004>.

12. Caballero LA. Hydrology, hydrochemistry and implications for water supply of a cloud forest in Central America. Ithaca, NY USA: Cornell University; 2012.

13. Carr AF. Outline for a classification of animal habitats in Honduras. Bulletin of the American Museum of Natural History. 1950;94:567-94.

14. Hollenbeck E. Plants and Vegetation — Update 2014. In: Nadkarni NM, Wheelwright NT, editors. Monteverde: Ecology and Conservation of a Tropical Cloud Forest - 2014 Updated Chapters: Bowdoin Scholars' Bookshelf, Book 4; 2014.

15. Nadkarni NM, Solano R. Potential effects of climate change on canopy communities in a tropical cloud forest: an experimental approach. Oecologia. 2002;131(4):580-6. doi: 10.1007/s00442-002-0899-3.

16. Lieberman D, Milton L, Peralta R, Hartshorn GS. Tropical Forest Structure and Composition on a Large-Scale Altitudinal Gradient in Costa Rica. Journal of Ecology. 1996;84(2):137-52. doi: 10.2307/2261350.

17. Helmer EH. The landscape ecology of tropical secondary forest in montane Costa Rica. Ecosystems. 2000;3(1):98-114.

18. Kappelle M, Juárez ME. Land Use, Ethnobotany and Conservation in Costa Rican Montane Oak Forests. In: Kappelle M, editor. Ecology and Conservation of Neotropical Montane Oak Forests. Ecological Studies. 185: Springer Berlin Heidelberg; 2006. p. 393-406.

19. Myers CW. The ecological geography of cloud forest in Panama. American Museum Novitates. 1969;2396:1-52.

20. Borhidi A. Phytogeography and vegetation ecology of Cuba. Akadémiai kiadó. Budapest Rumanía. 1991.

21. Carabia JP. The Vegetation of Sierra de Nipe, Cuba. Ecological Monographs. 1945;15(4):322-41. doi: 10.2307/1948426.

22. Pócs T. Tropical Forest Bryophytes. In: Smith AJE, editor. Bryophyte Ecology: Springer Netherlands; 1982. p. 59-104.

23. Asprey GF, Robbins RG. The Vegetation of Jamaica. Ecological Monographs. 1953;23(4):359-412. doi: 10.2307/1948625.

24. Sherman RE, Martin PH, Fahey TJ. Vegetation-environment relationships in forest ecosystems of the Cordillera Central, Dominican Republic. The Journal of the Torrey Botanical Society. 2005;132(2):293-310. doi: 10.3159/1095-5674(2005)132[293:vrifeo]2.0.co;2.

25. Weaver PL. Environmental gradients affect forest structure in Puerto Rico's Luquillo Mountains. Interciencia. 2000;25(5):254-9.

26. Helmer EH, Ramos O, López TdM, Quiñones M, Diaz W. Mapping forest type and land cover of Puerto Rico, a component of the Caribbean biodiversity hotspot. Caribbean Journal of Science. 2002;38(3-4):165-83.

27. Weaver PL. Baño de Oro Natural Area, Luquillo Mountains, Puerto Rico. In: U.S. Department of Agriculture FS, editor. New Orleans, LA USA: Southern Forest Research Station; 1994. p. 55.

28. Chipka SA, Izquierdo JA. A New Catalogue of Orchidaceae for Saba, N.A. Selbyana. 2005;26(1/2):14-22.

29. Helmer EH, Kennaway TA, Pedreros DH, Clark ML, Marcano-Vega H, Tieszen LL, et al. Land cover and forest formation distributions for St. Kitts, Nevis, St. Eustatius, Grenada and Barbados from decision tree classification of cloud-cleared satellite imagery. Caribbean Journal of Science. 2008;44(2):175-98.

30. Beard JS. The natural vegetation of the Windward & Leeward Islands: Clarendon Press; 1949.

31. Coan M, Wood E, Reillo P, cartographers. Land cover map of Dominica. Sioux Falls, SD, USA: US Geological Survey; 2007.

32. Helmer EH, Ruzycki TS, Benner J, Voggesser SM, Scobie BP, Park C, et al. Detailed maps of tropical forest types are within reach: Forest tree communities for Trinidad and Tobago mapped with multiseason Landsat and multiseason fine-resolution imagery. Forest Ecology and Management. 2012;279:147-66. doi: <https://doi.org/10.1016/j.foreco.2012.05.016>.

33. Ataroff M. Venezuela. In: Kappelle M, Brown AD, editors. Bosques nublados del neotrópico. San José, Costa Rica: Instituto Nacional de Biodiversidad; 2001. p. 397-442.

34. Ataroff M. Selvas y bosques de montaña. In: Aguilera M, Azózar A, González-Jiménez E, editors. Biodiversidad en Venezuela. Caracas, Venezuela: FONACIT-Fundación Polar; 2003. p. 762-811.

35. Dodson CH, Gentry AH. Biological extinction in western Ecuador. Annals of the Missouri Botanical Garden. 1991:273-95.

36. Safford HD. Brazilian Páramos I. An introduction to the physical environment and vegetation of the campos de altitude. Journal of Biogeography. 1999;26(4):693-712.

37. de Barcellos Falkenberg D, Voltolini J. The Montane Cloud Forest in Southern Brazil. In: Hamilton L, Juvik J, Scatena FN, editors. Tropical Montane Cloud Forests. Ecological Studies. 110: Springer US; 1995. p. 138-49.

38. Higuchi P, Silva ACd, Ferreira TS, Souza STd, Gomes JP, Silva KMd, et al. Floristic composition and phytogeography of the tree component of Araucaria Forest fragments in southern Brazil. Brazilian Journal of Botany. 2012;35(2):145-57.

39. Sugden AM, Robins RJ. Aspects of the Ecology of Vascular Epiphytes in Colombian Cloud Forests, I. The Distribution of the Epiphytic Flora. Biotropica. 1979;11(3):173-88. doi: 10.2307/2388037.

40. Sugden AM. Long-Distance Dispersal, Isolation, and the Cloud Forest Flora of the Serrania de Macuira, Guajira, Colombia. Biotropica. 1982;14(3):208-19. doi: 10.2307/2388027.

41. Brehm G, Fiedler K. Faunal composition of geometrid moths changes with altitude in an Andean montane rain forest. Journal of Biogeography. 2003;30(3):431-40.

42. Bussmann RW. The montane forests of Reserva Biologica San Francisco (Zamora-Chinchipe, Ecuador)-vegetation zonation and natural regeneration. Die Erde. 2001;132(1):9-25.

43. Young KR, Reynel C. Huancabamba Region, Peru and Ecuador. In: Davis SD, Heywood VH, Herrera-Acbryde O, Villa-Lobos J, Hamilton AC, editors. Centres of Plant Diversity: A Guide and Strategy for their Conservation. 3, The Americas. Cambridge, UK: The World Wide Fund For Nature (WWF) and IUCN - The World Conservation Union, IUCN Publications Unit; 1997. p. 465-9.

44. Girardin CAJ, Malhi Y, Aragão LEOC, Mamani M, Huaraca Huasco W, Durand L, et al. Net primary productivity allocation and cycling of carbon along a tropical forest elevational transect in the Peruvian Andes. Global Change Biology. 2010;16(12):3176-92. doi: 10.1111/j.1365-2486.2010.02235.x.

45. Leo M. The Importance of Tropical Montane Cloud Forest for Preserving Vertebrate Endemism in Peru: The Río Abiseo National Park as a Case Study. In: Hamilton L, Juvik J, Scatena FN, editors. Tropical Montane Cloud Forests. Ecological Studies. 110: Springer US; 1995. p. 198-211.

46. Young KR. Tropical timberlines: changes in forest structure and regeneration between two Peruvian timberline margins. Arctic and Alpine research. 1993:167-74.

47. Bach K. Vegetation, soil and climate in different altitudinal belts in montane cloud forests of the Yungas, Bolivia: first results. Ecología en Bolivia-Revista del Instituto de Ecología. 2003;38(1):3-14.

48. Bach K, Kessler M, Gradstein SR. A simulation approach to determine statistical significance of species turnover peaks in a species-rich tropical cloud forest. Diversity and Distributions. 2007;13(6):863-70. doi: 10.1111/j.1472-4642.2007.00357.x.

49. Gerold G, Schawe M, Bach K. Hydrometeorologic, pedologic and vegetation patterns along an elevational transect in the montane forest of the Bolivian Yungas. Die Erde. 2008;139(1-2):141-68.

50. Paniagua Zambrana N, Maldonado Goyzueta C, Chumacero Moscoso C. Mapa de vegetación de los alrededores de la Estación Biológica de Tunquini, Bolivia. Ecología en Bolivia. 2003;38(1):15-26.

51. Halloy S. Anconquija Region, North-western Argentina. Centres of Plant Diversity A guide and strategy for their conservation Cambridge, UK: WWF, IUCN. 1997:478-85.

52. Pryet A, Domínguez C, Tomai PF, Chaumont C, d’Ozouville N, Villacís M, et al. Quantification of cloud water interception along the windward slope of Santa Cruz Island, Galapagos (Ecuador). Agricultural and Forest Meteorology. 2012;161:94-106. doi: <http://dx.doi.org/10.1016/j.agrformet.2012.03.018>.

53. Kessler M. Elevational gradients in species richness and endemism of selected plant groups in the central Bolivian Andes. Plant Ecology. 2000;149(2):181-93. doi: 10.1023/a:1026500710274.

54. Carilla J, Grau HR. 150 Years of Tree Establishment, Land Use and Climate Change in Montane Grasslands, Northwest Argentina. Biotropica. 2010;42(1):49-58. doi: 10.1111/j.1744-7429.2009.00565.x.

S3 Table of Supporting material for:

E. H. Helmer, E. A. Gerson, L. Scott Baggett, Benjamin J. Bird, Thomas S. Ruzycki, Shannon M. Voggesser. 2019. Neotropical cloud forests and páramo to contract and dry from declines in cloud immersion and frost. 2019. PLOS ONE.

Raster data available at: <https://doi.org/10.2737/RDS-2019-0008>
