# Supplementary material for: Neotropical cloud forests and páramo to contract and dry from declines in cloud immersion and frost
Source: PLoS One. 2019 Apr 17;14(4):e0213155. doi: 10.1371/journal.pone.0213155 (PMC6469753; doi:10.1371/journal.pone.0213155)
Supplement: S6 Table — (DOCX) [file pone.0213155.s011.docx]

**S6 Table. Neotropical and regional cloud immersion changes under RCP 8.5, 2041-2060**. Results project that 80% of Neotropical TMCF will experience cloud immersion declines with worst-case scenario climate change as early as around 2040 (2041-2060, average year 2050) for Representative Concentration Pathway 4.5, *i.e.,* RCP 4.5. The net loss of area with a TMCF climate in the Neotropics is 80% minus 4.6% ≈ 75%. Changes in cloud immersion are given by **change category ^a^** and TMCF upper limit types as percentages of TMCF zone areas ^b^. **Upper limit types**: **No Subalpine (or N)** = montane TMCF where no subalpine occurs, **Mixed (or Mxd)** = montane + mixed TMCF, **Subalpine 1 (or S1)** = montane + subalpine 1 TMCF, **Subalpine 2 (or S2)** = montane + subalpine 2 TMCF. **Subalpine 1** = TMCF transitions to páramo;

**Subalpine 2** = TMCF transitions to puna.

| **Region** | **Subalpine Type** | **Montane + Subalpine TMCF Zone Area (km^2^)^b^** | **Below CF_min_**  **(%)** | **RH­_d_ ≤ -3% or**  **RH<Rh_min_**  **(%)** | **-3%< RH_d_ <0%**  **(%)** | **RH_d_ ≥ 0%**  **Total Lost**  **(%)** | **RH_d_ ≥ 0%**  **Remaining**  **(%)** | **RH_d_** **≥ 0%**  **Added**  **(%)** | **RH_d_ ≥ 0%**  **Net**  **Remaining**  **(%)** |
| --- | --- | --- | --- | --- | --- | --- | --- | --- | --- |
| **Caribbean** | Mixed | 2,125 | 9.2 | 91 | 0.0 | 100 | 0 | 0 | 0 |
|  | No Subalpine | 1,354 | 20 | 68 | 12 | 100 | 0 | 0 | 0 |
| **Mesoamerica** | Subalpine 1 | 7,597 | 8.5 | 4.8 | 87 | 100 | 0 | 0 | 0 |
|  | Mixed | 46,540 | 17 | 53 | 31 | 100 | 0 | 0 | 0 |
|  | No Subalpine | 2,022 | 26 | 8.2 | 66 | 100 | 0 | 0 | 0 |
| **South America** | Subalpine 1 | 196,300 | 7.8 | 1.4 | 71 | 80 | 20 | 4.5 | 25 |
|  | Subalpine 2 | 92,470 | 13 | 0.72 | 59 | 72 | 28 | 5.4 | 33 |
|  | Mixed | 45,610 | 21 | 13 | 35 | 70 | 30 | 9.3 | 39 |
|  | No Subalpine | 7,367 | 21 | 7.4 | 54 | 82 | 18 | 2.5 | 21 |
| **Neotropics** | **All** | **401,400** | **12** | **9.5** | **59** | **80** | **20** | **4.6** | **25** |

**^a^Change categories**: Below CF_min_ = falls below CF­_min_ (other categories remain above CF_min)_; RH_d_ ≤ -3% or < RH_min_ = RH falls severely; -3% < RH_d_ < 0% = RH falls up to 3%; RH_d_ ≥ 0% = RH is stable or increases. ^b^Based on maps with a ~250-m cell size.
