# Supplementary material for: Neotropical cloud forests and páramo to contract and dry from declines in cloud immersion and frost
Source: PLoS One. 2019 Apr 17;14(4):e0213155. doi: 10.1371/journal.pone.0213155 (PMC6469753; doi:10.1371/journal.pone.0213155)
Supplement: S7 Table — (DOCX) [file pone.0213155.s012.docx]

**S7 Table. Neotropical and regional cloud immersion changes under RCP 8.5, 2061-2080**. Results project that 86% of Neotropical TMCF will experience cloud immersion declines with worst-case scenario climate change as early as around 2060 (2061-2080, average year 2070) for Representative Concentration Pathway 4.5, *i.e.,* RCP 4.5. The net loss of area with a TMCF climate in the Neotropics is 86% minus 4.8% ≈ 81%. Changes in cloud immersion are given by **change category ^a^** and TMCF upper limit types as percentages of TMCF zone areas ^b^. **Upper limit types**: **No Subalpine (or N)** = montane TMCF where no subalpine occurs, **Mixed (or Mxd)** = montane + mixed TMCF, **Subalpine 1 (or S1)** = montane + subalpine 1 TMCF, **Subalpine 2 (or S2)** = montane + subalpine 2 TMCF. **Subalpine 1** = TMCF transitions to páramo;

**Subalpine 2** = TMCF transitions to puna.

| **Region** | **Subalpine Type** | **Montane + Subalpine TMCF Zone Area (km^2^)^b^** | **Below CF_min_**  **(%)** | **RH­_d_ ≤ -3% or**  **RH<Rh_min_**  **(%)** | **-3%< RH_d_ <0%**  **(%)** | **RH_d_ ≥ 0%**  **Total Lost**  **(%)** | **RH_d_ ≥ 0%**  **Remaining**  **(%)** | **RH_d_** **≥ 0%**  **Added**  **(%)** | **RH_d_ ≥ 0%**  **Net**  **Remaining**  **(%)** |
| --- | --- | --- | --- | --- | --- | --- | --- | --- | --- |
| **Caribbean** | Mixed | 2,125 | 12 | 88 | 0 | 100 | 0 | 0 | 0 |
|  | No Subalpine | 1,354 | 25 | 75 | 0.0 | 100 | 0 | 0 | 0 |
| **Mesoamerica** | Subalpine 1 | 7,597 | 13 | 15 | 71 | 100 | 0 | 0 | 0 |
|  | Mixed | 46,540 | 23 | 70 | 6.7 | 100 | 0 | 0 | 0 |
|  | No Subalpine | 2,022 | 38 | 22 | 40 | 100 | 0 | 0 | 0 |
| **South America** | Subalpine 1 | 196,300 | 14 | 4.6 | 68 | 87 | 13 | 4.4 | 17 |
|  | Subalpine 2 | 92,470 | 22 | 7.2 | 55 | 84 | 16 | 4.2 | 20 |
|  | Mixed | 45,610 | 30 | 15 | 25 | 70 | 30 | 15 | 45 |
|  | No Subalpine | 7,367 | 34 | 14 | 34 | 82 | 18 | 1.9 | 20 |
| **Neotropics** | **All** | **401,400** | **19** | **15** | **52** | **86** | **14** | **4.8** | **19** |

**^a^Change categories**: Below CF_min_ = falls below CF­_min_ (other categories remain above CF_min)_; RH_d_ ≤ -3% or < RH_min_ = RH falls severely; -3% < RH_d_ < 0% = RH falls up to 3%; RH_d_ ≥ 0% = RH is stable or increases. ^b^Based on maps with a ~250-m cell size.
