# Supplementary material for: Neotropical cloud forests and páramo to contract and dry from declines in cloud immersion and frost
Source: PLoS One. 2019 Apr 17;14(4):e0213155. doi: 10.1371/journal.pone.0213155 (PMC6469753; doi:10.1371/journal.pone.0213155)
Supplement: S8 Table — (DOCX) [file pone.0213155.s013.docx]

S8 Table. Changes in montane TMCF cloud immersion by ecoregion, and forest cover and protection of the least affected change category, Mesoamerica and the Caribbean. For RCP-4.5, 100% of TMCF in Mesoamerican and Caribbean ecoregions suffer cloud immersion declines. Shown here are changes in TMCF cloud immersion by ecoregion, and status of forest cover and protection of the least affected change category, for average year ~2070 (years 2061-2080) under RCP-4.5. The class least affected by climate change in Mesomerican ecoregions is -3% < RH_d_ < 0. Proportions for this class are then broken down by forest cover and protection status.

| **Ecoregion** | **Type** | **RH_d_**  **(%)** | **Area (km^2^)** | **Projected percent of TMCF Zone Area** | | | | | | **Land cover and Protection of Least Affected Class**  **(% of TMCF Zone Area)**  **(Sums to Total % for**  **-3%<RH_d_** **<0%)** | | | | |
| --- | --- | --- | --- | --- | --- | --- | --- | --- | --- | --- | --- | --- | --- | --- |
|  |  |  |  | **Below CF_min_** | **RH­_d_ ≤**  **-3% or <Rh_min_** | **-3%<**  **RH_d_**  **<0%** | **RH_d_ ≥ 0%** | **New > CF_min_** | **New RH_d_ ≥ 0%** |  |  |  |  |  |
|  |  |  |  |  |  |  |  |  |  | **NFor UPR** | **NFor PR** | **For UPR** | | **For PR** |
| **Cuban moist forests** | N | -2.0 | 551 | 11 | 50 | 38 | 0 | 0 | 0 | 0 | 0.023 | 7.4 | 31 | |
| **Hispaniolan moist, pine and mixed forests** | MX | -3.3 | 1,136 | 16 | 79 | 4.8 | 0 | 0 | 0 | 0.8 | 0 | 4 | 0 | |
| **Jamaican moist forests** | N | -2.3 | 352 | 16 | 70 | 15 | 0 | 0 | 0 | 0.089 | 0.3 | 0.36 | 14 | |
| **Puerto Rican moist forests** | N | -3.6 | 261 | 26 | 74 | 0 | 0 | 0 | 0 | 0 | 0 | 0 | 0 | |
| **Leeward Islands moist forests^d^** | M | -2.9 | 7.6 | 23 | 74 | 3.3 | 0 | 0 | 0 | 0 | 0 | 0 | 2.5 | |
| **Windward Islands and Guadeloupe^d^** | N | -4.3 | 166 | 30 | 70 | 0 | 0 | 0 | 0 | 0 | 0 | 0 | 0 | |
| **Trinidad and Tobago moist forests** | N | -4.6 | 16 | 41 | 59 | 0 | 0 | 0 | 0 | 0 | 0 | 0 | 0 | |
| **Central American pine-oak, montane forests** | MX | -2.9 | 20,020 | 24 | 41 | 35 | 0 | 0 | 0 | 3.5 | 0.74 | 16 | 15 | |
| **Chiapas montane forests** | MX | -4.3 | 1,822 | 42 | 58 | 0 | 0 | 0 | 0 | 0 | 0 | 0 | 0 | |
| **Chimalapas montane forests** | N | -2.6 | 375 | 45 | 4.8 | 50 | 0 | 0 | 0 | 0.59 | 0 | 47 | 2.7 | |
| **Oaxacan montane forests** | MX | -2.7 | 3,364 | 16 | 59 | 25 | 0 | 0 | 0 | 1.2 | 0 | 24 | 0.26 | |
| **Sierra Madre Oriental pine-oak forests^d^** | MX | -2.6 | 1,186 | 15 | 48 | 37 | 0 | 0 | 0 | 0.84 | 0.037 | 33 | 3.2 | |
| **Sierra Madre de Oaxaca pine-oak forests** | MX | -2.8 | 1,883 | 11 | 53 | 35 | 0 | 0 | 0 | 2 | 0 | 33 | 0.23 | |
| **Sierra de los Tuxtlas** | N | -2.7 | 72 | 31 | 0.086 | 69 | 0 | 0 | 0 | 0 | 0.95 | 0 | 68 | |
| **Veracruz montane forests** | MX | -3.3 | 1,484 | 13 | 78 | 9 | 0 | 0 | 0 | 0.46 | 0.01 | 7.5 | 1.1 | |
| **Sierra Madre Occidental pine-oak forests^d^** | MX | -2.1 | 1,403 | 28 | 25 | 47 | 0 | 0 | 0 | 8.4 | 0 | 39 | 0.089 | |
| **Sierra Madre del Sur pine-oak forests** | MX | -2 | 4,389 | 13 | 12 | 75 | 0 | 0 | 0 | 5.4 | 0.01 | 69 | 0.25 | |
| **Trans-Mexican Volcanic Belt pine-oak forests** | MX | -2.7 | 1,469 | 19 | 64 | 16 | 0 | 0 | 0 | 1.7 | 0.13 | 12 | 2.2 | |
| **Choco-Darien moist coastal forest** | N | -0.88 | 772 | 19 | 0.58 | 80 | 0 | 0 | 0 | 0.032 | 0.41 | 37 | 43 | |
| **Guanacaste and Tilarán montane forests** | N | -1.5 | 112 | 34 | 0 | 66 | 0 | 0 | 0 | 0.056 | 0.5 | 4.6 | 60 | |
| **Cordillera de los Maribios forests^d^** | N | -2.4 | 8.1 | 22 | 44 | 34 | 0 | 0 | 0 | 0 | 32 | 0 | 1.5 | |
| **Eastern Panamanian montane forests** | N | -0.87 | 247 | 17 | 24 | 59 | 0 | 0 | 0 | 0.25 | 0.2 | 26 | 32 | |
| **Isthmian Pacific moist forests** | N | -1.1 | 436 | 17 | 15 | 68 | 0 | 0 | 0 | 1.8 | 0.59 | 23 | 42 | |
| **Talamancan and Central montane forests** | S1 | -1.1 | 7,403 | 8.1 | 3.9 | 88 | 0 | 0 | 0 | 3.4 | 0.7 | 30 | 54 | |

S2 Table defines RH_d_ and CF_min_. Forest cover and protection status include: NFor = Nonforest, For = Forest, UPR = Unprotected, PR = Protected. S4 Table defines TMCF upper limit Types. Superscript **d** indicates nonforest class includes significant deciduous forest, dry scrub, savanna, or fumarole vegetation, and not all forest absence equates to deforestation.
