# Supplementary material for: Neotropical cloud forests and páramo to contract and dry from declines in cloud immersion and frost
Source: PLoS One. 2019 Apr 17;14(4):e0213155. doi: 10.1371/journal.pone.0213155 (PMC6469753; doi:10.1371/journal.pone.0213155)
Supplement: S9 Table — (DOCX) [file pone.0213155.s014.docx]

S9 Table. Changes in montane TMCF cloud immersion by ecoregion, and forest cover and protection of the least affected change category, South America. Most South American ecoregions suffer cloud immersion declines. Shown here are changes in TMCF cloud immersion by ecoregion, and status of forest cover and protection of the least affected change category, for average year ~2070 (years 2061-2080) under RCP-4.5. The class least affected by climate change in South American ecoregions is RH_d_ ≥ 0%. Proportions for this class are then broken down by forest cover and protection status.

| **Ecoregion** | **Type** | **RH_d_**  **(%)** | **Area (km^2^)** | **Projected percent of TMCF Zone Area** | | | | | | **Land cover and Protection of Least Affected Class**  **(% of TMCF Zone Area)**  **(Sums to Total % for**  **RH_d_ ≥ 0%)** | | | |
| --- | --- | --- | --- | --- | --- | --- | --- | --- | --- | --- | --- | --- | --- |
|  |  |  |  | **Below CF_min_** | **RH­_d_ ≤**  **-3% or < Rh_min_** | **-3%<**  **RH_d_**  **<0%** | **RH_d_ ≥ 0%** | **New > CF_min_** | **New RH ≥ Rh_min_** |  |  |  |  |
|  |  |  |  |  |  |  |  |  |  | **NFor UPR** | **NFor PR** | **For UPR** | **For PR** |
| **Eastern Cordillera real montane forests** | S1 | -0.04 | 37,180 | 4.7 | 0.01 | 45 | 50 | 1.1 | 1.9 | 11 | 0.15 | 26 | 13 |
| **Eastern Cordillera real montane forests foothills** | N | -0.99 | 4,059 | 22 | 0 | 78 | 0.04 | 0 | 0 | 0.00 | 0 | 0.03 | 0.00 |
| **Cauca Valley montane forests** | S1 | -0.11 | 19,520 | 6.3 | 0.11 | 48 | 46 | 0 | 0 | 4.1 | 0.46 | 32 | 8.9 |
| **Cordillera Oriental montane forests** | S1 | -0.48 | 10,800 | 6.1 | 0 | 80 | 14 | 0 | 0 | 5 | 0.13 | 7.7 | 1.6 |
| **Cordillera Oriental montane forests north** | S1 | -0.38 | 13,570 | 6.4 | 0 | 55 | 38 | 0 | 0 | 9.5 | 0.58 | 24 | 4.3 |
| **Magdalena Valley montane forests** | S1 | -0.03 | 47,380 | 4.8 | 0 | 47 | 48 | 0 | 0.04 | 19 | 0.75 | 25 | 3.9 |
| **Northwestern Andean montane forests** | S1 | -0.31 | 33,880 | 1.7 | 0.59 | 70 | 28 | 0.8 | 0.76 | 9.3 | 0.098 | 15 | 3.4 |
| **Santa Marta montane forests** | S1 | -2.4 | 1,483 | 4 | 0.14 | 96 | 0 | 0 | 0 | 0 | 0 | 0 | 0 |
| **Venezuelan Andes montane forests** | S1 | -0.93 | 8,297 | 5.3 | 10 | 77 | 7.7 | 0 | 0 | 1.3 | 0.25 | 5 | 1.1 |
| **Cordillera de la Costa montane forests** | N | -2.2 | 1,676 | 13 | 5.4 | 81 | 0 | 8.2 | 0 | 0 | 0 | 0 | 0 |
| **Guajira-Barranquilla xeric scrub^d^** | N | 0.03 | 10 | 0 | 0 | 29 | 71 | 14 | 0 | 32 | 6.4 | 0 | 32 |
| **Guiana Highlands moist forests** | MX | -2.9 | 7,433 | 27 | 48 | 25 | 0 | 0 | 0 | 0 | 0 | 0 | 0 |
| **Isla Margarita** | N | -0.82 | 10 | 0 | 28 | 72 | 0 | 0 | 0 | 0 | 0 | 0 | 0 |
| **Lara-Falcon dry forests** | N | -0.99 | 73 | 8.8 | 0 | 91 | 0 | 0 | 0 | 0 | 0 | 0 | 0 |
| **Paraguana xeric scrub^d^** | N | -1.1 | 1.5 | 29 | 0 | 71 | 0 | 0 | 0 | 0 | 0 | 0 | 0 |
| **Ecuadorian dry forests^d^** | N | 0.15 | 381 | 0 | 0 | 14 | 86 | 17 | 0.15 | 2.9 | 1.2 | 69 | 13 |
| **Galapagos islands^d^** | N | 2.2 | 921 | 0 | 0 | 0 | 100 | 9 | 8.1 | 0 | 56 | 0 | 44 |
| **Alto Parana forests and Campos Rupestres^d^** | MX | -0.49 | 48 | 42 | 53 | 4.7 | 0 | 0 | 0 | 0 | 0 | 0 | 0 |
| **Araucaria moist forests^d^** | MX | 1.4 | 1,675 | 0.06 | 0.16 | 2.9 | 97 | 16 | 80 | 36 | 2.7 | 51 | 7.3 |
| **Serra do Mar coastal forests** | N | -0.71 | 236 | 2.4 | 43 | 45 | 9.6 | 0.42 | 0.13 | 0.82 | 0.08 | 8 | 0.69 |
| **Bolivian Yungas** | S2 | -1.6 | 17,390 | 18 | 2.5 | 79 | 0 | 0 | 0 | 0 | 0 | 0 | 0 |
| **Peruvian Yungas** | S2 | 0.25 | 35,680 | 5.5 | 0.14 | 20 | 74 | 0.26 | 1.1 | 5.3 | 0.19 | 54 | 15 |
| **Peruvian Yungas foothills** | S2 | -1.2 | 27,810 | 13 | 0.00 | 85 | 2.4 | 0 | 0.00 | 0.01 | 0.01 | 1.5 | 0.85 |
| **Southern Andean Yungas^d^** | MX | 0.09 | 28,040 | 24 | 0.73 | 39 | 37 | 0.95 | 1 | 7.4 | 0.95 | 20 | 8.5 |

S2 Table defines RH_d_ and CF_min_. Forest cover and protection status include: NFor = Nonforest, For = Forest, UPR = Unprotected, PR = Protected. S4 Table defines TMCF upper limit Types. Superscript **d** indicates nonforest class includes significant deciduous forest, dry scrub, savanna, or fumarole vegetation, and not all forest absence equates to deforestation.
