# Supplementary material for: Neotropical cloud forests and páramo to contract and dry from declines in cloud immersion and frost
Source: PLoS One. 2019 Apr 17;14(4):e0213155. doi: 10.1371/journal.pone.0213155 (PMC6469753; doi:10.1371/journal.pone.0213155)
Supplement: S10 Table — (DOCX) [file pone.0213155.s015.docx]

**S10 Table. Regional cloud immersion changes by protection status for RCP 4.5, 2041-2060** (Representative Concentration Pathway 4.5, average year 2050). Changes in cloud immersion are given by change category^a^ and protection status^b^ for: montane TMCF no subalpine occurs (No Subalpine), montane + mixed TMCF (Mixed), or montane + subalpine 1 or subalpine 2 TMCF, as percentages of TMCF zone areas^c^. Subalpine 1 TMCF transitions to páramo; Subalpine 2 TMCF transitions to puna.

| **Region** | **Subalpine Type** | **PR or UPR** | **Montane + Subalpine TMCF Zone Area (km^2^)^b^** | **Below CF_min_**  **(%)** | **RH­_d_ ≤ -3% or**  **RH<Rh_min_**  **(%)** | **-3%< RH_d_ <0%**  **(%)** | **RH_d_ ≥ 0%**  **Total Lost**  **(%)** | **RH_d_ ≥ 0%**  **Left**  **(%)** | **RH_d_** **≥ 0%**  **Added**  **(%)** | **RH_d_ ≥ 0%**  **Net Left**  **(%)** |  |
| --- | --- | --- | --- | --- | --- | --- | --- | --- | --- | --- | --- |
| **Caribbean** | Mixed | UPR | 676 | 16 | 13 | 71 | 100 | - | - | - | |
|  |  | **PR** | **1,449** | **4.6** | **52** | **43** | **100** | **-** | **-** | **-** | |
|  | No Subalpine | UPR | 454 | 25 | 52 | 23 | 100 | - | - | - | |
|  |  | **PR** | **900** | **12** | **52** | **36** | **100** | **-** | **-** | **-** | |
| **Mesoamerica** | Subalpine 1 | UPR | 2,978 | 9.2 | 3.3 | 88 | 100 | 0.0 | - | 0.0 | |
|  |  | **PR** | **4,619** | **4.9** | **1.2** | **94** | **100** | **0.0** | **-** | **0.0** | |
|  | Mixed | UPR | 36,520 | 16 | 34 | 50 | 100 | - | - | - | |
|  |  | **PR** | **9,878** | **12** | **25** | **63** | **100** | **-** | **-** | **-** | |
|  | No Subalpine | UPR | 1,047 | 27 | 5.5 | 67 | 100 | - | - | - | |
|  |  | **PR** | **975** | **15** | **6.9** | **78** | **100** | **-** | **-** | **-** | |
| **South America** | Subalpine 1 | UPR | 152,200 | 5.1 | 0.06 | 33 | 38 | 62 | 7.1 | 69 | |
|  |  | **PR** | **44,060** | **3.8** | **0.73** | **42** | **46** | **54** | **15** | **68** | |
|  | Subalpine 2 | UPR | 55,460 | 8.3 | 0.06 | 40 | 49 | 51 | 7.0 | 58 | |
|  |  | **PR** | **37,020** | **13** | **0.26** | **62** | **75** | **25** | **2.3** | **28** | |
|  | Mixed | UPR | 28,230 | 16 | 4.4 | 42 | 63 | 37 | 16 | 53 | |
|  |  | **PR** | **17,390** | **20** | **14** | **46** | **80** | **20** | **4.6** | **24** | |
|  | No Subalpine | UPR | 3,631 | 23 | 0.5 | 67 | 90 | 9.8 | 0.5 | 10 | |
|  |  | **PR** | **3,736** | **12** | **2.0** | **59** | **73** | **27** | **6.2** | **33** | |

^a^Change categories: Below CF_min_ = falls below CF­_min_ (other categories remain above CF_min_ ); RH_d_ ≤ -3% or < RH_min_ = RH falls severely; -3% < RH_d_ < 0% = RH falls up to 3%; RH_d_ ≥ 0% = RH is stable or increases. ^b^UPR = unprotected, PR = protected. ^c^Based on maps with a ~250-m cell size.
