# Supplementary material for: Neotropical cloud forests and páramo to contract and dry from declines in cloud immersion and frost
Source: PLoS One. 2019 Apr 17;14(4):e0213155. doi: 10.1371/journal.pone.0213155 (PMC6469753; doi:10.1371/journal.pone.0213155)
Supplement: S11 Table — (DOCX) [file pone.0213155.s016.docx]

**S11 Table. Regional cloud immersion changes by protection status for RCP 4.5, 2061-2080** (Representative Concentration Pathway 4.5, average year 2070). Changes in cloud immersion are given by change category^a^ and protection status^b^ for: montane TMCF no subalpine occurs (No Subalpine), montane + mixed TMCF (Mixed), or montane + subalpine 1 or subalpine 2 TMCF, as percentages of TMCF zone areas^c^. Subalpine 1 TMCF transitions to páramo; Subalpine 2 TMCF transitions to puna.

| **Region** | **Subalpine Type** | **PR or UPR** | **Montane + Subalpine TMCF Zone Area (km^2^)^b^** | **Below CF_min_**  **(%)** | **RH­_d_ ≤ -3% or**  **RH<Rh_min_**  **(%)** | **-3%< RH_d_ <0%**  **(%)** | **RH_d_ ≥ 0%**  **Total Lost**  **(%)** | **RH_d_ ≥ 0%**  **Left**  **(%)** | **RH_d_** **≥ 0%**  **Added**  **(%)** | **RH_d_ ≥ 0%**  **Net Left**  **(%)** |  |
| --- | --- | --- | --- | --- | --- | --- | --- | --- | --- | --- | --- |
| **Caribbean** | Mixed | UPR | 676 | 17 | 75 | 8.0 | 100 | - | - | - | |
|  |  | **PR** | **1,449** | **5.0** | **95** | **0.0** | **100** | **-** | **-** | **0.0** | |
|  | No Subalpine | UPR | 454 | 27 | 64 | 9.3 | 100 | - | - | 0.0 | |
|  |  | **PR** | **900** | **13** | **62** | **24** | **100** | **-** | **-** | **-** | |
| **Mesoamerica** | Subalpine 1 | UPR | 2,978 | 11 | 4.4 | 85 | 100 | - | - | - | |
|  |  | **PR** | **4,619** | **5.9** | **3.3** | **91** | **100** | **-** | **-** | **-** | |
|  | Mixed | UPR | 36,520 | 18 | 46 | 37 | 100 | - | - | - | |
|  |  | **PR** | **9,878** | **14** | **44** | **42** | **100** | **-** | **-** | **-** | |
|  | No Subalpine | UPR | 1,047 | 31 | 7.4 | 61 | 100 | - | - | - | |
|  |  | **PR** | **975** | **17** | **7.8** | **75** | **100** | **-** | **-** | **-** | |
| **South America** | Subalpine 1 | UPR | 152,200 | 4.3 | 0.23 | 50 | 55 | 45 | 6.9 | 52 | |
|  |  | **PR** | **44,060** | **3.1** | **2.2** | **60** | **66** | **34** | **14** | **49** | |
|  | Subalpine 2 | UPR | 55,460 | 7.8 | 0.4 | 45 | 54 | 46 | 7.2 | 54 | |
|  |  | **PR** | **37,020** | **12** | **0.8** | **69** | **82** | **18** | **2.3** | **20** | |
|  | Mixed | UPR | 28,230 | 20 | 5.3 | 41 | 66 | 34 | 14 | 48 | |
|  |  | **PR** | **17,390** | **21** | **24** | **38** | **83** | **17** | **4.6** | **22** | |
|  | No Subalpine | UPR | 3,631 | 20 | 2.0 | 70 | 92 | 8.2 | 1.8 | 10 | |
|  |  | **PR** | **3,736** | **10** | **3.3** | **60** | **74** | **26** | **7.9** | **34** | |

^a^Change categories: Below CF_min_ = falls below CF­_min_ (other categories remain above CF_min_ ); RH_d_ ≤ -3% or < RH_min_ = RH falls severely; -3% < RH_d_ < 0% = RH falls up to 3%; RH_d_ ≥ 0% = RH is stable or increases. ^b^UPR = unprotected, PR = protected. ^c^Based on maps with a ~250-m cell size.
