# Supplementary material for: Neotropical cloud forests and páramo to contract and dry from declines in cloud immersion and frost
Source: PLoS One. 2019 Apr 17;14(4):e0213155. doi: 10.1371/journal.pone.0213155 (PMC6469753; doi:10.1371/journal.pone.0213155)
Supplement: S12 Table — (DOCX) [file pone.0213155.s017.docx]

**S12 Table. Regional cloud immersion changes by protection status for RCP 8.5, 2041-2060** (Representative Concentration Pathway 8.5, average year 2050). Changes in cloud immersion are given by change category^a^ and protection status^b^ for: montane TMCF no subalpine occurs (No Subalpine), montane + mixed TMCF (Mixed), or montane + subalpine 1 or subalpine 2 TMCF, as percentages of TMCF zone areas^c^. Subalpine 1 TMCF transitions to páramo; Subalpine 2 TMCF transitions to puna.

| **Region** | **Subalpine Type** | **PR or UPR** | **Montane + Subalpine TMCF Zone Area (km^2^)^b^** | **Below CF_min_**  **(%)** | **RH­_d_ ≤ -3% or**  **RH<Rh_min_**  **(%)** | **-3%< RH_d_ <0%**  **(%)** | **RH_d_ ≥ 0%**  **Total Lost**  **(%)** | **RH_d_ ≥ 0%**  **Left**  **(%)** | **RH_d_** **≥ 0%**  **Added**  **(%)** | **RH_d_ ≥ 0%**  **Net Left**  **(%)** |  |
| --- | --- | --- | --- | --- | --- | --- | --- | --- | --- | --- | --- |
| **Caribbean** | Mixed | UPR | 676 | 18 | 82 | 0.0 | 100 | - | - | - | |
|  |  | **PR** | **1,449** | **5.0** | **95** | **0.0** | **100** | **-** | **-** | **-** | |
|  | No Subalpine | UPR | 454 | 29 | 66 | 4.8 | 100 | - | - | - | |
|  |  | **PR** | **900** | **15** | **69** | **16** | **100** | **-** | **-** | **-** | |
| **Mesoamerica** | Subalpine 1 | UPR | 2,978 | 12 | 5.6 | 83 | 100 | - | - | - | |
|  |  | **PR** | **4,619** | **6.4** | **4.3** | **89** | **100** | **-** | **-** | **-** | |
|  | Mixed | UPR | 36,520 | 17 | 53 | 30 | 100 | - | - | - | |
|  |  | **PR** | **9,878** | **13** | **51** | **36** | **100** | **-** | **-** | **-** | |
|  | No Subalpine | UPR | 1,047 | 34 | 7.3 | 59 | 100 | - | - | - | |
|  |  | **PR** | **975** | **18** | **9.2** | **73** | **100** | **-** | **-** | **-** | |
| **South America** | Subalpine 1 | UPR | 152,200 | 8.4 | 0.8 | 69 | 78 | 22 | 1.8 | 24 | |
|  |  | **PR** | **44,060** | **5.8** | **3.5** | **77** | **86** | **14** | **1.2** | **15** | |
|  | Subalpine 2 | UPR | 55,460 | 11 | 0.5 | 51 | 62 | 38 | 5.8 | 44 | |
|  |  | **PR** | **37,020** | **16** | **1.0** | **71** | **88** | **12** | **1.7** | **14** | |
|  | Mixed | UPR | 28,230 | 18 | 5.7 | 40 | 63 | 37 | 12 | 50 | |
|  |  | **PR** | **17,390** | **26** | **26.2** | **29** | **81** | **19** | **4.6** | **24** | |
|  | No Subalpine | UPR | 3,631 | 29 | 1.8 | 61 | 91 | 8.9 | 0.8 | 10 | |
|  |  | **PR** | **3,736** | **14** | **13** | **47** | **73** | **27** | **4.2** | **31** | |

^a^Change categories: Below CF_min_ = falls below CF­_min_ (other categories remain above CF_min_ ); RH_d_ ≤ -3% or < RH_min_ = RH falls severely; -3% < RH_d_ < 0% = RH falls up to 3%; RH_d_ ≥ 0% = RH is stable or increases. ^b^UPR = unprotected, PR = protected. ^c^Based on maps with a ~250-m cell size.
