# Supplementary material for: Neotropical cloud forests and páramo to contract and dry from declines in cloud immersion and frost
Source: PLoS One. 2019 Apr 17;14(4):e0213155. doi: 10.1371/journal.pone.0213155 (PMC6469753; doi:10.1371/journal.pone.0213155)
Supplement: S14 Table — (DOCX) [file pone.0213155.s019.docx]

S14 Table. TMCF zone forest cover and protection by ecoregion, Caribbean and Mesoamerica.

| **Ecoregion** | **Type** | **All TMCF**  **Forested Area (km2)** | **All TMCF Zone Area (km2)** | **UPR NFor (%)** | **PR NFor (%)** | **UPR For**  **(%)** | **PR For**  **(%)** | **Total % For** |
| --- | --- | --- | --- | --- | --- | --- | --- | --- |
| **Greater Antilles** |  |  |  |  |  |  |  |  |
| Cuban moist forests | N | 540 | 550 | 1.1 | 0.78 | 29 | 69 | 98 |
| Hispaniolan moist, pine and mixed forests | MX | 1,649 | 2,125 | 18 | 3.9 | 13 | 64 | 77 |
| Jamaican moist forests | N | 320 | 349 | 7.3 | 1.3 | 22 | 70 | 92 |
| Puerto Rican moist forests | N | 240 | 262 | 8.3 | 0.19 | 58 | 34 | 92 |
| **Lesser Antilles** |  |  |  |  |  |  |  |  |
| Leeward Islands moist forests^d^ | N | 7 | 8 | 14 | 0 | 77 | 9.4 | 86 |
| Windward Islands moist forests, Guadeloupe^d^ | N | 154 | 166 | 0 | 7.5 | 4.2 | 88 | 92 |
| **Trinidad and Tobago moist forests** |  |  |  |  |  |  |  |  |
| Trinidad moist forests | N | 16 | 16 | 0 | 0 | 88 | 12 | 100 |
| **Middle Central America** |  |  |  |  |  |  |  |  |
| Central American pine-oak, montane forests | MX | 19,120 | 23,210 | 16 | 2 | 56 | 26 | 82 |
| Chiapas montane forests | MX | 1,718 | 1,829 | 5.6 | 0.47 | 92 | 2.2 | 94 |
| Chimalapas montane forests | N | 373 | 376 | 0.82 | 0 | 93 | 6.1 | 99 |
| **Northeast Central America** |  |  |  |  |  |  |  |  |
| Oaxacan montane forests | MX | 2,992 | 3,435 | 12 | 0.5 | 85 | 2.2 | 87 |
| Sierra Madre Oriental pine-oak forests^d^ | MX | 2,205 | 2,763 | 17 | 2.8 | 51 | 29 | 80 |
| Sierra Madre de Oaxaca pine-oak forests | MX | 2,157 | 2,346 | 7.9 | 0.15 | 90 | 2 | 92 |
| Sierra de los Tuxtlas | N | 72 | 73 | 0 | 1.9 | 0 | 97 | 97 |
| Veracruz montane forests | MX | 1,570 | 1,919 | 15 | 3.4 | 72 | 9.6 | 82 |
| **Northwest Central America** |  |  |  |  |  |  |  |  |
| Sierra Madre Occidental pine-oak forests^d^ | MX | 1,043 | 1,403 | 22 | 3.5 | 70 | 4 | 74 |
| Sierra Madre del Sur pine-oak forests | MX | 3,927 | 4,455 | 12 | 0.091 | 87 | 1.2 | 88 |
| Trans-Mexican Volcanic Belt pine-oak forests | MX | 3,819 | 5,043 | 17 | 7.7 | 50 | 25 | 75 |
| **Southern Central America** |  |  |  |  |  |  |  |  |
| Choco-Darien moist coastal forest | N | 763 | 774 | 0.084 | 1.1 | 49 | 49 | 98 |
| Guanacaste and Tilarán montane forests | N | 110 | 111 | 0.2 | 0.93 | 10 | 89 | 99 |
| Cordillera de los Maribios forest^d^ | N | 5 | 8 | 0 | 36 | 0 | 64 | 64 |
| Eastern Panamanian montane forests | N | 243 | 247 | 0.61 | 0.56 | 42 | 56 | 98 |
| Isthmian Pacific moist forests | N | 410 | 434 | 4.5 | 1.1 | 39 | 55 | 94 |
| **Talamancan montane forests** |  |  |  |  |  |  |  |  |
| Talamancan and Central montane forests | S1 | 7,175 | 7,593 | 4.5 | 1 | 36 | 59 | 95 |

**NFor** = Nonforest, **For** = Forest, **UPR** = Unprotected, **PR** = Protected, **Type =** upper limit type which Table 4 defines, **d** = superscript d indicates nonforest class includes significant deciduous forest, dry scrub, savanna, or fumarole vegetation, and not all forest absence equates to deforestation.
