# Supplementary material for: Neotropical cloud forests and páramo to contract and dry from declines in cloud immersion and frost
Source: PLoS One. 2019 Apr 17;14(4):e0213155. doi: 10.1371/journal.pone.0213155 (PMC6469753; doi:10.1371/journal.pone.0213155)
Supplement: S15 Table — (DOCX) [file pone.0213155.s020.docx]

S15 Table. TMCF zone forest cover and protection by ecoregion, South America.

| **Ecoregion** | **Type** | **All TMCF**  **Forested Area (km2)** | **All TMCF Zone Area (km2)** | **UPR NFor (%)** | **PR NFor (%)** | **UPR For**  **(%)** | **PR For**  **(%)** | **Total % For** |
| --- | --- | --- | --- | --- | --- | --- | --- | --- |
| **North Central Andes** |  |  |  |  |  |  |  |  |
| Eastern Cordillera real montane forests | S1 | 37,740 | 44,510 | 14 | 0.84 | 57 | 28 | 85 |
| Eastern Cordillera real montane foothills | N | 4,046 | 4,061 | 0.36 | 0.01 | 70 | 29 | 99 |
| **Northern Andes** |  |  |  |  |  |  |  |  |
| Cauca Valley montane forests | S1 | 18,290 | 21,140 | 12 | 1 | 71 | 15 | 86 |
| Cordillera Oriental montane forests | S1 | 10,600 | 11,960 | 11 | 0.81 | 65 | 24 | 89 |
| Cordillera Oriental montane forests north | S1 | 12,690 | 15,450 | 16 | 1.8 | 60 | 22 | 82 |
| Magdalena Valley montane forests | S1 | 40,380 | 52,970 | 22 | 1.5 | 62 | 14 | 76 |
| Northwestern Andean montane forests | S1 | 31,050 | 39,070 | 20 | 0.59 | 67 | 12 | 79 |
| Santa Marta montane forests | S1 | 1,610 | 1,871 | 4.1 | 9.8 | 29 | 57 | 86 |
| Venezuelan Andes montane forests | S1 | 7,461 | 9,276 | 7 | 13 | 31 | 49 | 80 |
| **Northern South America** |  |  |  |  |  |  |  |  |
| Cordillera de la Costa montane forests | N | 1,434 | 1,678 | 5.7 | 8.8 | 18 | 67 | 85 |
| Guajira-Barranquilla xeric scrub^d^ | N | 3 | 10 | 61 | 6.4 | 0 | 30 | 30 |
| Guianan Highlands moist forests | MX | 12,840 | 12,950 | 0.27 | 0.58 | 33 | 66 | 99 |
| Isla Margarita | N | 9 | 10 | 2.9 | 2.2 | 38 | 57 | 95 |
| Lara-Falcon dry forests | N | 69 | 72 | 0.15 | 3.7 | 5.1 | 91 | 96 |
| Paraguana xeric scrub^d^ | N | 1 | 2 | 8.7 | 2.9 | 43 | 39 | 82 |
| **Pacific Coastal** |  |  |  |  |  |  |  |  |
| Ecuadorian dry forests^d^ | N | 365 | 384 | 3.4 | 1.4 | 76 | 20 | 96 |
| Galapagos islands^d^ | N | 419 | 919 | 0 | 54 | 0 | 46 | 46 |
| **Southeast South America** |  |  |  |  |  |  |  |  |
| Alto Paraná forests, Campos Rupestres^d^ | MX | 1,316 | 1,526 | 1.1 | 11 | 4.2 | 82 | 86 |
| Araucaria moist forests^d^ | MX | 1,613 | 2,347 | 27 | 3.9 | 57 | 12 | 69 |
| Serra do Mar coastal forests | N | 230 | 251 | 3.8 | 4.4 | 26 | 66 | 92 |
| **Southern and South-Central Andes** |  |  |  |  |  |  |  |  |
| Bolivian Yungas | S2 | 21,060 | 21,950 | 3.2 | 0.86 | 51 | 45 | 96 |
| Peruvian Yungas | S2 | 37,680 | 40,890 | 7.5 | 0.37 | 71 | 21 | 92 |
| Peruvian Yungas foothills | S2 | 29,040 | 29,660 | 1.5 | 0.56 | 38 | 60 | 98 |
| Southern Andean Yungas^d^ | MX | 22,390 | 28,840 | 20 | 2.8 | 57 | 20 | 77 |

**NFor** = Nonforest, **For** = Forest, **UPR** = Unprotected, **PR** = Protected, **Type =** upper limit type which Table 4 defines, **d** = superscript d indicates nonforest class includes significant deciduous forest, dry scrub, savanna, or fumarole vegetation, and not all forest absence equates to deforestation.
