# Supplementary material for: Neotropical cloud forests and páramo to contract and dry from declines in cloud immersion and frost
Source: PLoS One. 2019 Apr 17;14(4):e0213155. doi: 10.1371/journal.pone.0213155 (PMC6469753; doi:10.1371/journal.pone.0213155)
Supplement: S16 Table — (DOCX) [file pone.0213155.s021.docx]

**S16 Table. Changes in Páramo cloud immersion and frost for RCP 4.5, years 2041-2060.**

With moderate climate change, 70% of Neotropical páramo zone area, including 100% of the páramo zone in Mesoamerica, Venezuela and the Cordillera de Santa Marta of Colombia, will experience declines in cloud immersion, frost, or both as early as around 2040 (2041-2060, average year 2050). These páramo habitats will dry or be subject to tree invasion. Cloud immersion or frost changes are given as percent of total zone area by change category^a^.

| Ecoregion^b^ | UPR, PR, or All^c^ | Páramo Zone Area (km^2^) | RH_d_ < 0%  and Frost < Frost_min2_  (%) | RH_d_ < 0%  (%) | Frost < Frost_min2_  (%) | Frost < Frost_min2_ and MSDF Zone^d^  (%) | Total Affected  (%) | RH_d_ ≥ 0%  Remaining  (%) |
| --- | --- | --- | --- | --- | --- | --- | --- | --- |
| Talamanca | UPR | 6.8 | 100 | - | - | - | 100 | - |
| Talamanca | PR | 111 | 83 | 17 | - | - | 100 | - |
| Santa Marta | UPR | 63.7 | 86 | 14 | - | - | 100 | - |
| Santa Marta | PR | 1,322 | 26 | 74 | - | - | 100 | - |
| Merida | UPR | 709 | 59 | 32 | 8.9 | - | 100 |  |
| Merida | PR | 1,619 | 55 | 37 | 7.6 | - | 100 | 0 |
| N Oriental 1 | UPR | 3,655 | 15 | 9.6 | 58 | - | 83 | 17 |
| N Oriental 1 | PR | 2,613 | 19 | 15 | 33 | - | 67 | 33 |
| N Central/Occid | UPR | 1,660 | 37 | 1.3 | 56 | - | 94 | 4.9 |
| N Central/Occid | PR | 2,082 | 26 | 3.9 | 46 | - | 76 | 24 |
| N Oriental 2 | UPR | 765 | 0 | - | 100 | - | 100 | - |
| N Oriental 2 | PR | 1,663 | 0 | - | 88 | - | 88 | 11 |
| Real | UPR | 8,462 | 0 | 0 | 63 | - | 63 | 37 |
| Real | PR | 5,966 | 0 | 0 | 51 | - | 51 | 49 |
| Central | UPR | 7,659 | 0 | - | 18 | 38 | 56 | 43 |
| Central | PR | 1,993 | - | - | 27 | 39 | 66 | 34 |
| South America | UPR | 22,970 | 7.3 | 2.7 | 46 | 13 | 69 | 31 |
| South America | PR | 17,260 | 13 | 12 | 41 | 4.5 | 71 | 30 |
| Neotropics | UPR | 22,980 | 7.3 | 2.7 | 46 | 13 | 69 | 31 |
| Neotropics | PR | 17,370 | 14 | 12 | 40 | 4.5 | 71 | 29 |
| Santa Marta | Total | 1,386 | 28 | 72 | - | - | 100 | - |
| Merida | Total | 2,328 | 56 | 36 | 8 | - | 100 | 0 |
| N Oriental 1 | Total | 6,268 | 17 | 12 | 48 | - | 77 | 24 |
| N Central/Occid | Total | 3,742 | 31 | 2.7 | 50 | - | 84 | 15 |
| N Oriental 2 | Total | 2,428 | 0 | - | 92 | - | 92 | 7.7 |
| Real | Total | 14,430 | 0 | 0 | 58 | - | 58 | 42 |
| Central | Total | 9,653 | 0 | - | 20 | 39 | 59 | 41 |
| Mesoamerica | Total | 118 | 84 | 16 | - | - | 100 | - |
| South America | Total | 40,230 | 9.9 | 6.6 | 44 | 9.3 | 70 | 31 |
| Neotropics | Total | 40,350 | 10 | 6.6 | 44 | 9.2 | 70 | 30 |

^a^Change categories: RH_d_ < 0% and Frost < Frost_min2_ = Decline in relative humidity (RH) and frost (d·yr^-1^) falls below minimum to be páramo (Frost_min2_)­­; RH_d_ < 0% = Decline in RH; Frost < Frost_min2_ = Frost falls below Frost _min2_; Frost < Frost_min2_ *and* MSDF Zone = Frost falls below Frost _min2_ and adjacent to montane or subalpine dry forest. ^b^See Fig 10 legend for Ecoregions. ^c^UPR=Unprotected; PR=Protected; Total=Unprotected + Protected. ^d^Páramo adjacent to montane or subalpine dry forest will likely be invaded by montane dry forest species.
