# Supplementary material for: Neotropical cloud forests and páramo to contract and dry from declines in cloud immersion and frost
Source: PLoS One. 2019 Apr 17;14(4):e0213155. doi: 10.1371/journal.pone.0213155 (PMC6469753; doi:10.1371/journal.pone.0213155)
Supplement: S17 Table — (DOCX) [file pone.0213155.s022.docx]

**S17 Table. Changes in páramo cloud immersion and frost for RCP 4.5, years 2061-2080.**

With moderate climate change, 81% of Neotropical páramo zone area, including 100% of the páramo zone in Mesoamerica, Venezuela and the Cordillera de Santa Marta of Colombia, will experience declines in cloud immersion, frost, or both as early as around 2060 (2061-2080, average year 2070). These páramo habitats will dry or be subject to tree invasion. Cloud immersion or frost changes are given as percent of total zone area by change category^a^.

| Ecoregion | UPR, PR, or All | Páramo Zone Area (km^2^) | RH_d_ < 0%  and Frost < Frost_min2_  (%) | RH_d_ < 0%  (%) | Frost < Frost_min2_  (%) | Frost < Frost_min2_ and MSDF Zone^b^  (%) | Total Affected  (%) | RH_d_ ≥ 0%  Remaining  (%) |
| --- | --- | --- | --- | --- | --- | --- | --- | --- |
| Talamanca | UPR | 6.8 | 100 | - | - | - | 100 | - |
| Talamanca | PR | 111 | 92 | 7.7 | - | - | 100 | - |
| Santa Marta | UPR | 63.7 | 95 | 5.5 | - | - | 101 | - |
| Santa Marta | PR | 1,322 | 30 | 70 | - | - | 100 | - |
| Merida | UPR | 709 | 75 | 24 | 1.3 | - | 100 |  |
| Merida | PR | 1,620 | 72 | 28 | 0 | - | 100 | - |
| N Oriental 1 | UPR | 3,655 | 43 | 12 | 39 | - | 94 | 6.2 |
| N Oriental 1 | PR | 2,613 | 40 | 37 | 20 | - | 97 | 2.1 |
| N Central/Occid | UPR | 1,660 | 52 | 0 | 45 | - | 97 | 1.2 |
| N Central/Occid | PR | 2,082 | 50 | 4.8 | 28 | - | 83 | 17 |
| N Oriental 2 | UPR | 765 | 4 | - | 96 | - | 100 | - |
| N Oriental 2 | PR | 1,663 | 1.1 | 0 | 95 | - | 96 | 3.5 |
| Real | UPR | 8,462 | 3.7 | 0 | 69 | - | 73 | 27 |
| Real | PR | 5,966 | 3.7 | 0 | 61 | - | 65 | 35 |
| Central | UPR | 7,659 | 0 | 0 | 20 | 51 | 71 | 29 |
| Central | PR | 1,993 | 0 | 0 | 28 | 50 | 78 | 22 |
| South America | UPR | 22,970 | 15 | 2.8 | 45 | 17 | 80 | 20 |
| South America | PR | 17,260 | 23 | 14 | 40 | 5.8 | 83 | 18 |
| Neotropics | UPR | 22,980 | 15 | 2.8 | 45 | 17 | 80 | 20 |
| Neotropics | PR | 17,370 | 23 | 14 | 39 | 5.8 | 82 | 17 |
| Santa Marta | Total | 1,386 | 33 | 67 | - | - | 100 | - |
| Merida | Total | 2,329 | 73 | 27 | 0 | - | 100 | - |
| N Oriental 1 | Total | 6,268 | 42 | 23 | 31 | - | 96 | 4.5 |
| N Central/Occid | Total | 3,742 | 51 | 3.1 | 35 | - | 89 | 10 |
| N Oriental 2 | Total | 2,428 | 2 | 0 | 96 | - | 98 | 2.4 |
| Real | Total | 14,430 | 3.7 | 0 | 66 | - | 70 | 30 |
| Central | Total | 9,653 | 0 | 0 | 22 | 51 | 73 | 27 |
| Mesoamerica | Total | 118 | 93 | 7.3 | - | - | 100 | - |
| South America | Total | 40,230 | 18 | 7.8 | 43 | 12 | 81 | 19 |
| Neotropics | Total | 40,350 | 18 | 7.8 | 43 | 12 | 81 | 19 |

^a^Change categories: RH_d_ < 0% and Frost < Frost_min2_ = Decline in relative humidity (RH) and frost (d·yr^-1^) falls below minimum to be páramo (Frost_min2_)­­; RH_d_ < 0% = Decline in RH; Frost < Frost_min2_ = Frost falls below Frost _min2_; Frost < Frost_min2_ *and* MSDF Zone = Frost falls below Frost _min2_ and adjacent to montane or subalpine dry forest. ^b^See Fig 10 legend for Ecoregions. ^c^UPR=Unprotected; PR=Protected; Total=Unprotected + Protected. ^d^Páramo adjacent to montane or subalpine dry forest will likely be invaded by montane dry forest species.
