# Supplementary material for: Neotropical cloud forests and páramo to contract and dry from declines in cloud immersion and frost
Source: PLoS One. 2019 Apr 17;14(4):e0213155. doi: 10.1371/journal.pone.0213155 (PMC6469753; doi:10.1371/journal.pone.0213155)
Supplement: S18 Table — (DOCX) [file pone.0213155.s023.docx]

**S18 Table. Changes in Páramo cloud immersion and frost for RCP 8.5, years 2041-2060.**

In a worst-case scenario, 86% of Neotropical páramo zone area, including 100% of the páramo zone in Mesoamerica, and nearly the entire Andean Cordillera Oriental of Colombia and Venezuela, will experience declines in cloud immersion, frost, or both as early as around 2040 (2041-2060, average year 2050). These páramo habitats will dry or be subject to tree invasion. Cloud immersion or frost changes are given as percent of total zone area by change category^a^.

| Ecoregion | UPR, PR, or All | Páramo Zone Area (km^2^) | RH_d_ < 0%  and Frost < Frost_min2_  (%) | RH_d_ < 0%  (%) | Frost < Frost_min2_  (%) | Frost < Frost_min2_ and MSDF Zone^b^  (%) | Total Affected  (%) | RH_d_ ≥ 0%  Remaining  (%) |
| --- | --- | --- | --- | --- | --- | --- | --- | --- |
| Talamanca | UPR | 6.8 | 100 | - | - | - | 100 | - |
| Talamanca | PR | 111 | 94 | 5.7 | - | - | 100 | - |
| Santa Marta | UPR | 63.7 | 97 | 2.6 | - | - | 100 | - |
| Santa Marta | PR | 1,322 | 34 | 66 | - | - | 100 | - |
| Merida | UPR | 709 | 83 | 17 | - | - | 100 |  |
| Merida | PR | 1,620 | 78 | 22 | - | - | 100 | - |
| N Oriental 1 | UPR | 3,655 | 79 | 21 | - | - | 100 | - |
| N Oriental 1 | PR | 2,613 | 57 | 43 | - | - | 100 | - |
| N Central/Occid | UPR | 1,660 | 67 | 0 | 31 | - | 98 | 1 |
| N Central/Occid | PR | 2,082 | 63 | 5.1 | 15 | - | 83 | 17 |
| N Oriental 2 | UPR | 765 | 100 | - | 0 | - | 100 | - |
| N Oriental 2 | PR | 1,663 | 95 | 4.3 | 0 | - | 99 | - |
| Real | UPR | 8,462 | 21 | 8.8 | 53 | - | 83 | 16 |
| Real | PR | 5,966 | 30 | 13 | 37 | - | 80 | 21 |
| Central | UPR | 7,659 | 0 | - | 20 | 52 | 72 | 27 |
| Central | PR | 1,993 | 1 | 0 | 27 | 52 | 80 | 20 |
| South America | UPR | 22,970 | 32 | 7.2 | 29 | 17 | 85 | 15 |
| South America | PR | 17,260 | 46 | 19 | 18 | 6 | 89 | 11 |
| Neotropics | UPR | 22,980 | 32 | 7.2 | 29 | 17 | 85 | 15 |
| Neotropics | PR | 17,370 | 46 | 19 | 18 | 5.9 | 89 | 11 |
| Santa Marta | Total | 1,386 | 37 | 63 | - | - | 100 | - |
| Merida | Total | 2,329 | 79 | 21 | - | - | 100 | - |
| N Oriental 1 | Total | 6,268 | 70 | 30 | - | - | 100 | - |
| N Central/Occid | Total | 3,742 | 65 | 3.3 | 22 | - | 90 | 9.8 |
| N Oriental 2 | Total | 2,428 | 97 | 3 | 0 | - | 100 | - |
| Real | Total | 14,430 | 25 | 11 | 47 | - | 83 | 18 |
| Central | Total | 9,653 | 0 | 0 | 21 | 52 | 73 | 26 |
| Mesoamerica | Total | 118 | 95 | 5.4 | - | - | 100 | - |
| South America | Total | 40,230 | 38 | 12 | 24 | 13 | 87 | 14 |
| Neotropics | Total | 40,350 | 38 | 12 | 24 | 12 | 86 | 14 |

^a^Change categories: RH_d_ < 0% and Frost < Frost_min2_ = Decline in relative humidity (RH) and frost (d·yr^-1^) falls below minimum to be páramo (Frost_min2_)­­; RH_d_ < 0% = Decline in RH; Frost < Frost_min2_ = Frost falls below Frost _min2_; Frost < Frost_min2_ *and* MSDF Zone = Frost falls below Frost _min2_ and adjacent to montane or subalpine dry forest. ^b^See Fig 10 legend for Ecoregions. ^c^UPR=Unprotected; PR=Protected; Total=Unprotected + Protected. ^d^Páramo adjacent to montane or subalpine dry forest will likely be invaded by montane dry forest species.
