# Supplementary material for: Neotropical cloud forests and páramo to contract and dry from declines in cloud immersion and frost
Source: PLoS One. 2019 Apr 17;14(4):e0213155. doi: 10.1371/journal.pone.0213155 (PMC6469753; doi:10.1371/journal.pone.0213155)
Supplement: S19 Table — (DOCX) [file pone.0213155.s024.docx]

**S19 Table. Changes in Páramo cloud immersion and frost for RCP 8.5, years 2061-2080.**

In a worst-case scenario, 98% of Neotropical páramo zone area, including 100% of the páramo zone in Mesoamerica and the entire Andean Cordillera Oriental of Colombia and Venezuela, will experience declines in cloud immersion, frost, or both as early as around 2060 (2061-2080, average year 2070). These páramo habitats will dry or be subject to tree invasion. Cloud immersion or frost changes are given as percent of total zone area by change category^a^.

| Ecoregion | UPR, PR, or All | Páramo Zone Area (km^2^) | RH_d_ < 0%  and Frost < Frost_min2_  (%) | RH_d_ < 0%  (%) | Frost < Frost_min2_  (%) | Frost < Frost_min2_ and MSDF Zone^b^  (%) | Total Affected  (%) | RH_d_ ≥ 0%  Remaining  (%) |
| --- | --- | --- | --- | --- | --- | --- | --- | --- |
| Talamanca | UPR | 6.8 | 100 | - | - | - | 100 | - |
| Talamanca | PR | 111 | 100 | - | - | - | 100 | - |
| Santa Marta | UPR | 63.7 | 100 | - | - | - | 100 | - |
| Santa Marta | PR | 1,322 | 48 | 52 | - | - | 100 | - |
| Merida | UPR | 709 | 98 | 2.4 | - | - | 100 |  |
| Merida | PR | 1,620 | 92 | 8.4 | - | - | 100 | - |
| N Oriental 1 | UPR | 3,655 | 94 | 5.6 | - | - | 100 | - |
| N Oriental 1 | PR | 2,613 | 73 | 27 | - | - | 100 | - |
| N Central/Occid | UPR | 1,660 | 70 | 0 | 30 | - | 100 | - |
| N Central/Occid | PR | 2,082 | 66 | 2.5 | 23 | - | 92 | 8.7 |
| N Oriental 2 | UPR | 765 | 100 | - | 0 | - | 100 | - |
| N Oriental 2 | PR | 1,663 | 100 | - | 0 | - | 100 | - |
| Real | UPR | 8,462 | 38 | 2.2 | 59 | - | 99 | 1.2 |
| Real | PR | 5,966 | 60 | 4.9 | 31 | - | 96 | 3.7 |
| Central | UPR | 7,659 | 6.5 | 0 | 16 | 70 | 93 | 7.9 |
| Central | PR | 1,993 | 15 | 0 | 14 | 69 | 98 | 1.8 |
| South America | UPR | 22,970 | 43 | 1.8 | 29 | 23 | 97 | 3 |
| South America | PR | 17,260 | 64 | 11 | 15 | 8 | 98 | 2.6 |
| Neotropics | UPR | 22,980 | 43 | 1.8 | 29 | 23 | 97 | 3 |
| Neotropics | PR | 17,370 | 64 | 11 | 15 | 7.9 | 98 | 2.5 |
| Santa Marta | Total | 1,386 | 51 | 49 | - | - | 100 | - |
| Merida | Total | 2,329 | 93 | 6.6 | - | - | 100 | - |
| N Oriental 1 | Total | 6,268 | 85 | 15 | - | - | 100 | - |
| N Central/Occid | Total | 3,742 | 68 | 1.4 | 26 | - | 95 | 4.8 |
| N Oriental 2 | Total | 2,428 | 100 | - | 0 | - | 100 | - |
| Real | Total | 14,430 | 47 | 3.3 | 47 | - | 97 | 2.2 |
| Central | Total | 9,653 | 8.3 | 0 | 16 | 69 | 93 | 6.6 |
| Mesoamerica | Total | 118 | 100 | - | - | - | 100 | - |
| South America | Total | 40,230 | 52 | 5.7 | 23 | 17 | 98 | 2.8 |
| Neotropics | Total | 40,350 | 52 | 5.7 | 23 | 17 | 98 | 2.8 |

^a^Change categories: RH_d_ < 0% and Frost < Frost_min2_ = Decline in relative humidity (RH) and frost (d·yr^-1^) falls below minimum to be páramo (Frost_min2_)­­; RH_d_ < 0% = Decline in RH; Frost < Frost_min2_ = Frost falls below Frost _min2_; Frost < Frost_min2_ *and* MSDF Zone = Frost falls below Frost _min2_ and adjacent to montane or subalpine dry forest. ^b^See Fig 10 legend for Ecoregions. ^c^UPR=Unprotected; PR=Protected; Total=Unprotected + Protected. ^d^Páramo adjacent to montane or subalpine dry forest will likely be invaded by montane dry forest species.
